# Label-Free Quantitative Thermal Proteome Profiling Reveals Target Transcription Factors with Activities Modulated by MC3R Signaling

**DOI:** 10.1021/acs.analchem.3c03643

**Published:** 2023-10-07

**Authors:** Friederike A. Sandbaumhüter, Mariya Nezhyva, Per E. Andrén, Erik T. Jansson

**Affiliations:** † Department of Pharmaceutical Biosciences, Uppsala University, 751 24 Uppsala, Sweden; ‡ Science for Life Laboratory, Spatial Mass Spectrometry, Uppsala University, 751 24 Uppsala, Sweden

## Abstract

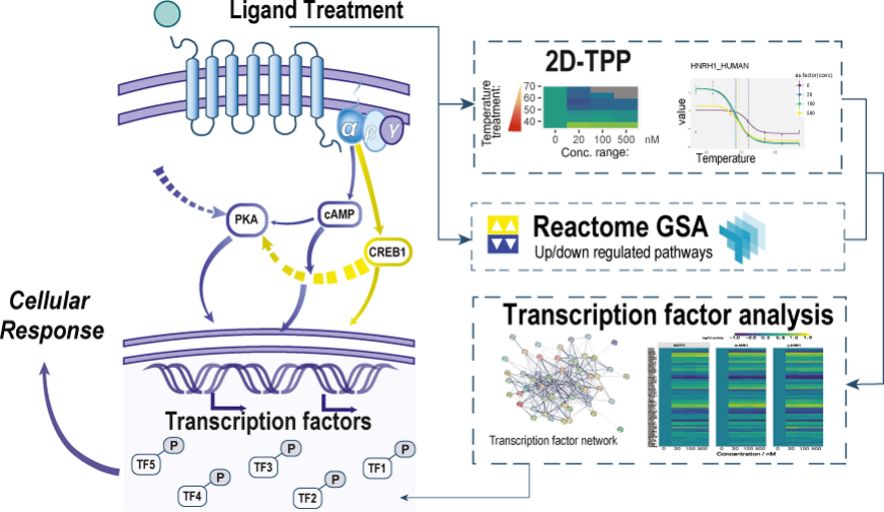

Thermal proteome profiling with label-free quantitation using ion-mobility-enhanced LC–MS offers versatile data sets, providing information on protein differential expression, thermal stability, and the activities of transcription factors. We developed a multidimensional data analysis workflow for label-free quantitative thermal proteome profiling (TPP) experiments that incorporates the aspects of gene set enrichment analysis, differential protein expression analysis, and inference of transcription factor activities from LC–MS data. We applied it to study the signaling processes downstream of melanocortin 3 receptor (MC3R) activation by endogenous agonists derived from the proopiomelanocortin prohormone: ACTH, α-MSH, and γ-MSH. The obtained information was used to map signaling pathways downstream of MC3R and to deduce transcription factors responsible for cellular response to ligand treatment. Using our workflow, we identified differentially expressed proteins and investigated their thermal stability. We found in total 298 proteins with altered thermal stability, resulting from MC3R activation. Out of these, several proteins were transcription factors, indicating them as being downstream target regulators that take part in the MC3R signaling cascade. We found transcription factors CCAR2, DDX21, HMGB2, SRSF7, and TET2 to have altered thermal stability. These apparent target transcription factors within the MC3R signaling cascade play important roles in immune responses. Additionally, we inferred the activities of the transcription factors identified in our data set. This was done with Bayesian statistics using the differential expression data we obtained with label-free quantitative LC–MS. The inferred transcription factor activities were validated in our bioinformatic pipeline by the phosphorylated peptide abundances that we observed, highlighting the importance of post-translational modifications in transcription factor regulation. Our multidimensional data analysis workflow allows for a comprehensive characterization of the signaling processes downstream of MC3R activation. It provides insights into protein differential expression, thermal stability, and activities of key transcription factors. All proteomic data generated in this study are publicly available at DOI: 10.6019/PXD039945.

## Introduction

Protein–ligand interactions play a critical role in nearly all biological processes, making their study essential for understanding cellular functions and developing therapeutics. Numerous methods have been developed to characterize these interactions, typically focusing on the ligand affinity. However, it is important to follow-up protein–ligand interactions with their downstream effects, including transcriptomic, proteomic, and post-translational modification (PTM) changes.

Thermal proteome profiling (TPP) has emerged as a valuable technique to gain insights into protein function, protein–protein interactions, and even forecast adverse drug effects in physiologically relevant environments.^1,2^ TPP is based on the intrinsic property of a protein–ligand interaction, for instance, when ligand binding stabilizes the protein structure and thus increases its melting temperature.^1,2^ In detail, TPP utilizes a multistep approach, including ligand treatment, heating, extraction, purification, digestion, and LC–MS analysis. This comprehensive method generates complex raw data, from which melting curves and relative protein solubility measurements can be obtained.^1−6^ The most common 2D-TPP procedure involves treating cells with a drug at various concentrations before heating over a range of temperatures. From the resulting complex data set, dose response curves are obtained based on relative protein solubility measurements. Notably, this approach was used to study the effect of adenosine triphosphate binding on protein stability and solubility and to identify phenylalanine hydroxylase as an off-target of the drug panobinostat.^3,4^ After tryptic digestion of the proteins, samples can be isobarically labeled for multiplexed data acquisition.^1−6^ However, a label-free quantitative workflow can also benefit TPP. Omitting the labeling step allows one to follow changes in protein expression levels while retaining information about protein stability. Hence, with label-free quantitation, more information is obtained from a single sample set and allows the user to create a multilayered information data set, previously shown for TPP workflows.^7−10^

**Figure 1:**
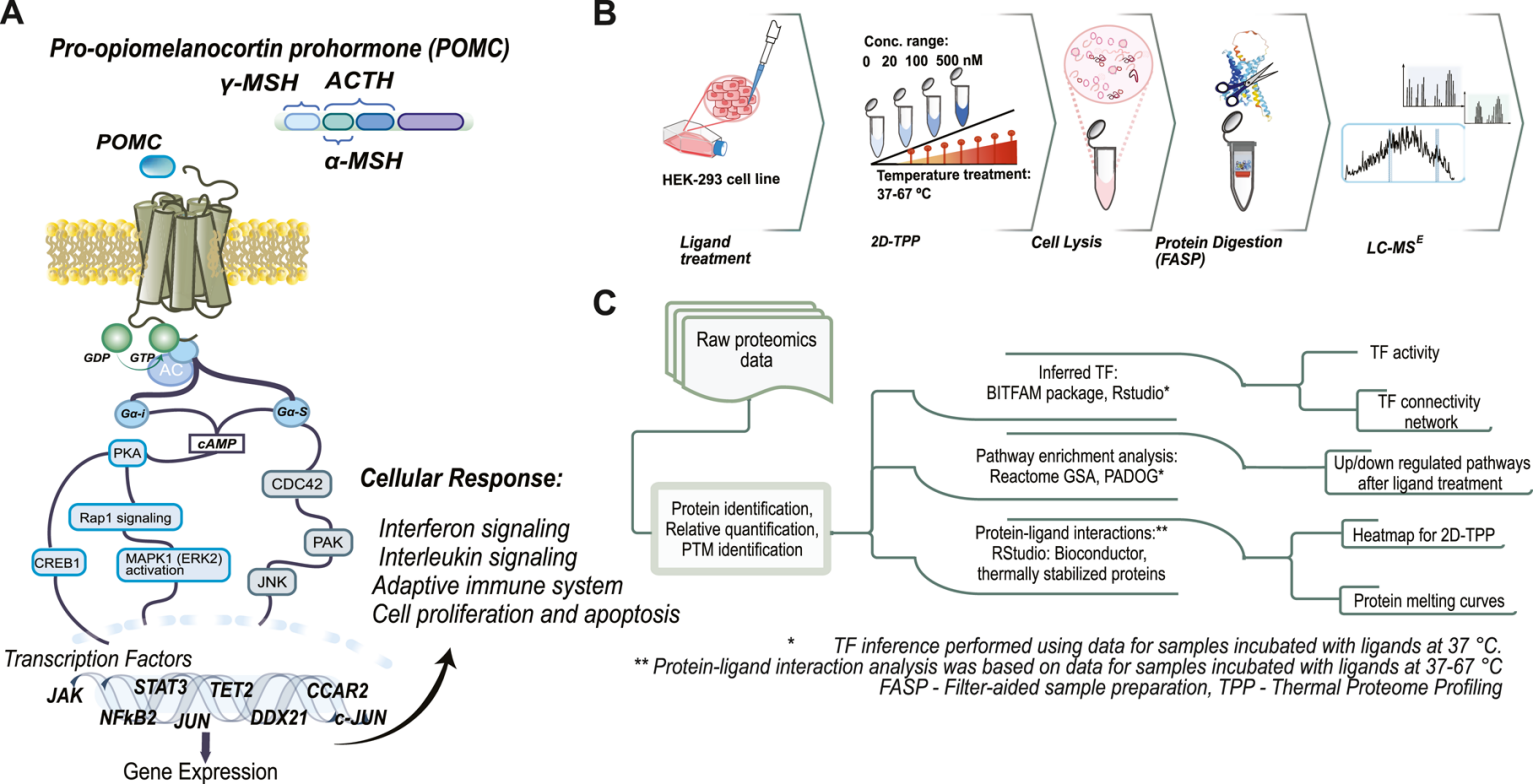
Overview of the preparatory and analytical workflows. (A) POMC derived ligands and their downstream signaling cascades. (B) Schematic overview of the thermal proteome profiling (TPP) workflow. MC3R-expressing HEK293 cells were treated with ACTH, α-MSH, or γ-MSH at concentrations of 20, 100, and 500 nM or with DMSO as a vehicle-only negative control. (C) Schematic overview of the TPP data analysis workflow. Protein identification and relative quantification were achieved by direct analysis of the raw LC–MS data, after which various bioinformatics tools were used to infer changes in transcription factor (TF) activity, perform enriched pathway analysis, and identify thermally affected proteins.

Herein, we apply an integrated data analysis workflow to link alterations in thermal stability with specific biological pathways and infer activities of transcription factors downstream of the ligand-mediated activation of the melanocortin 3 receptor (MC3R). MC3R is a transmembrane G-protein coupled receptor (GPCR) that mediates diverse biological processes, including immune responses, inflammatory control, and energy homeostasis (Figure 1A).^11−18^ MC3R is activated by its endogenous agonists, adrenocorticotropic hormone (ACTH) and α- and γ-melanocyte stimulating hormones (MSH) which are formed by tissue-specific post-translational processing of pro-opiomelanocortin (POMC). The common key pharmacophore His-Phe-Arg-Trp is essential for binding and activation of the receptor. The binding of agonists to MC3R increases cellular cAMP levels and thus triggers an anti-inflammatory response. This involves suppression of inflammatory mediators such as cytokines, the protection of cells from inflammation-related damage and death. These effects have been studied extensively in the case of the agonists ACTH and α-MSH, but the responses induced by γ-MSH are much less characterized.^12,13,15,17^ MC3R is the only MCR that responds to physiological levels of γ-MSH.^12^

We are the first to present a data analysis pipeline for label-free quantitative TPP experiments that incorporates gene set enrichment analysis, differential protein expression analysis, and inference of transcription factor activities. Our TPP experiments use a data-independent acquisition (DIA) protocol in preference to a widely used data-dependent acquisition (DDA) approach for TPP (previously described^4−8^ elsewhere). Next, we demonstrate that our bioinformatics workflow is capable of yielding a wealth of versatile information from a single proteomics data set. It was applied to map the signaling pathways downstream of MC3R and to deduce transcription factors responsible for the cellular response to ligand treatment.

## Materials and Methods

### Chemicals and Reagents

Gibco Dulbecco’s Modified Eagle Medium, high glucose (DMEM), PierceTM Trypsin protease MS grade, protease inhibitor, DMSO, Corning Premium Fetal Bovine Serum (FBS), Invitrogen accutase, phosphate buffered saline (PBS), doxycycline, isobutyl-1-methylxanthine (IBMX), adrenocorticotropin hormone (ACTH), α-melanocyte stimulating hormone (α-MSH), γ-melanocyte stimulating hormone (γ-MSH), UPLC–MS grade water, acetonitrile, and formic acid were purchased from Thermo Fisher Scientific (Waltham, MA). Dithiothreitol (DTT), iodoacetamide (IAA), HeLa digest, 4-(3-butoxy-4-methodybenzyl) imidazolidone (Ro 20-1724), and HEPES were obtained from Sigma-Aldrich Chemie (Steinheim, Germany). Urea and NH_4_HCO_3_ were obtained from Acros Organics (Geel, Belgium). Tris(hydroxymethyl)aminomethane hydrochloride (Tris), LC–MS grade water, acetonitrile, and formic acid were obtained from Fisher Scientific (Geel, Belgium). RapiGest SF Surfactant was purchased from Waters Corporation (Milford, MA). The cAMP-GloTM Assay including cAMP-GloTM Lysis Buffer, cAMP-GloTM reaction buffer, Protein Kinase A, Kinase-Glo substrate, and Kinase-Glo Buffer were from Promega Corporation (Madison, WI). Stock solutions of all ligands were prepared in 0.1 mM DMSO.

### Cell Culture and Ligand Treatment

A human embryonic kidney 293 cell line transfected with a tetracycline-regulated expression system to overexpress MC3R (HEK-TREx-MC3R) was provided by Astra Zeneca. Cells were cultured in DMEM supplemented with 10% FBS (growth medium) at 37 °C in a 5% CO_2_ environment. For the cAMP assay, cells were seeded at a cell density of 10 000 cells/well into a 96-well plate. For the TPP experiment, 2.1 × 10^6^ cells were seeded into a T-75 flask. MC3R overexpression was induced by replacing the growth medium with an induction medium consisting of growth media containing 0.01 nM doxycycline at 80% cell confluency in T-75 flask (1 × 10^6^ cells/mL). After 24 h, the induction medium was removed, and the cells were incubated in the treatment medium for the cAMP assay or the TPP experiment.

### cAMP Assay

The presence and functionality of MC3R after induced overexpression were verified using the cAMP-GloTM Assay (Promega Corporation, Madison, WI). After the cells were prepared as described above, the doxycyclin-containing medium was replaced with a treatment solution containing 500 μM IBMX and 100 μM Ro 20-1724 as well as 0, 0.5, 5, 50, or 500 nM of γ-MSH, or 4 μM cAMP in PBS as a positive control. The plate was then incubated at 37 °C for 1 h, after which the assay was performed according to the manufacturer’s protocol. Briefly, cell lysis was followed by incubation with a cAMP detection solution containing Protein Kinase A at room temperature for 20 min and then with Kinase-Glo Reagent at room temperature for 10 min. Finally, the luminescence was measured using a FLUOstar Omega instrument (BMG Labtech, Ortenberg, Germany).

### Thermal Proteome Profiling Experiment

The treatment solutions for the TPP experiment contained either no additives or ACTH, α-MSH, and γ-MSH at a concentration of 20, 100, or 500 nM. Cells were incubated in the treatment solutions for 1 h (37 °C, 5% CO_2_), then detached with accutase and washed three times with ice-cold PBS. Next, ice-cold PBS containing a protease inhibitor (10 μL/mL) was added to give a final cell concentration of 4 × 10^6^ cells/100 μL. Seven aliquots of 100 μL each were centrifuged at 300*g* for 3 min (4 °C) and kept on ice until further use. Each aliquot was then heated on a heating block (Thermomixer Compact, Eppendorf) for 3 min at one of the following temperatures: *T* ∈ [37, 42, 47, 52, 57, 62, 67] °C before being allowed to cool to room temperature over 3 min. After cooling, the samples were snap-frozen in liquid nitrogen and stored on ice.

### Lysis, Sample Purification, and Protein Digestion

RapiGest was added to the samples (1 μL/100 μL). Cells were lyzed by performing two freeze–thaw cycles in which they were frozen in liquid nitrogen and thawed in a heating block at 25 °C according to a previously reported procedure.^4^ After each heating step, the samples were vortexed. The samples were then centrifuged at 14 000*g* at 4 °C for 90 min to separate the soluble protein fraction from the aggregated proteins, and the supernatant was transferred into new tubes. Finally, the protein concentration was measured with a Nanodrop instrument (Thermo Scientific, Waltham, MA). Samples were kept on ice.

Sample purification and protein digestion were done by filter-aided sample preparation as described previously.^19^ Briefly, 20 μg of total protein was placed on centrifugal filter units (Microcon-30 kDa; Merck, Darmstadt, Germany) that had been preconditioned with 1% formic acid. Samples were washed with urea buffer (8 M urea and 100 mM Tris (pH 8.5)) on the filter and then centrifuged at 14 000*g* at 4 °C for 15 min. Sample reduction was performed with 8 mM DTT at 56 °C for 15 min, followed by alkylation with 50 mM IAA at room temperature for 20 min. Excess IAA was then removed in a second incubation with 8 mM DTT. An intermittent washing step with urea buffer (8 M urea and 100 mM Tris (pH 8.5)) was performed twice after each incubation. Before tryptic digestion (enzyme-protein ratio 1:50 (w/w)) the samples were washed with 50 mM NH_4_HCO_3_ three times and centrifuged at 14 000*g* for 10 min each. Trypsin digestion was performed overnight (16 h) in a wet chamber at 37 °C, and the digested peptides were collected by washing the filter with 50 mM NH_4_HCO_3_ and centrifuging twice at 14 000*g* for 10 min before adding trifluoroacetic acid to a final concentration of 1% (v/v). Samples were then dried in a speedvac (Concentrator 5301, Eppendorf) at 45 °C and reconstituted in a solution of 3% acetonitrile and 0.1% formic acid in water (protein concentration: 150 ng/μL).

### Proteomic Sample Analysis with LC–MS

Tryptic peptides were analyzed with a nanoAcquity UPLC system coupled to a Synapt G2-Si HDMS mass spectrometer with a nanoelectrospray ionization source (Waters Corporation, Manchester, UK). The nanoAcquity UPLC system consisted of a C18, 5 μm, 180 μm × 20 mm trap column and an HSS-T3 C18 1.8 μm, 75 μm × 250 mm analytical column (Waters Corporation, Manchester, UK) set to trapping mode. Samples containing 300 ng of protein were injected in each run. Mobile phases A and B consisted of 0.1% formic acid and 3% dimethyl sulfoxide in water (v/v) and 0.1% formic acid and 3% dimethyl sulfoxide in acetonitrile (v/v), respectively. Peptide separation was done using a gradient from 3% to 40% (v/v) of mobile phase B at a constant flow rate of 0.3 μL/min over 120 min. Lock-mass correction was performed by spraying a lock-mass solution containing [Glu1]-fibrinopeptide B (0.1 μM) and leu-enkephalin (1 μM) through the reference channel every 60 s. Data were acquired using a data-independent acquisition workflow in positive ion mode with a UDMS^E^ method.^19−21^ The system’s performance and stability were monitored by injecting a commercially available HeLa digest (Thermo Scientific, Waltham, MA) after every seventh sample injection.

### ProteinLynx Global Server (PLGS)

Raw data were processed using PLGS 3.0.3 (Waters Corporation, Milford, MA, USA) and the SWISSPROT Human database (UniProtK version 12/10/2021). The values of key analytical parameters were set as follows: the false discovery rate (FDR) was set to 0.01, the digestion reagent was Trypsin, and the number of peptide missed cleavages was set to 1. The minimum number of fragment ion matches was set to 1 per peptide and 3 per protein, and the minimum number of peptide matches was set to 2 per protein. Carbamidomethyl cysteine was set as a fixed modification, and lysine acetylation, C-terminal amidation, asparagine deamidation, glutamine deamidation, and methionine oxidation were set as variable modifications. Additional analyses were performed by conducting searches with phosphorylation at serine, threonine, and tyrosine as variable modifications using the results for samples incubated with the ligands at 37 °C. For phosphopeptide analysis, an individual peptide needed at least 3 fragments to be accepted for further data analysis. The detailed PLGS processing and workflow parameters are provided in Tables S1 and S2.

### Label-Free Quantification with ISOQuant

ISOQuant 1.8 workflow uses PLGS identifications, intensity normalization, and protein isoform and homology filtering to annotate signal clusters based on accurate mass data and retention and drift times.^20,21^ This approach maximizes the recovery of inferred protein abundances for relative protein quantification using the TOP3 method, in which quantification is based on the average intensity of the three most intense peptides of each protein. The software settings used for this purpose are specified in the appended ISOquant report files (Supporting Information files Data TPP_ACTH, TPP_alpha, and TPP_gamma).

### Analysis of Thermal Protein Profiling Data

Thermally stabilized or destabilized proteins were identified by adapting a previously reported algorithm for analysis of isobarically labeled samples.^4^ Briefly, the protein abundance *A*_*c*,*p*,*T*_ for a drug concentration *c*, temperature *T*, and protein ID *p* obtained from the ISOQuant analysis using the TOP3 method was log_2_-transformed such that
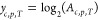
1Next, the average transformed abundance per protein and temperature at the null concentration was subtracted from each individual value:
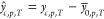
2

The above operation centered the  around zero. The resulting data were then antilog-transformed, causing  to become centered around unity. Next, the data were analyzed to evaluate the concentration dependence of the protein melting curves. A filter criterion was used to retain only those temperatures where the fold-change in protein abundance within the concentration series was above or below a predefined threshold (*h* = 1.5) for further analysis, meaning that the maximum fold change for a protein ϕ_*T*_^*max*^ was required to satisfy the following equation:
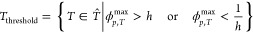
3

In analogy to previous work,^4^ for each protein *p*, our null hypothesis *H*_0_ is that the soluble fraction  is independent of the concentration *c* of the drug with which the cells were treated. Therefore, if there is no direct or indirect interaction between the drug and the protein, then
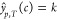
4However, if there is an interaction, the soluble fraction can be described by a 4-parameter log–logistic function such that our alternate hypothesis *H*_1_ is given by
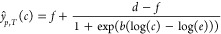
5

The functions for *H*_0_ and *H*_1_ were fitted for the full concentration range of each protein at each of the incubation temperatures. Since our analysis used data obtained by label-free quantitation, we were not limited to a restricted number of isobaric channels and had no need to worry about batch effects because each sample was analyzed individually. This increases the number of degrees of freedom for the curve-fitting analysis because the relative abundance at the null concentration is centered around unity rather than fixed to unity. The residual sum of squares between the linear model and the log–logistic function was then evaluated to determine whether the data were better explained with the *H*_1_ model or the *H*_0_ model. The *F*-statistic for a given temperature condition was obtained as
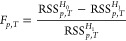
6

The combined *F*-statistic *F*_*p*_^comb^ across all temperatures was then obtained as
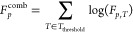
7We also followed the suggestions of Sridharan et al.^4^ and assessed the false discovery rate using a bootstrap method. For this purpose, after the *F*-statistic had been evaluated on the original data, the melting curve data were shuffled, and *H*_0_ and *H*_1_ models were fitted to the shuffled data. *F*-statistics were then evaluated for the shuffled data and joined with the original data, and this procedure was repeated 100 times. The results for all 100 runs were then ranked by their *F*-statistic, with the highest score on top. The average FDR obtained from the 100 bootstrap runs was then computed as
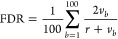
8Here, *r* denotes the number of hits obtained within the original data set and *v*_*b*_ is the number of hits within the permuted data set *b*.^4,22^ We used a criterion of FDR = 0.1 to retain identified proteins exhibiting altered thermal stability.

### Pathway Analysis

The label-free quantitation data were used for multidata set Pathway Analysis with Down-weighting of Overlapping Genes (PADOG) in the Reactome GSA software. The multiple data sets in this case were triplicates of the results obtained for each condition (concentration and ligand) in relation to the results obtained for control samples (DMSO treatment).

### Transcription Factor Analysis

Transcription factor activity was inferred from the combined data set of identified proteins with log-transformed intensity values using Bayesian inference transcription factor activity modeling as implemented in the Bayesian inference transcription factor activity modeling (BITFAM) R-package.^23^

## Results and Discussion

### 2D-TPP analysis of MC3R Interaction with Endogeneous Ligands

To characterize the systemic responses of MC3R activation and identify ligand-specific and concentration-dependent effects, we cultured HEK293 cells stably transfected with MC3R and stimulated them with the endogenous agonists ACTH, α-MSH, or γ-MSH at concentrations between 0 and 500 nM for 1 h (Figure 1A,B). The HEK293 cell line has previously been used to study MCRs^24−26^ and is a robust model system with stable transfection. This enables induced overexpression of target proteins to study the intracellular signaling cascades of single receptors. The presence and functionality of the overexpressed receptor were confirmed using a cAMP assay. Briefly, binding of an agonist to MC3R is followed with formation of cAMP. In this assay we measured luminescence of oxyluciferin, which is inversely proportional to the concentration of cAMP. As expected, the luminescence decreased in a dose-dependent manner upon incubating the cells with 0.5, 5, 50, or 500 nM γ-MSH, indicating that the interaction between γ-MSH and MC3R caused cAMP production to increase in parallel with the ligand concentration (Figure S1).

The treated cells were then heated to temperatures between 37 and 67 °C and lyzed. Next, the soluble protein fraction was prepared for label-free bottom-up proteomic analysis using LC–MS. Individual LC–MS runs were performed for all 3 agonists at 7 temperatures and 3 concentrations with 3 replicates. In addition, a set of DMSO (vehicle only) runs were performed at the same 7 temperatures with 3 replicates, giving 210 runs in total. A key advantage of our label-free quantitative approach, when compared with the more common isobaric labeling workflow, is that fewer sample aliquots must be processed to capture the information needed for differential expression analysis. To monitor the performance of the LC–MS system and evaluate the robustness of the analysis, we performed periodic injections of commercial tryptic digests of HeLa cell lysates between LC–MS analyses of TPP aliquots. Over the duration of the experiment, 83% of all proteins detected in these HeLa standard runs had RSD values below 0.05 (Figures S2 and S3). Across all ligands and concentrations, a total of 3318 protein identities were inferred from the data set obtained by untargeted MS-based proteomic analysis of samples in the TPP experiments. An overview of the identified proteins and phosphopeptides is given in Figures S5 and S6.

### Workflow of Data Acquisition and Bioinformatic Pipeline

TPP offers valuable insights into protein thermal stability and its modulation by ligands. When choosing a suitable data acquisition strategy between DDA and DIA for TPP, significant differences arise, influencing data analysis and interpretation. DIA’s label-free analysis eliminates the need for isobaric labeling, thereby allowing individual examination of each sample. The use of retention time and ion mobility alignment facilitates collation of data from individual runs for label-free quantification.^20,21,27,28^ In turn, this reduces missing values and preserves variation under control conditions.^20,21,28−31^ Moreover, DIA workflows preserve raw data for comprehensive analyses, which is crucial for studying ligand-induced changes in thermal stability and differential expression. In contrast, the iTRAQ-DDA workflow permits simultaneous analysis of multiple samples in a single LC–MS run but mandates isobaric labels, leading to complex sample handling suitable while using Orbitrap instruments.

In this work, data-independent acquisition allowed us to develop a bioinformatic workflow (Figure 1C) that combines information on thermal proteome stability with differential expression analysis. This approach enables the monitoring of the relationship between protein abundance and thermal stability, thereby identifying proteins that exhibit significant changes in both expression levels and thermal stability under different experimental conditions. Within this workflow, differential expression information plays a crucial role, particularly in the context of transcription factor analysis using BITFAM.^23^ Transcription factors serve as key regulators of gene expression and exert control over numerous downstream target genes. By inferring the activity of transcription factors based on the expression patterns of these genes, valuable insights into the regulatory networks that drive observed biological processes can be realized. This technique, which was previously applied to RNA-seq data,^23^ uses a Chip-Seq TF database to decompose log_2_-transformed activities into a product of 2 matrices with values representing transcription factor activities and the connectivity of each transcription factor to potential target genes.

Additionally, the data have been specifically examined for phosphorylation as a key PTM. PTMs offer a vital layer of biological information, connecting changes in thermal stability to regulatory functions, including transcription factor activities. By targeting phosphorylation, proteins that play a pivotal role in biological regulation but may not exhibit significant changes in differential expression are not overlooked. This enriched analysis enhances our overall understanding of complex interactions within the biological system.

Furthermore, the integration of all available information from the data set, including differential expression, phosphorylation modification, thermal stability, and transcription factor activity, facilitated the identification of proteins relevant to MC3R response to endogenous ligand treatment. This comprehensive approach illustrates the intricate relationship between thermal stability and protein regulation, offering a unified perspective on the complex biological system being studied.

### Ligand-Induced Effects on Protein Thermal Stability and Phosphorylation

In the TPP experiments, we analyzed the soluble protein fractions remaining in the samples after incubation of intact HEK293 cells overexpressing MC3R with the studied ligands at various temperatures. Proteins may be thermally stabilized or destabilized by direct or indirect interactions with ligands, leading to changes in their melting-curve profiles, which are obtained by measuring their apparent solubility.

**Figure 2:**
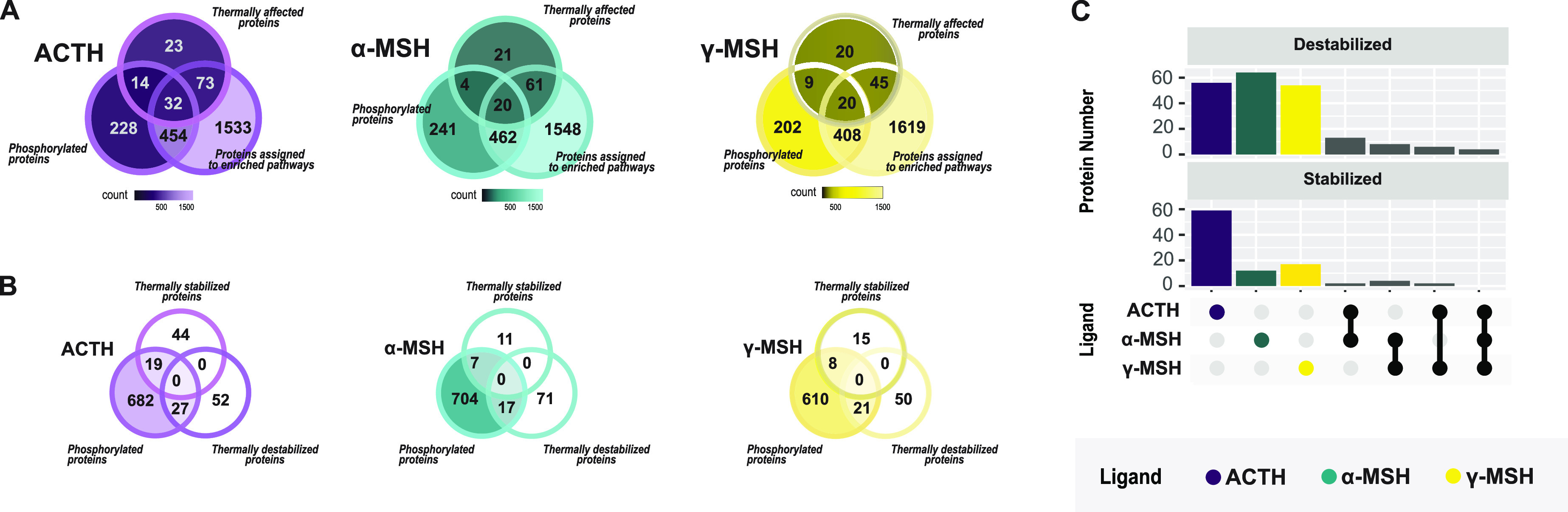
Overview of identified proteins and thermally stabilized or destabilized proteins. (A) Venn diagrams showing the numbers of proteins exhibiting altered melting points, associations with enriched pathways, and phosphorylation in MC3R-expressing HEK293 cells incubated with ACTH, α-MSH, and γ-MSH. (B) Venn diagrams showing the numbers of stabilized, destabilized, and phosphorylated proteins after incubation with ACTH, α-MSH, and γ-MSH. (C) Upset plot representing individual numbers of stabilized and destabilized proteins for each ligand and those common between various combinations of ligands.

**Figure 3:**
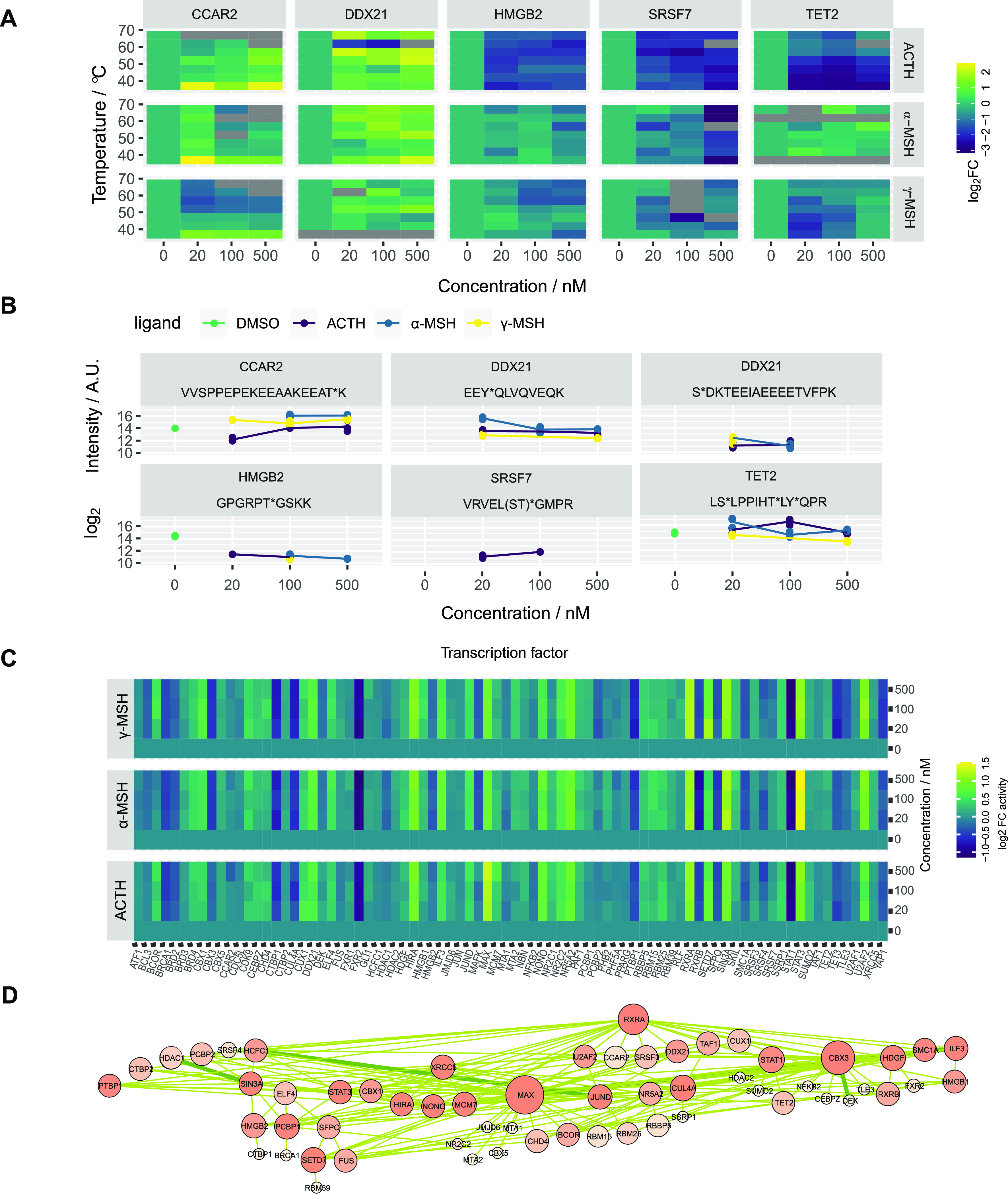
Characterization of transcription factors. (A) Heat map showing the relative abundance (compared to vehicle-only controls) of the transcription factors CCAR2, HMGB2, DDX21, SRSF7, and TET2 in MC3R-expressing HEK293 cells incubated with ACTH, α-MSH, and γ-MSH at different ligand concentrations and temperatures. (B) Phosphorylation of tryptic peptides derived from the thermally stabilized and destabilized transcription factors shown in panel A whose activity was inferred to change following stimulation with ACTH, α-MSH, or γ-MSH. Phosphorylation sites are indicated by asterisks next to the modified amino acid (shown in parentheses when the exact amino acid is unknown). (C) Transcription factor activities and relational networks inferred from differential expression data using BITFAM.^23^ The heatmap shows fold changes in transcription factor activities (relative to vehicle-only treatments) in MC3R-expressing HEK293 cells incubated with ACTH, α-MSH, or γ-MSH. (D) Network showing the interconnectivity of the transcription factors identified within our experimental LC–MS data set.

We found that in total 298 proteins were thermally stabilized or destabilized upon stimulation of MC3R across its endogenous agonists. Of these proteins, only 4 were affected by all three ligands. Another 36 proteins were affected by two ligands (ACTH and α-MSH, ACTH and γ-MSH, or α-MSH and γ-MSH), leaving 258 proteins whose thermal stability was affected by only one of the POMC neuropeptides (Figure 2C). Overall, treatment with ACTH, α-MSH, and γ-MSH altered the thermal stability of 142, 106, and 94 proteins, respectively (Figure 2A). Furthermore, PTMs play central roles in functional proteomics because they influence the activity, localization, and synthesis of proteins. We therefore used peptide sequencing software to search our LC–MS data set for phosphorylation as a variable modification, revealing that in total 104 of the thermally stabilized or destabilized proteins were also phosphorylated across the three ligands. However, there was no detectable tendency toward overrepresentation of phosphorylation among the thermally stabilized or destabilized proteins: 19 of the proteins thermally stabilized and 27 of the proteins thermally destabilized by ACTH were also phosphorylated, while the corresponding numbers were 7 and 17 for α-MSH and 8 and 21 for γ-MSH, respectively (Figure 2B). These numbers correspond to roughly a third of the proteins thermally affected by ACTH and γ-MSH and a quarter of those thermally affected by α-MSH. Moreover, of the 104 proteins exhibiting both altered thermal stability and phosphorylation, 72 were phosphorylated in samples treated with an MC3R agonist but not in vehicle-only controls, suggesting their phosphorylation was induced by the agonist’s interaction with MC3R. The remaining 32 proteins were also phosphorylated in the control samples (Supporting Information file Phosphopeptides). Figure 3A,B shows the 2D-TPP results and phosphorylation data for five transcription factors that are discussed in more detail below.

### Gene Set Enrichment Analysis and Transcription Factor Activity Inference and Validation

The majority of the systemic effects resulting from interactions between a receptor and its ligands are due to differential expression. We identified 1598, 1557, and 1474 proteins exhibiting increased expression after treatment with ACTH, α-MSH, and γ-MSH, respectively, as well as 610, 682, and 835 proteins exhibiting reduced expression, respectively. These changes in expression can be attributed to the ligand effects on transcription, translation, and protein degradation. In connection to differential expression, transcription factors are the main initiators and regulators of gene transcription and thus connect cell signaling to gene expression. Consequently, their activation and regulation are governed by multiple pathways and the formation of transcription factor complexes.^32^ Our analysis revealed a network of transcription factors affected by the applied treatments and generated a connectivity map that sheds light on the relationships between these transcription factors and their regulatory targets (Figure 3D). Because transcription factor modulation is regulated by PTMs such as phosphorylation, their activity is not determined solely by their expression.^33,34^

Data analysis with the Reactome search engine and gene set enrichment analysis showed which biological pathways were primarily affected by the ligand treatment. Among the enriched pathways with a high number of thermally affected proteins are signaling pathways involved in signal transduction and protein turn over (Table 1). This was expected as ligand-mediated receptor activation initiates various signaling cascades based on other protein–ligand interactions. Further, we detected effects on the immune system, where MC3R plays a role in the modulation of immune and anti-inflammatory responses. Proteins involved in the enriched pathways were related to the results from the TPP experiment as well as from the phosphorylation and transcription factor analysis (Figure 2A). For the proteins connected to the immune system, this led to the identification of five transcription factors: cell cycle and apoptosregulator protein 2 (CCAR2), nucleolar RNA helicase 2 (DDX21), high mobility group protein B2 (HMGB2), serine/arginine-rich splicing factor 7 (SRSF7), and methylcytosine dioxygenase TET2 (TET2). These transcription factors play crucial roles in regulating immune responses, and their activation or inhibition can have significant effects on immune-related pathways. Figure 3B shows the relative abundances of tryptic phosphorylated peptides derived from these proteins. An observed increase in phosphorylation upon the ligand treatment can be the consequence of an increased expression of the protein, an increased number of phosphorylations, or a combination of both. The trends in the inferred transcription factor activities shown in Figure 3C agree well with the trends in the relative abundances of the corresponding phosphorylated tryptic peptides shown in Figure 3B. It is particularly notable that both the inferred activities of DDX21, SRSD7, and TET2 and the relative abundances of phosphorylated peptides derived from these proteins increased across the studied range of ligand concentrations. The locations of the phosphorylation sites given in Figure 3B are unambiguous for all peptides except for VRVEL(ST)*GMPR from SRSF7 where the fragmentation data cannot rule out whether the phosphorylation is located on the serine or tyrosine residue (Supporting Information file STY-peptides). Moreover, the abundance of the phosphorylated form of the DDX21-derived peptide EEY*QLVQVEQK in the ligand treatments was higher than in the vehicle-only controls. This is also the only DDX21 derived phosphopeptide in which the only possible phosphorylation site is a tyrosine residue, which is notable because tyrosine phosphorylation is considered to regulate transcription factor activity whereas serine or threonine phosphorylation may have other modulatory effects. We therefore consider this finding to be strongly consistent with the inferred increase in DDX21 activity. In contrast, both the inferred transcription factor activity of HMGB2 and the relative abundance of its phosphopeptides decreased as the ligand concentration increased, while the inferred activity of CCAR2 decreased but the relative abundance of its phosphopeptides remained stable. Phosphorylation plots for the 104 proteins exhibiting altered thermal stability are presented together with the associated phosphopeptides in the Supporting Information file Phosphopeptides.

**Table 1:** Numbers of Thermally Stabilized or Destabilized Proteins Associated with Enriched Pathways, Where the Pathways Are Grouped under Their Top Levels within the Reactome

Top level	ACTH	α-MSH	γ-MSH
Autophagy	5	2	3
Cell–cell communication	1	2	1
Cell cycle	9	4	14
Cellular responses to stimuli	11	4	8
Chromatin organization	1	1	–
Developmental biology	14	5	4
DNA repair	3	–	2
DNA replication	–	–	1
Gene expression (transcription)	8	5	11
Hemostasis	5	2	1
Immune system	12	5	6
Metabolism	22	9	9
Metabolism of proteins	33	13	16
Metabolism of RNA	15	11	7
Muscle contraction	2	3	1
Neuronal System	4	3	3
Organelle biogenesis and maintenance	4	1	4
Programmed cell death	1	–	1
Protein localization	2	–	–
Sensory perception	1	–	–
Signal transduction	21	21	15
Transport of small molecules	2	3	1
Vesicle-mediated transport	10	10	5
No association with enriched pathway	133	98	90

The increase in the inferred activity of other transcription factors, including STAT3, MAX, and NONO, as well as the inhibition of STAT1 and FXR2, highlights the intricate interplay between these key players after ligand treatment. The significance of phosphorylation and thermal stabilization of transcription factors like CCAR2, DDX21, HMGB2, SRSF7, and TET2 becomes evident when considering the pathways affected by the POMC ligand treatment. CCAR2, involved in T-cell immune responses, and DDX21, a contributor to the JUN signaling cascade, shed light on the complex regulation of immune functions. The influence of HMGB2, a pro-inflammatory factor, further emphasizes the multifaceted nature of the signaling pathways affected by POMC ligands. Additionally, the involvement of SRSF7 in immune responses and apoptosis suggests its role in orchestrating immune system dynamics. The thermal destabilization of TET2, which modulates IL-6 activity during inflammation, provides further insight into the intricate molecular mechanisms at play. Notably, these findings not only deepen our understanding of the immune response but also highlight the effectiveness of our novel data pipeline in uncovering ligand-specific mechanistic differences within MC3R signaling cascades without additional experimental work.

## Conclusion

We used label-free quantitative TPP to characterize proteomic effects resulting from the binding of ACTH, α-MSH, and γ-MSH to MC3R. The multidimensional data gathered using this technique allowed us to perform a differential expression analysis that linked proteins exhibiting altered thermal stability to the enrichment of specific biological pathways. This was possible because our new workflow provides a wealth of quantitative data and permits gene set enrichment analysis using GO and Reactome annotation terms. Unlike previous TPP protocols in which protein classification is based exclusively on direct GO annotations, our workflow incorporates a statistical analysis to identify affected biochemical pathways and infer transcription factor from the data set. The inclusion of gene set enrichment data allowed us to clarify the mechanistic roles of proteins exhibiting altered thermal stability and their functions within differentially regulated biochemical pathways. As a result, although ACTH, α-MSH, and γ-MSH had similar overall effects on signaling pathways, ligand-induced changes observed in CCAR2, DDX21, HMGB2, SRSF7, and TET2 shed light on their roles in immune responses and their modulation by POMC ligands. These findings highlight the applicability and efficiency of our workflow as they were obtained without the need for additional experiments. The results obtained will provide valuable guidance for follow-up experiments on human MC3R-expressing immune cells, such as macrophages.

The workflow presented here begins with a TPP assay using label-free LC–MS-based proteomics. The experimental data from this assay are then analyzed using a multidimensional data processing workflow. This methodology could easily be used to address other research questions concerning the relationships between receptors and their endogenous or synthetic ligands. Importantly, it also allows researchers to extract multiple layers of information to obtain deep mechanistic insights into the molecular consequences of receptor stimulation by specific drugs.

## Data Availability

The mass spectrometry proteomics data have been deposited to the ProteomeXchange Consortium via the PRIDE partner repository and are accessible via DOI 10.6019/PXD039945.

## Supporting Information Available

Figures showing cAMP assay data for validation of MC3R-expression, robustness of the LC–MS system for protein identification, and TPP inference algorithms (PDF)
Phosphopeptides: Relative abundance of phosphorylated peptides belonging to proteins exhibiting altered thermal stability at 37 °C (PDF)
TPP_ACTH: ISOQuant report file for analysis of relative protein abundance (XLSX)
TPP_alpha: ISOQuant report file for analysis of relative protein abundance (XLSX)
TPP_gamma: ISOQuant report file for analysis of relative protein abundance (XLSX)
List of identified proteins exhibiting altered thermal stability (XLSX)
Full Reactome pathway analysis for all drug and concentration conditions at 37 °C (XLSX)
STY-peptides: ISOQuant report file for the analysis of relative protein abundance under all drug and concentration conditions at 37 °C with phosphorylation used as a variable modification in the preceding PLGS search (XLSX)

## References

[ref1] Jafari R.; Almqvist H.; Axelsson H.; Ignatushchenko M.; Lundbäck T.; Nordlund P.; Molina D. M. The cellular thermal shift assay for evaluating drug target interactions in cells. Nat. Protoc. 2014, 9, 2100–2122. 10.1038/nprot.2014.138. 25101824

[ref2] Franken H.; Mathieson T.; Childs D.; Sweetman G.; Werner T.; Tögel I.; Doce C.; Gade S.; Bantscheff M.; Drewes G.; Reinhard F. B. M.; Huber W.; Savitski M. M. Thermal proteome profiling for unbiased identification of direct and indirect drug targets using multiplexed quantitative mass spectrometry. Nat. Protoc. 2015, 10, 1567–1593. 10.1038/nprot.2015.101. 26379230

[ref3] Becher I.; Werner T.; Doce C.; Zaal E. A.; Tögel I.; Khan C. A.; Rueger A.; Muelbaier M.; Salzer E.; Berkers C. R.; et al. Thermal profiling reveals phenylalanine hydroxylase as an off-target of panobinostat. Nat. Chem. Biol. 2016, 12, 908–910. 10.1038/nchembio.2185. 27669419

[ref4] Sridharan S.; Kurzawa N.; Werner T.; Günthner I.; Helm D.; Huber W.; Bantscheff M.; Savitski M. M. Proteome-wide solubility and thermal stability profiling reveals distinct regulatory roles for ATP. Nat. Commun. 2019, 10, 1155 10.1038/s41467-019-09107-y. 30858367 PMC6411743

[ref5] Mateus A.; Kurzawa N.; Becher I.; Sridharan S.; Helm D.; Stein F.; Typas A.; Savitski M. M. Thermal proteome profiling for interrogating protein interactions. Mol. Syst. Biol. 2020, 16, e9232 10.15252/msb.20199232. 32133759 PMC7057112

[ref6] Mateus A.; Määttä T. A.; Savitski M. M. Thermal proteome profiling: unbiased assessment of protein state through heat-induced stability changes. Proteome Sci. 2016, 15, 13 10.1186/s12953-017-0122-4. 28652855 PMC5482948

[ref7] Carnero Corrales M. A.; Zinken S.; Konstantinidis G.; Rafehi M.; Abdelrahman A.; Wu Y.-W.; Janning P.; Müller C. E.; Laraia L.; Waldmann H. Thermal proteome profiling identifies the membrane-bound purinergic receptor P2 × 4 as a target of the autophagy inhibitor indophagolin. Cell Chem. Biol. 2021, 28, 1750–1757. 10.1016/j.chembiol.2021.02.017. 33725479

[ref8] Ruan C.; Wang Y.; Zhang X.; Lyu J.; Zhang N.; Ma Y.; Shi C.; Qu G.; Ye M. Matrix Thermal Shift Assay for Fast Construction of Multidimensional Ligand–Target Space. Anal. Chem. 2022, 94, 6482–6490. 10.1021/acs.analchem.1c04627. 35442643

[ref9] Ye Y.; Li K.; Ma Y.; Zhang X.; Li Y.; Yu T.; Wang Y.; Ye M. The Introduction of Detergents in Thermal Proteome Profiling Requires Lowering the Applied Temperatures for Efficient Target Protein Identification. Molecules 2023, 28, 4859 10.3390/molecules28124859. 37375414 PMC10301966

[ref10] Gholizadeh E.; Karbalaei R.; Khaleghian A.; Salimi M.; Gilany K.; Soliymani R.; Tanoli Z.; Rezadoost H.; Baumann M.; Jafari M.; et al. Identification of celecoxib-targeted proteins using label-free thermal proteome profiling on rat hippocampus. Mol. Pharmacol. 2021, 99, 308–318. 10.1124/molpharm.120.000210. 33632781

[ref11] Wang W.; Guo D.-Y.; Lin Y.-J.; Tao Y.-X. Melanocortin regulation of inflammation. Front. Endocrinol. 2019, 10, 683 10.3389/fendo.2019.00683. PMC6794349 31649620

[ref12] Marks D. L.; Hruby V.; Brookhart G.; Cone R. D. The regulation of food intake by selective stimulation of the type 3 melanocortin receptor (MC3R). Peptides 2006, 27, 259–264. 10.1016/j.peptides.2005.01.025. 16274853 PMC1679957

[ref13] Sweeney P.; Bedenbaugh M. N.; Maldonado J.; Pan P.; Fowler K.; Williams S. Y.; Gimenez L. E.; Ghamari-Langroudi M.; Downing G.; Gui Y.; et al. The melanocortin-3 receptor is a pharmacological target for the regulation of anorexia. Sci. Transl. Med. 2021, 13, eabd6434 10.1126/scitranslmed.abd6434. 33883274 PMC9022017

[ref14] Lam C. W.; Getting S. J. Melanocortin receptor type 3 as a potential target for anti-inflammatory therapy. Curr. Drug Targets: Inflammation Allergy 2004, 3, 311–315. 10.2174/1568010043343606. 15379600

[ref15] Lisak R. P.; Benjamins J. A. Melanocortins, melanocortin receptors and multiple sclerosis. Brain Sci. 2017, 7, 104 10.3390/brainsci7080104. 28805746 PMC5575624

[ref16] Maaser C.; Kannengiesser K.; Kucharzik T. Role of the melanocortin system in inflammation. Ann. N.Y. Acad. Sci. 2006, 1072, 123–134. 10.1196/annals.1326.016. 17057195

[ref17] Moscowitz A. E.; Asif H.; Lindenmaier L. B.; Calzadilla A.; Zhang C.; Mirsaeidi M. The importance of melanocortin receptors and their agonists in pulmonary disease. Front. Med. 2019, 6, 145 10.3389/fmed.2019.00145. PMC6610340 31316990

[ref18] Gantz I.; Fong T. M. The melanocortin system. Am. J. Physiol. Endocrinol. Metab. 2003, 284, E468–E474. 10.1152/ajpendo.00434.2002. 12556347

[ref19] Sandbaumhüter F. A.; Nezhyva M.; Eriksson O.; Engberg A.; Kreuger J.; Andrén P. E.; Jansson E. T. Well-Plate μFASP for Proteomic Analysis of Single Pancreatic Islets. J. Proteome Res. 2022, 21, 1167–1174. 10.1021/acs.jproteome.2c00047. 35293755 PMC8981318

[ref20] Distler U.; Kuharev J.; Navarro P.; Levin Y.; Schild H.; Tenzer S. Drift time-specific collision energies enable deep-coverage data-independent acquisition proteomics. Nat. Methods 2014, 11, 167–170. 10.1038/nmeth.2767. 24336358

[ref21] Distler U.; Kuharev J.; Navarro P.; Tenzer S. Label-free quantification in ion mobility–enhanced data-independent acquisition proteomics. Nat. Protoc. 2016, 11, 795–812. 10.1038/nprot.2016.042. 27010757

[ref22] Zhang B.; Chambers M. C.; Tabb D. L. Proteomic parsimony through bipartite graph analysis improves accuracy and transparency. J. Proteome Res. 2007, 6, 3549–3557. 10.1021/pr070230d. 17676885 PMC2810678

[ref23] Gao S.; Dai Y.; Rehman J. A Bayesian inference transcription factor activity model for the analysis of single-cell transcriptomes. Genome Res. 2021, 31, 1296–1311. 10.1101/gr.265595.120. 34193535 PMC8256867

[ref24] Mountjoy K. G. In The Melanocortin Receptors; Springer, 2000; pp 209–235.

[ref25] Lee E. J.; Lee S.-H.; Jung J.-W.; Lee W.; Kim B. J.; Park K. W.; Lim S.-K.; Yoon C.-J.; Baik J.-H. Differential regulation of cAMP-mediated gene transcription and ligand selectivity by MC3R and MC4R melanocortin receptors. Eur. J. Biochem. 2001, 268, 582–591. 10.1046/j.1432-1327.2001.01900.x. 11168397

[ref26] Durek T.; Cromm P. M.; White A. M.; Schroeder C. I.; Kaas Q.; Weidmann J.; Ahmad Fuaad A.; Cheneval O.; Harvey P. J.; Daly N. L.; et al. Development of novel melanocortin receptor agonists based on the cyclic peptide framework of sunflower trypsin inhibitor-1. J. Med. Chem. 2018, 61, 3674–3684. 10.1021/acs.jmedchem.8b00170. 29605997 PMC5999400

[ref27] Distler U.; Sielaff M.; Tenzer S. In Quantitative Methods in Proteomics, Marcus K., Eisenacher M., Sitek B., Eds.; Springer US: New York, NY, 2021; pp 327–339. 10.1007/978-1-0716-1024-4_23 33950501

[ref28] Dayon L.; Affolter M. Progress and pitfalls of using isobaric mass tags for proteome profiling. Expert Rev. Proteomics 2020, 17, 149–161. 10.1080/14789450.2020.1731309. 32067523

[ref29] Collins B. C.; Gillet L. C.; Rosenberger G.; Röst H. L.; Vichalkovski A.; Gstaiger M.; Aebersold R. Quantifying protein interaction dynamics by SWATH mass spectrometry: application to the 14–3-3 system. Nat. Methods 2013, 10, 1246–1253. 10.1038/nmeth.2703. 24162925

[ref30] O’Connell J. D.; Paulo J. A.; O’Brien J. J.; Gygi S. P. Proteome-wide evaluation of two common protein quantification methods. J. Proteome Res. 2018, 17, 1934–1942. 10.1021/acs.jproteome.8b00016. 29635916 PMC5984592

[ref31] Pappireddi N.; Martin L.; Wühr M. A review on quantitative multiplexed proteomics. ChemBioChem. 2019, 20, 1210–1224. 10.1002/cbic.201800650. 30609196 PMC6520187

[ref32] Mitsis T.; Efthimiadou A.; Bacopoulou F.; Vlachakis D.; Chrousos G. P.; Eliopoulos E. Transcription factors and evolution: an integral part of gene expression. World Acad. Sci. 2020, 2, 3–8. 10.3892/wasj.2020.32.

[ref33] Weidemüller P.; Kholmatov M.; Petsalaki E.; Zaugg J. B. Transcription factors: Bridge between cell signaling and gene regulation. Proteomics 2021, 21, 2000034 10.1002/pmic.202000034. 34314098

[ref34] De Bastiani M. A.; Pfaffenseller B.; Klamt F. Master regulators connectivity map: a transcription factors-centered approach to drug repositioning. Front. Pharmacol. 2018, 9, 697 10.3389/fphar.2018.00697. 30034338 PMC6043797

