# Supplementary material for: Label-Free Quantitative Thermal Proteome Profiling Reveals Target Transcription Factors with Activities Modulated by MC3R Signaling
Source: Anal Chem. 2023 Oct 7;95(41):15400–8. doi: 10.1021/acs.analchem.3c03643 (PMC10585664; doi:10.1021/acs.analchem.3c03643)
Supplement: Supplementary file 1 — ac3c03643_si_007.pdf [file ac3c03643_si_007.pdf]

Supporting information: Label-free quantitative thermal proteome profiling reveals target transcription factors with activities modulated by MC3R signaling

Friederike A. Sandbaumhüter[1,§], Mariya Nezhyva[1,§], Per E. Andrén[1,2], and Erik T. Jansson[1,*]

[1]Department of Pharmaceutical Biosciences, Uppsala University, 751 24 Uppsala, Sweden
[2] Science for Life Laboratory, Spatial Mass Spectrometry, Uppsala University, 751 24 Uppsala, Sweden
[§]Contributed equally to this work
[*]Corresponding author, erik.jansson@uu.se

List of Tables

S1 PLGS protein identification processing parameters S-2
S2 PLGS protein identification workflow parameters S-2

List of Figures

S1 cAMP-assay for evaluation of MC3R-expression. S-3
S2 Assessment of protein identification robustness. S-3
S3 HeLa RSD histogram. S-4
S4 TPP inference algorithm. S-4
S5 Summary of identified proteins S-5
S6 Summary of identified phosphopeptides S-5

Table S1: PLGS protein identification processing parameters

Parameter	Value
Instrument Mode	Electrospray-Shotgun
Peak Width	Automatic
MS Resolution	Automatic
Lockmass Charge 1	
Lockmass Charge 2	785.8426
Lockmass Tolerance	0.25
Low Energy Threshold	150.0
High Energy Threshold	50.0
Starting RT	
Ending RT	
Bin Intensity Threshold	0

Table S2: PLGS protein identification workflow parameters

Category	Parameter	Value
Databank Search	Search Engine Type	PLGS
	Search Type	Electrospray-Shotgun
	Fasta Format	Uniprot
	Precursor MHP Window PPM	-1
	Product MHP Window PPM	-1
	Num Peptide for Protein Minimum	2
	False Positive Rate	1
Digests	Missed Cleavages	1
	Amino Acid Sequence Digestor	Trypsin
	Cleaves At	K, R (Excludes P at N-Term)
Modifications	Carbamidomethyl+C	Fixed, Delta Mass 57.0215
	Acetyl+K	Variable, Delta Mass 42.0106
	Amidation+C-TERM	Variable, Delta Mass -0.9840
	Deamidation+N	Variable, Delta Mass 0.9840
	Deamidation+Q	Variable, Delta Mass 0.9840
	Oxidation+M	Variable, Delta Mass 15.9949
	**Phosphoryl+STY**	**Variable, Delta Mass 79.9663**

*Phosphoryl-STY was added in search for phosphoryated proteins.

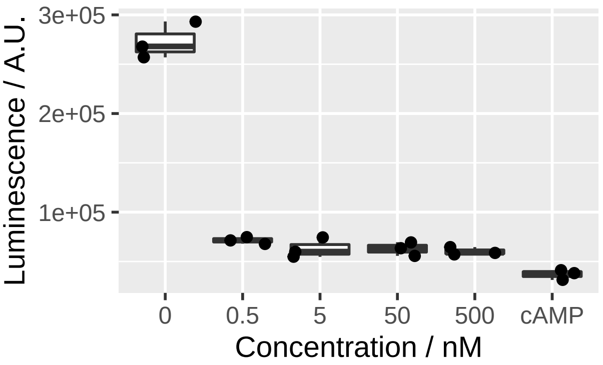

Figure S1: Evaluation of MC3R expression in stably transfected HEK293 cells. cAMP levels resulting from stimulus with various concentrations of γ-MSH are shown. Data points labeled cAMP show the results of positive control experiments where free cAMP was added to the sample. The luminescence is inversely proportional to the amount of free cAMP; higher amounts of free cAMP led to reduced luminescence and indicate activation of a GPCR.

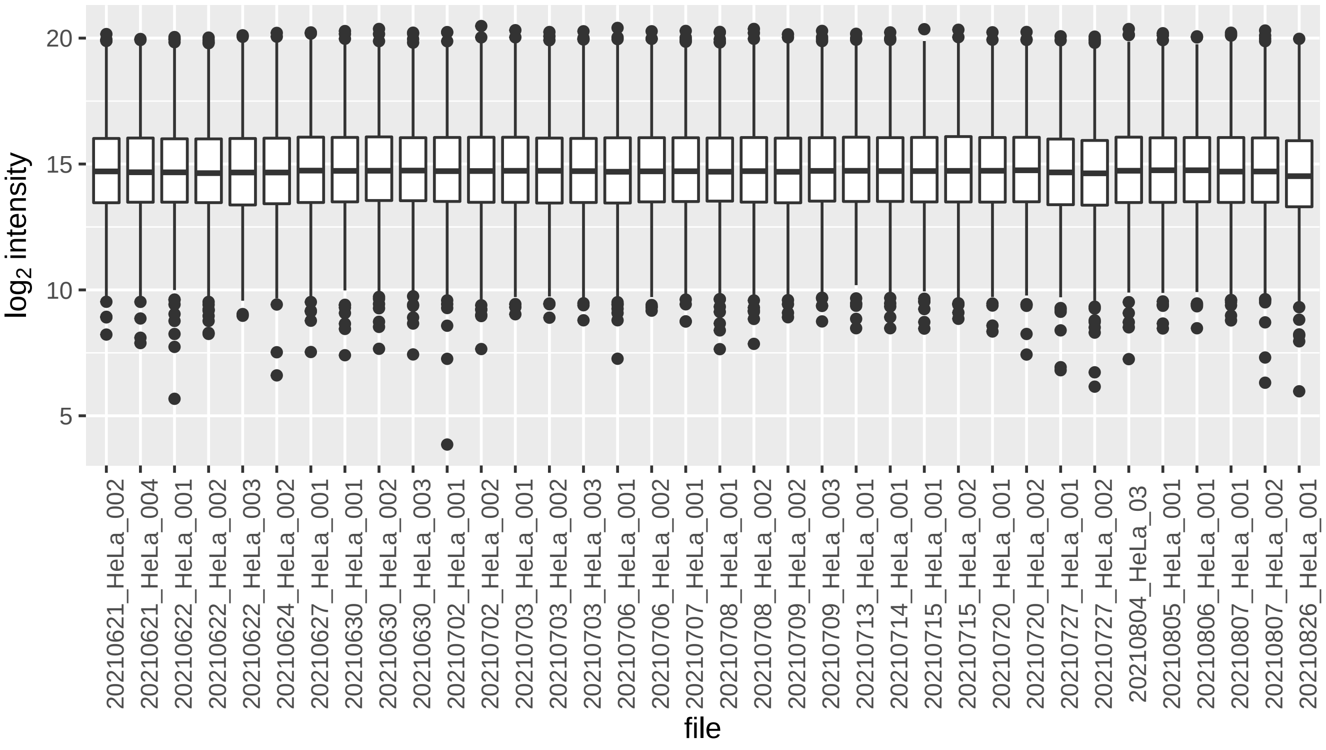

Figure S2: Box plots of protein intensities from LC–MS runs using HeLa standard digests over the course of the label-free quantitation experiment for thermal proteome profiling. Injections of HeLa digests were interspersed between TPP runs to monitor system stability.

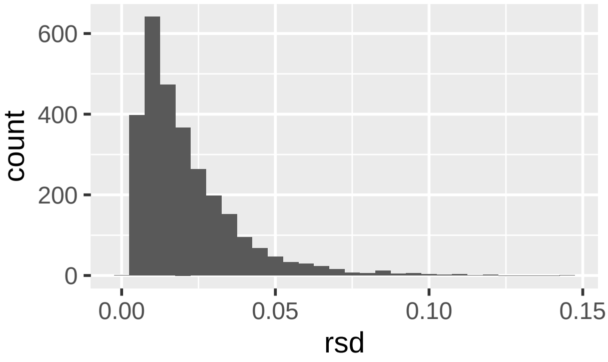

Figure S3: Histogram showing the distribution of protein RSD intensities from LC–MS runs using HeLa standards over the label-free quantitation experiment for thermal proteome profiling. Injections of HeLa digests were interspersed between TPP runs to monitor system stability.

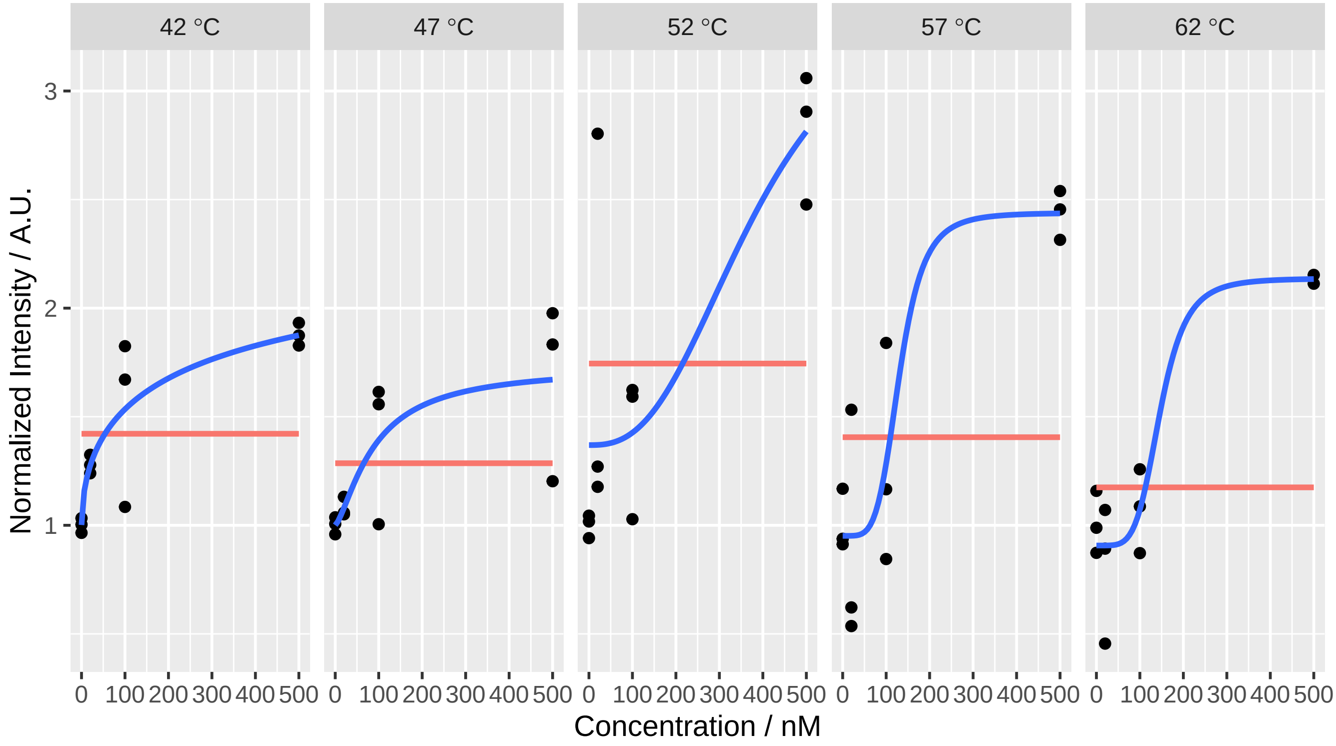

Figure S4: An illustration of the evaluation algorithm for identifying proteins exhibiting altered thermal stability after ligand stimulation. The figure shows relative abundance data for the protein CCAR2 following treatment with ACTH. The mean of the data for the vehicle-only condition (0 nM) is normalized to unity for each set of temperatures, which range from 42 to 62 °C. The alternate hypothesis H_1 is fitted to the data using a log-logistic function (blue) and is compared to the null hypothesis H_0, for which a constant function is fitted to the data (red). The residual error is then evaluated for both fits and used to determine the F-statistic.

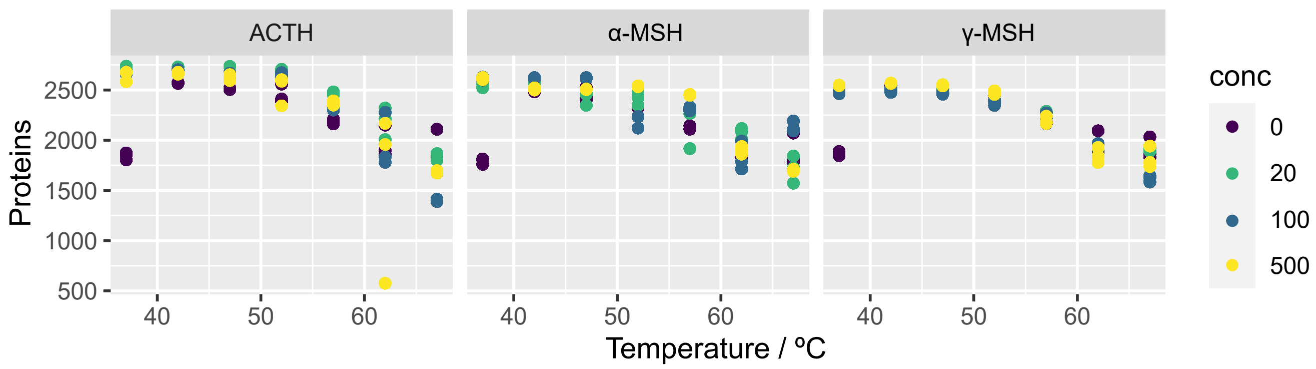

Figure S5: Summary of number of identified proteins from the various treatment conditions

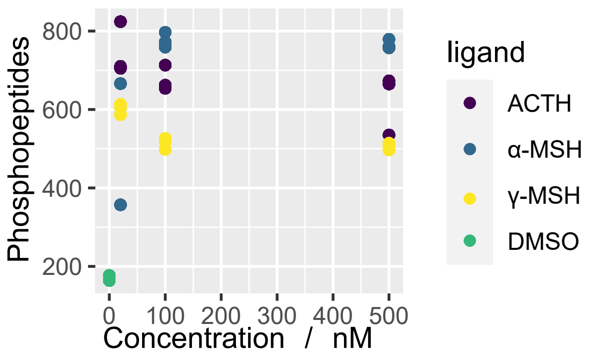

Figure S6: Summary of number of identified phosphopeptides with at least three observed fragments from the various treatment conditions
