# Supplementary material for: Label-Free Quantitative Thermal Proteome Profiling Reveals Target Transcription Factors with Activities Modulated by MC3R Signaling
Source: Anal Chem. 2023 Oct 7;95(41):15400–8. doi: 10.1021/acs.analchem.3c03643 (PMC10585664; doi:10.1021/acs.analchem.3c03643)
Supplement: Supplementary file 2 — ac3c03643_si_008.pdf [file ac3c03643_si_008.pdf]

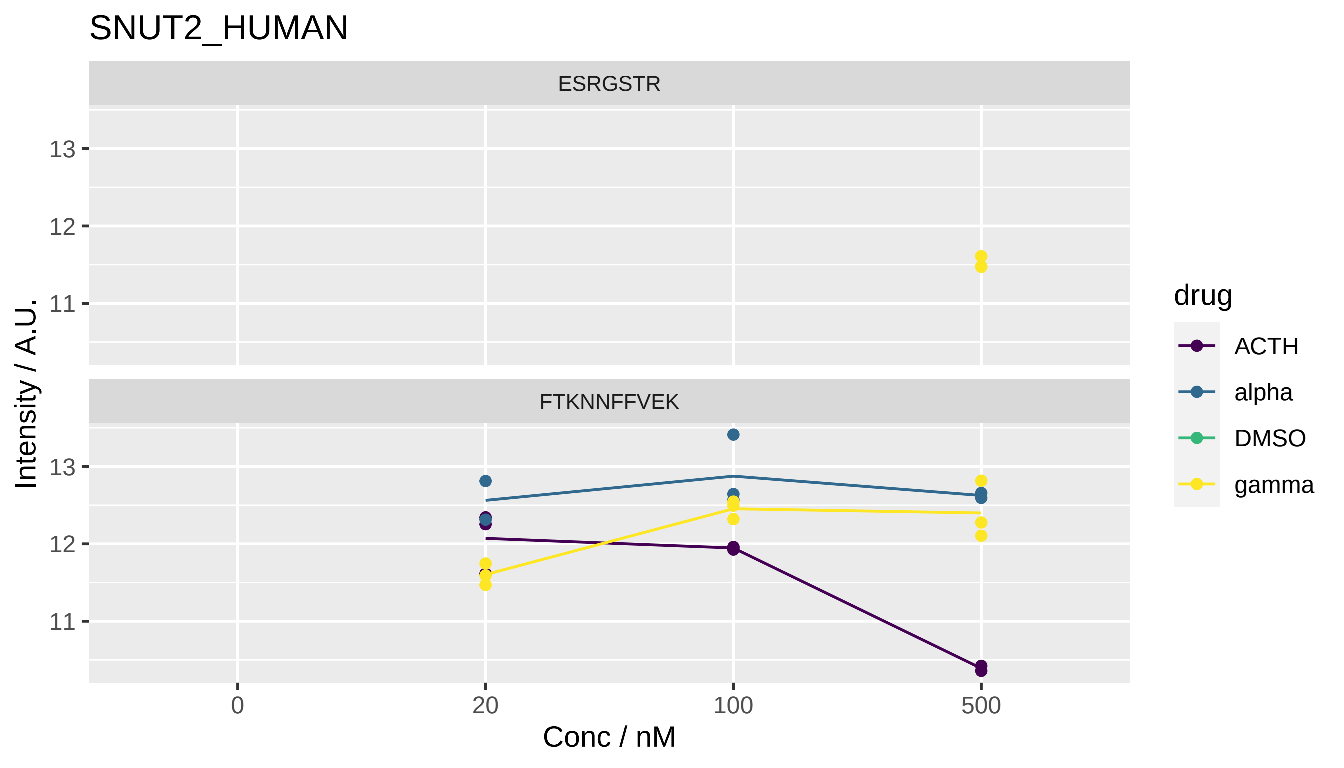
SNUT2_HUMAN
ESRGSTR
FTKNNFFVEK
Intensity / A.U.
13
12
11
13
12
11
0
20
100
500
Conc / nM
drug
ACTH
alpha
DMSO
gamma

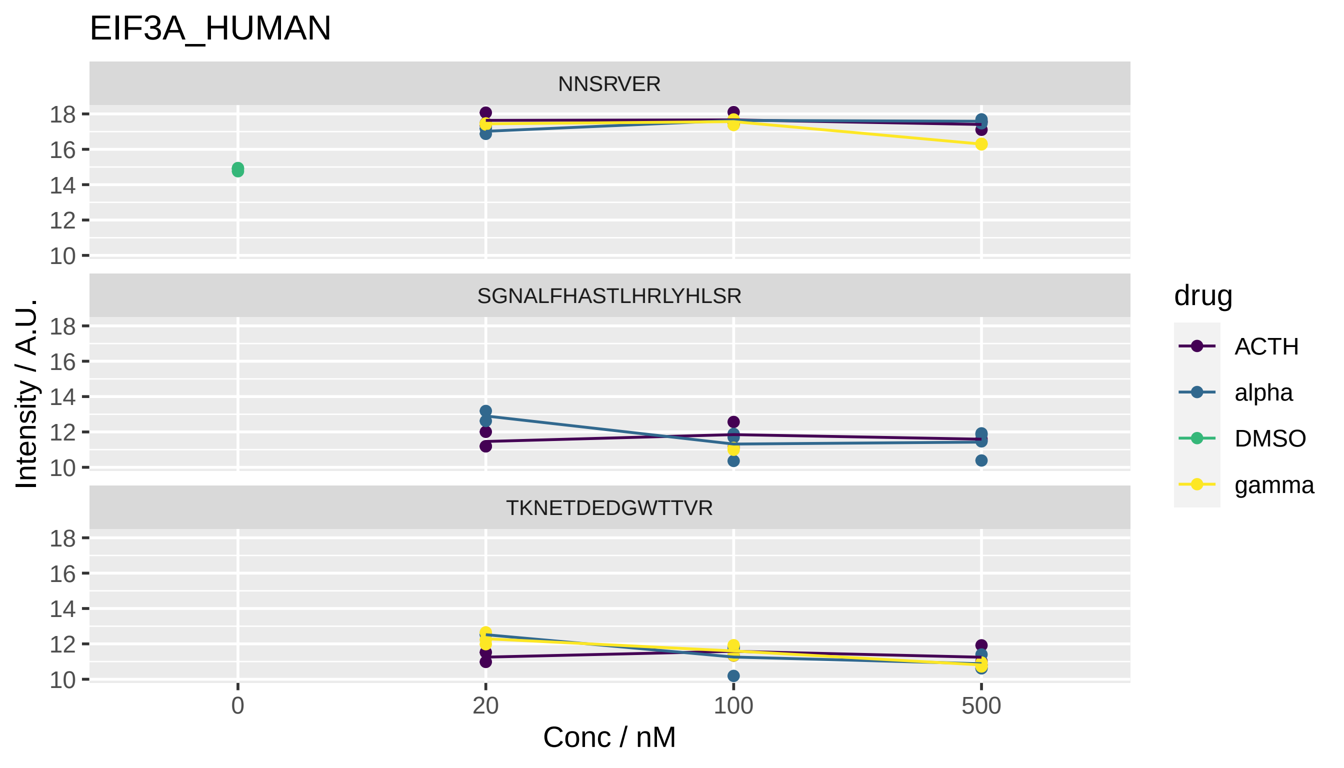

EIF3A_HUMAN
NNSRVER
SGNALFHASTLHRLYHLSR
TKNETDEDGWTTVR
Intensity / A.U.
Conc / nM
0
20
100
500
10
12
14
16
18
drug
ACTH
alpha
DMSO
gamma

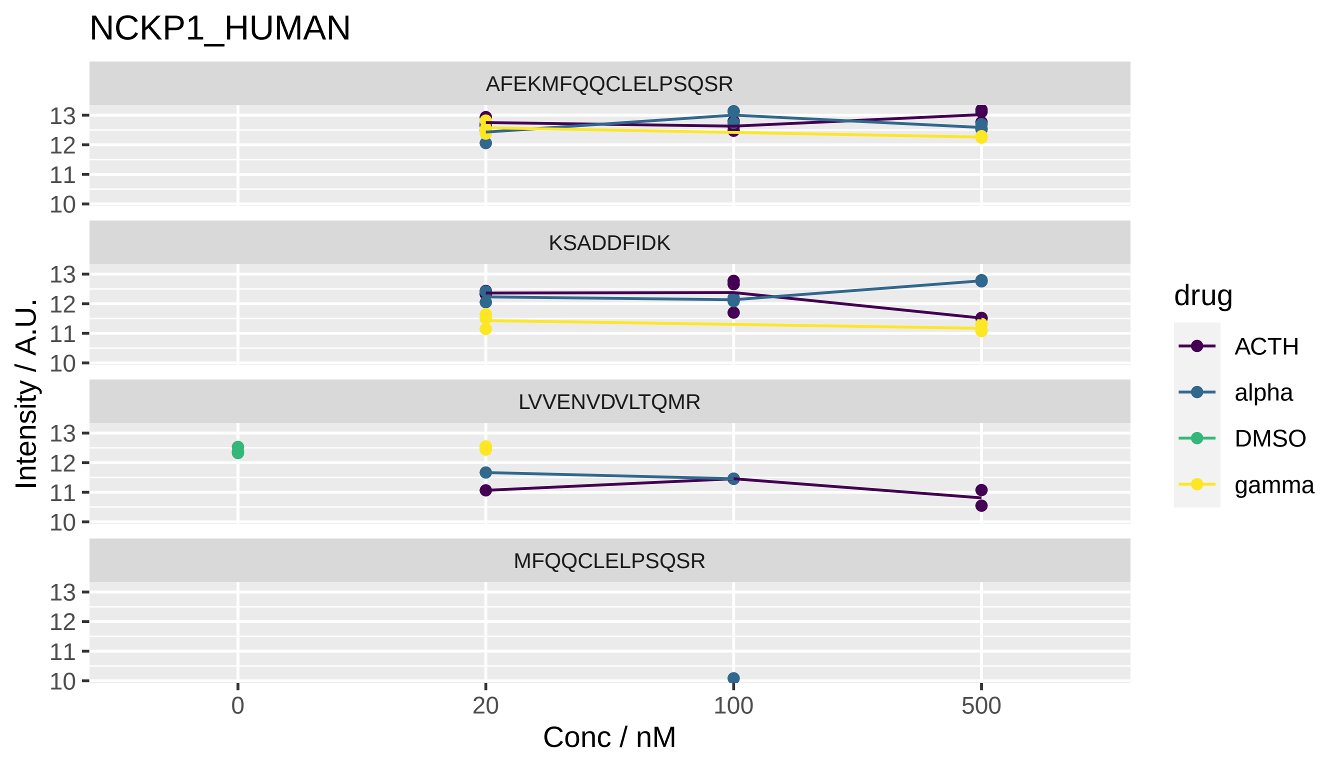

NCKP1_HUMAN
AFEKMFQQCLELPSQSR
KSADDFIDK
LVVENVDVLTQMR
MFQQCLELPSQSR
Intensity / A.U.
13
12
11
10
0
20
100
500
Conc / nM
drug
ACTH
alpha
DMSO
gamma

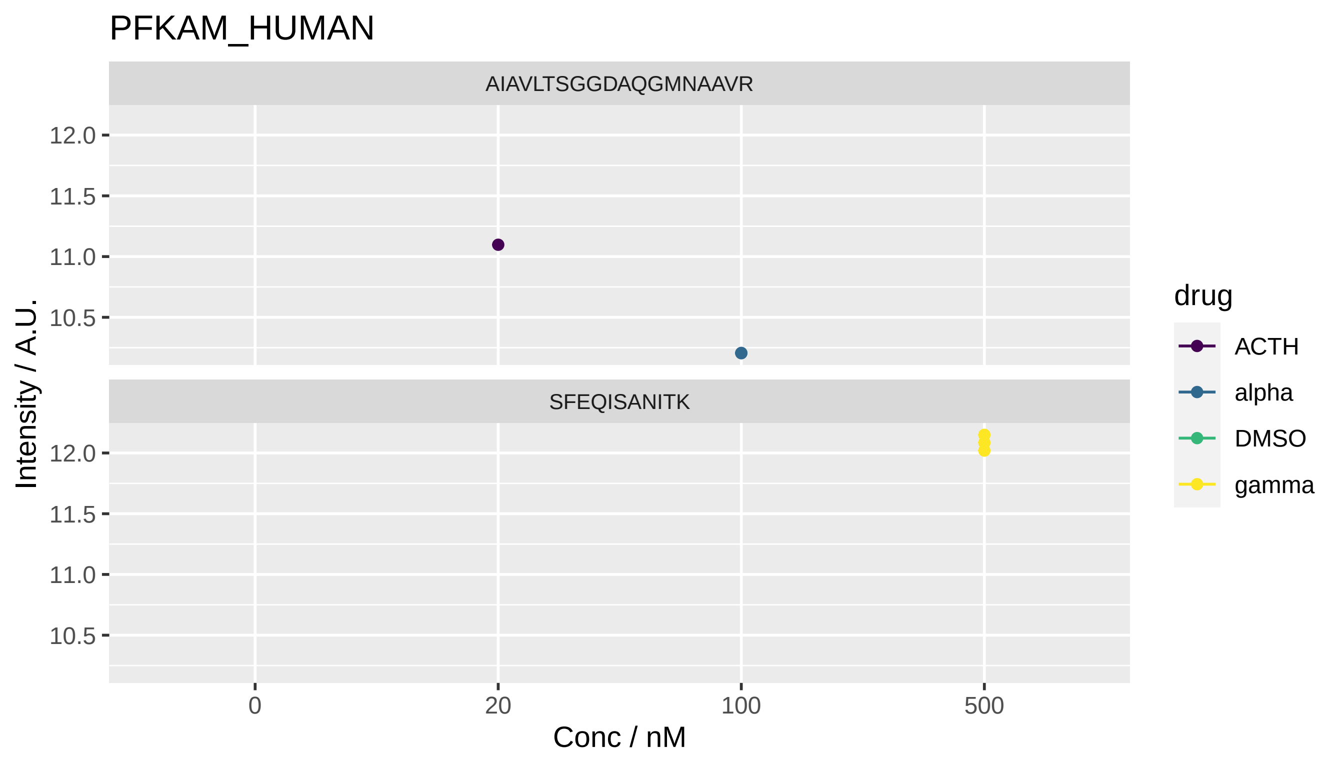
PFKAM_HUMAN
AIAVLTSGGDAQGMNAAVR
SFEQISANITK
12.0
11.5
11.0
10.5
Intensity / A.U.
0
20
100
500
Conc / nM
drug
ACTH
alpha
DMSO
gamma

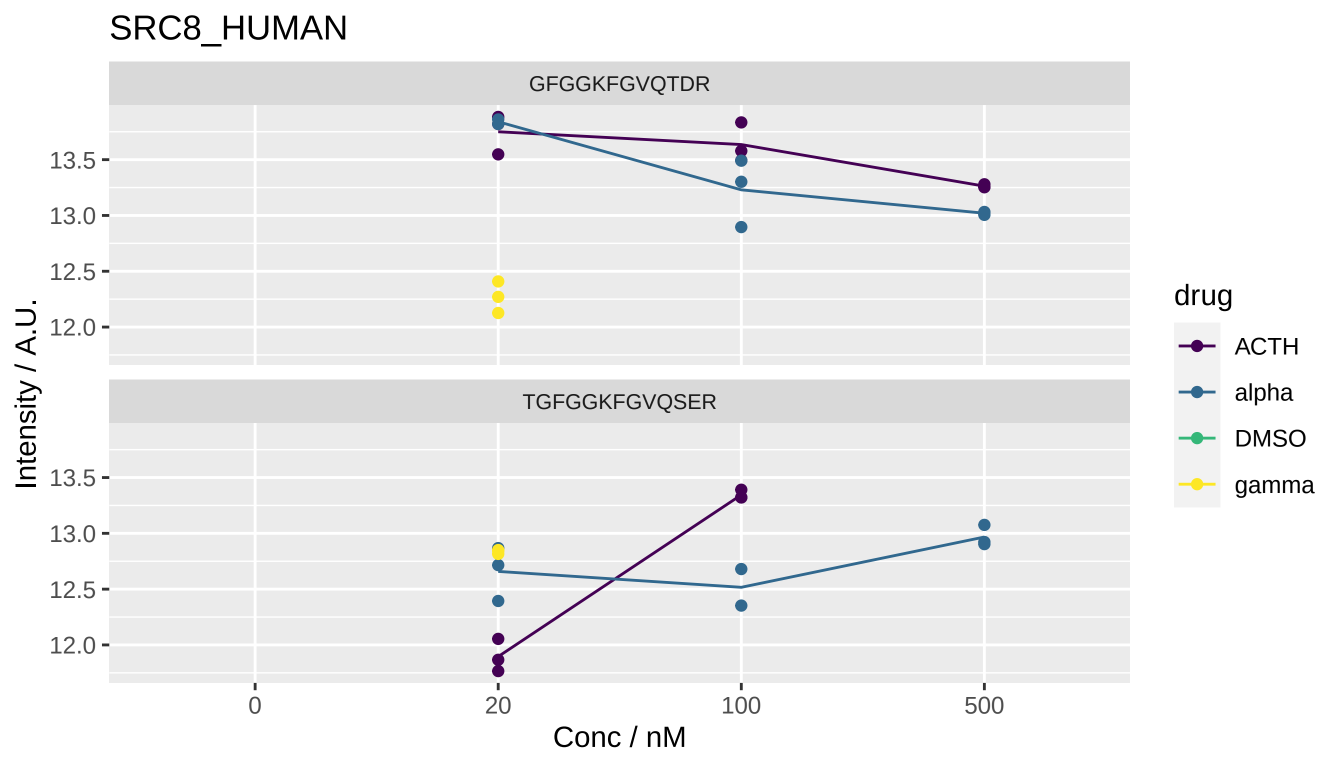
SRC8_HUMAN
GFGGKFGVQTDR
TGFGGKFGVQSER
Intensity / A.U.
13.5
13.0
12.5
12.0
Conc / nM
0
20
100
500
drug
ACTH
alpha
DMSO
gamma

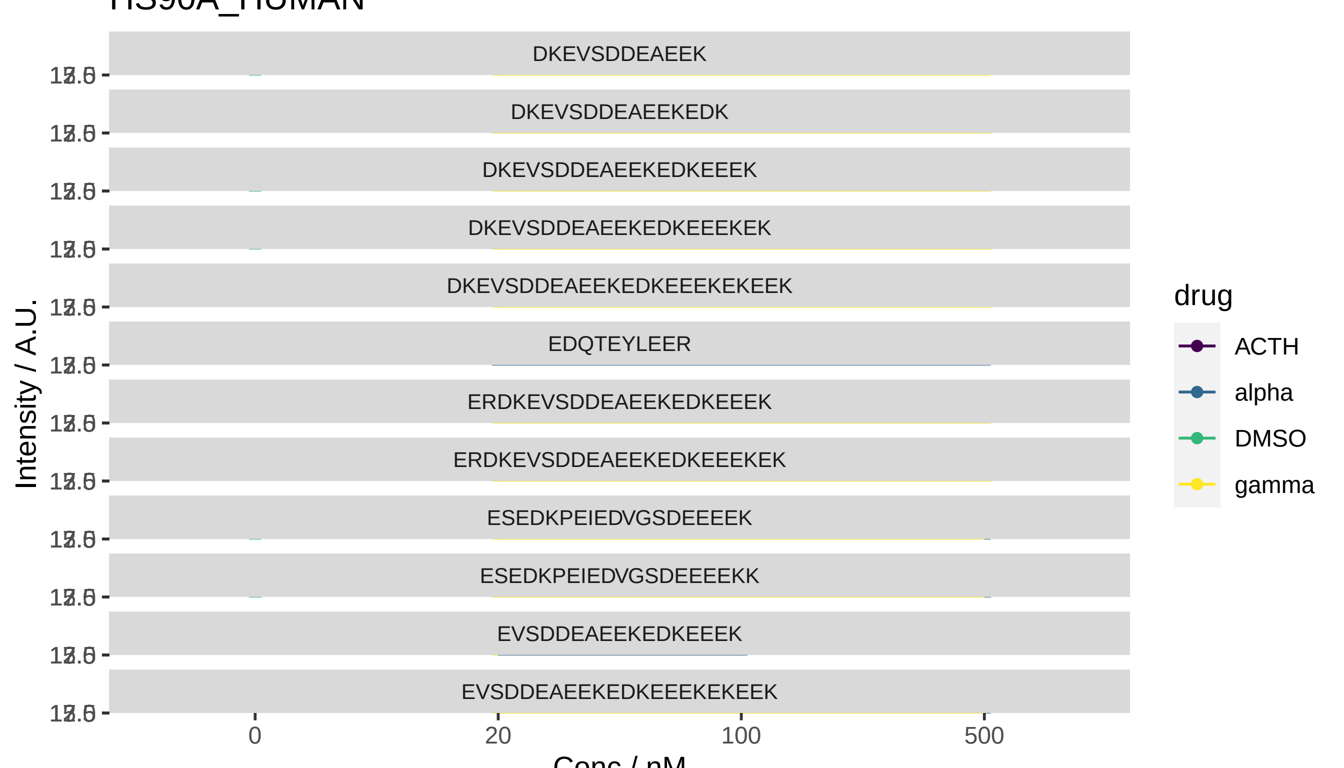

HS90A_HUMAN
DKEVSDDEAEEK
DKEVSDDEAEEKEDK
DKEVSDDEAEEKEDKEEEK
DKEVSDDEAEEKEDKEEEKEK
DKEVSDDEAEEKEDKEEEKEKEEK
EDQTEYLEER
ERDKEVSDDEAEEKEDKEEEK
ERDKEVSDDEAEEKEDKEEEKEK
ESEDKPEIEDVGSDEEEEK
ESEDKPEIEDVGSDEEEEKK
EVSDDEAEEKEDKEEEK
EVSDDEAEEKEDKEEEKEKEEK
Intensity / A.U.
0
20
100
500
Conc / nM
drug
ACTH
alpha
DMSO
gamma

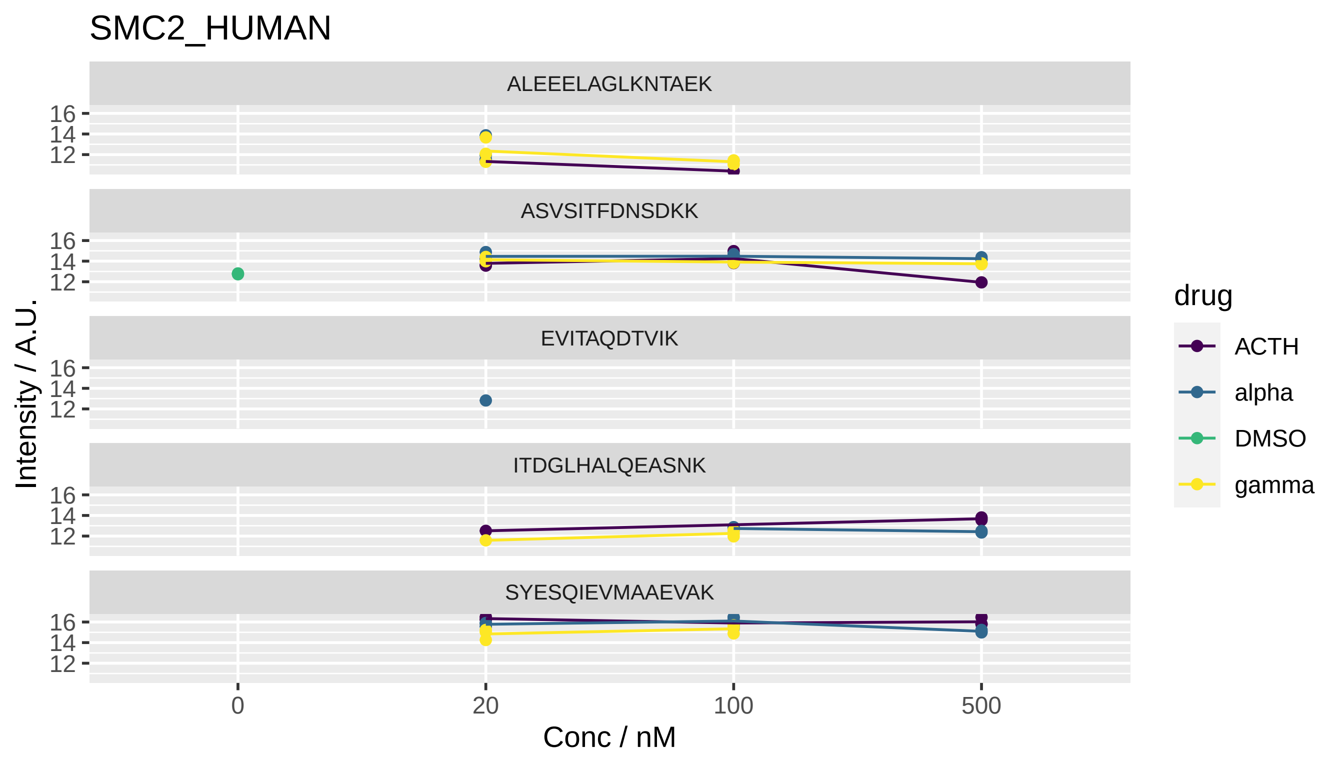

SMC2_HUMAN
ALEEELAGLKNTAEK
ASVSITFDNSDKK
EVITAQDTVIK
ITDGLHALQEASNK
SYESQIEVMAAEVAK
16
14
12
Intensity / A.U.
0
20
100
500
Conc / nM
drug
ACTH
alpha
DMSO
gamma

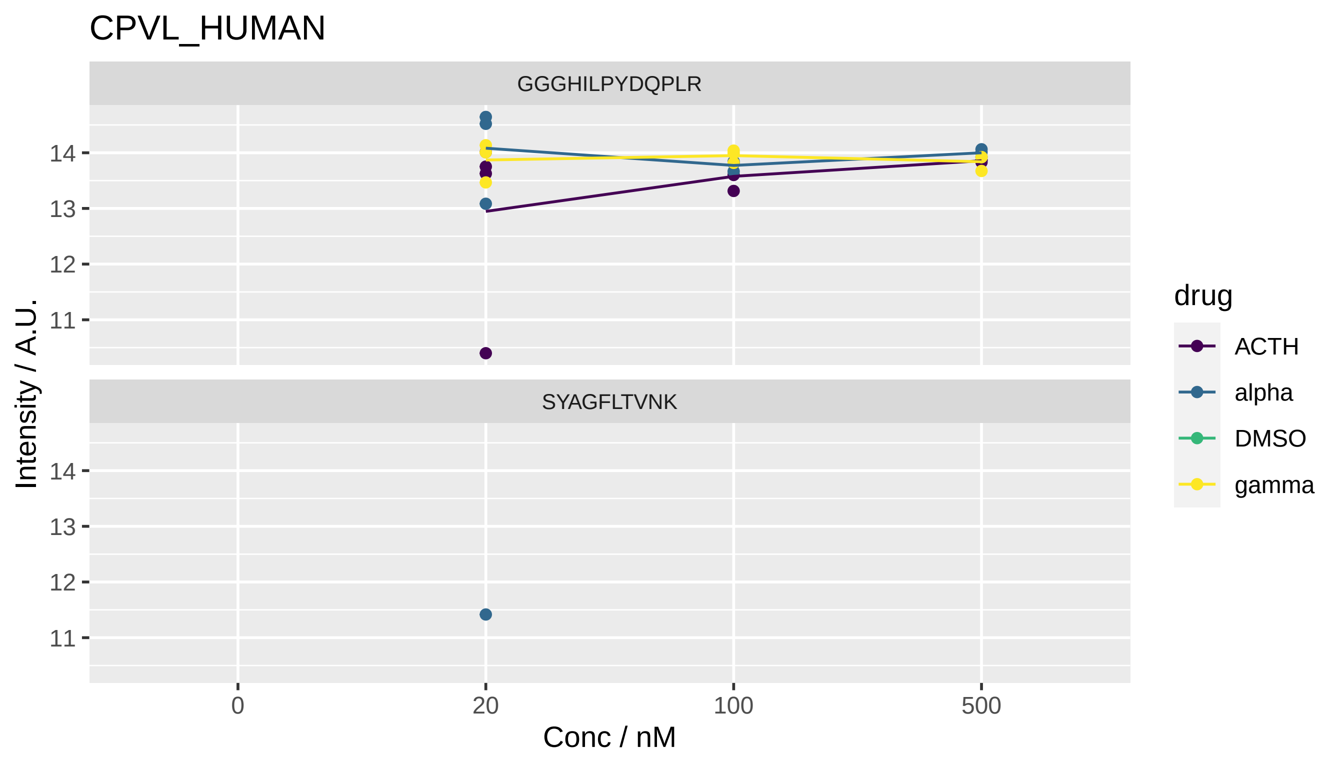
CPVL_HUMAN
GGGHILPYDQPLR
SYAGFLTVNK
Intensity / A.U.
14
13
12
11
0
20
100
500
Conc / nM
drug
ACTH
alpha
DMSO
gamma

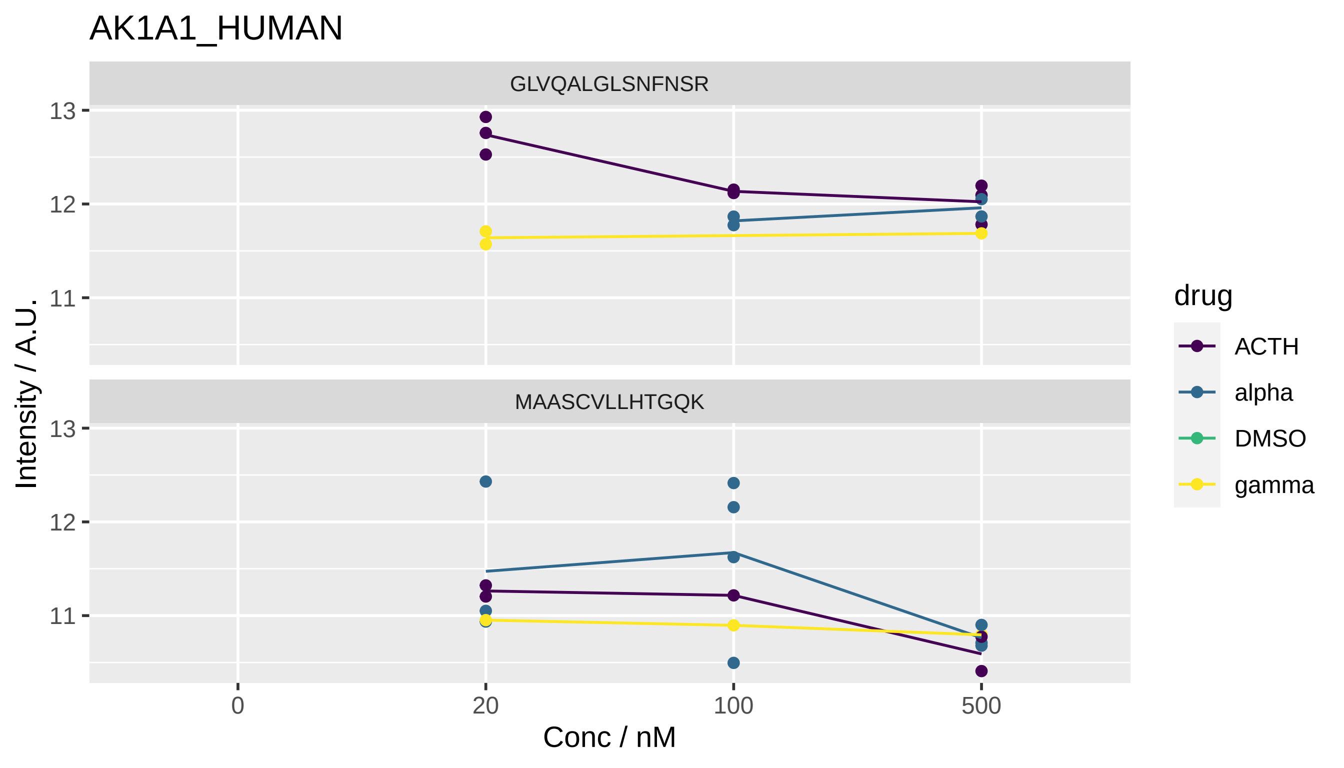
AK1A1_HUMAN
GLVQALGLSNFNSR
MAASCVLLHTGQK
Intensity / A.U.
13
12
11
0
20
100
500
Conc / nM
drug
ACTH
alpha
DMSO
gamma

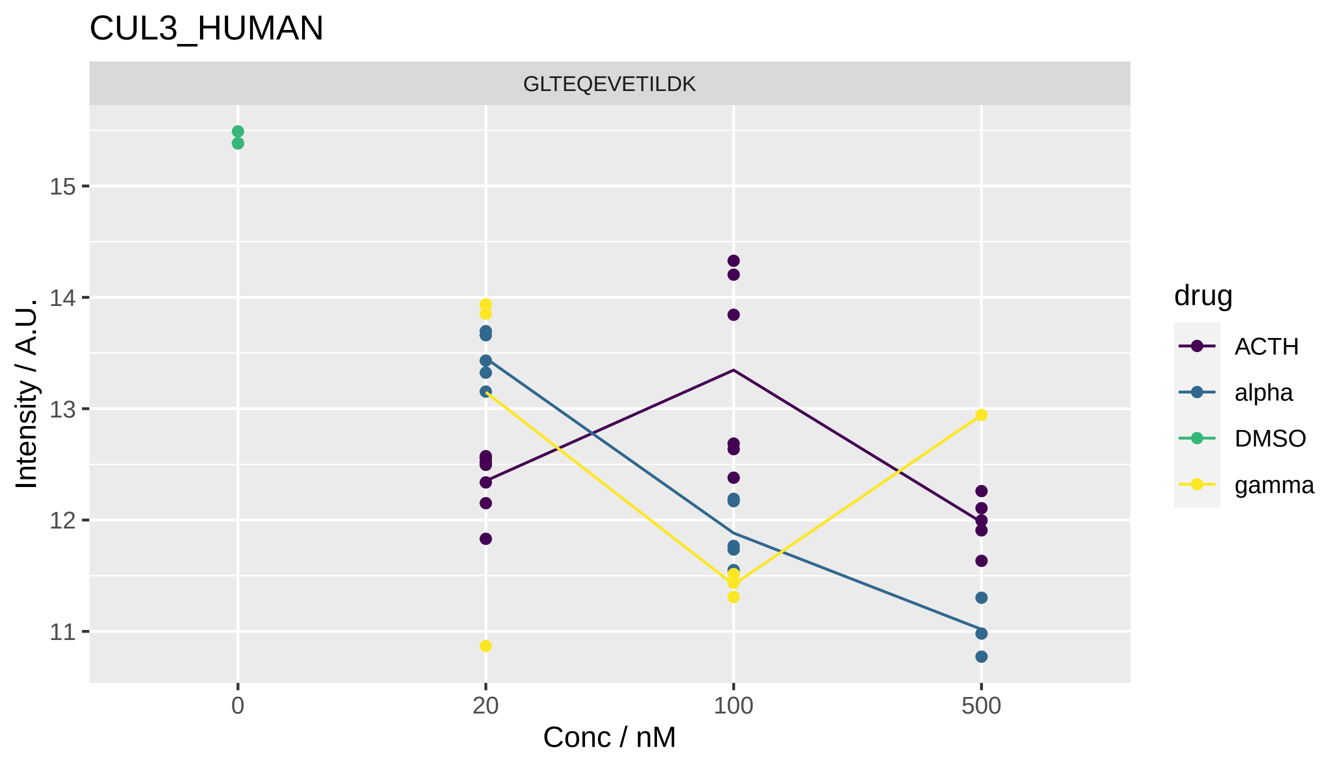

CUL3_HUMAN
GLTEQEVETILDK
Intensity / A.U.
15
14
13
12
11
0
20
100
500
Conc / nM
drug
ACTH
alpha
DMSO
gamma

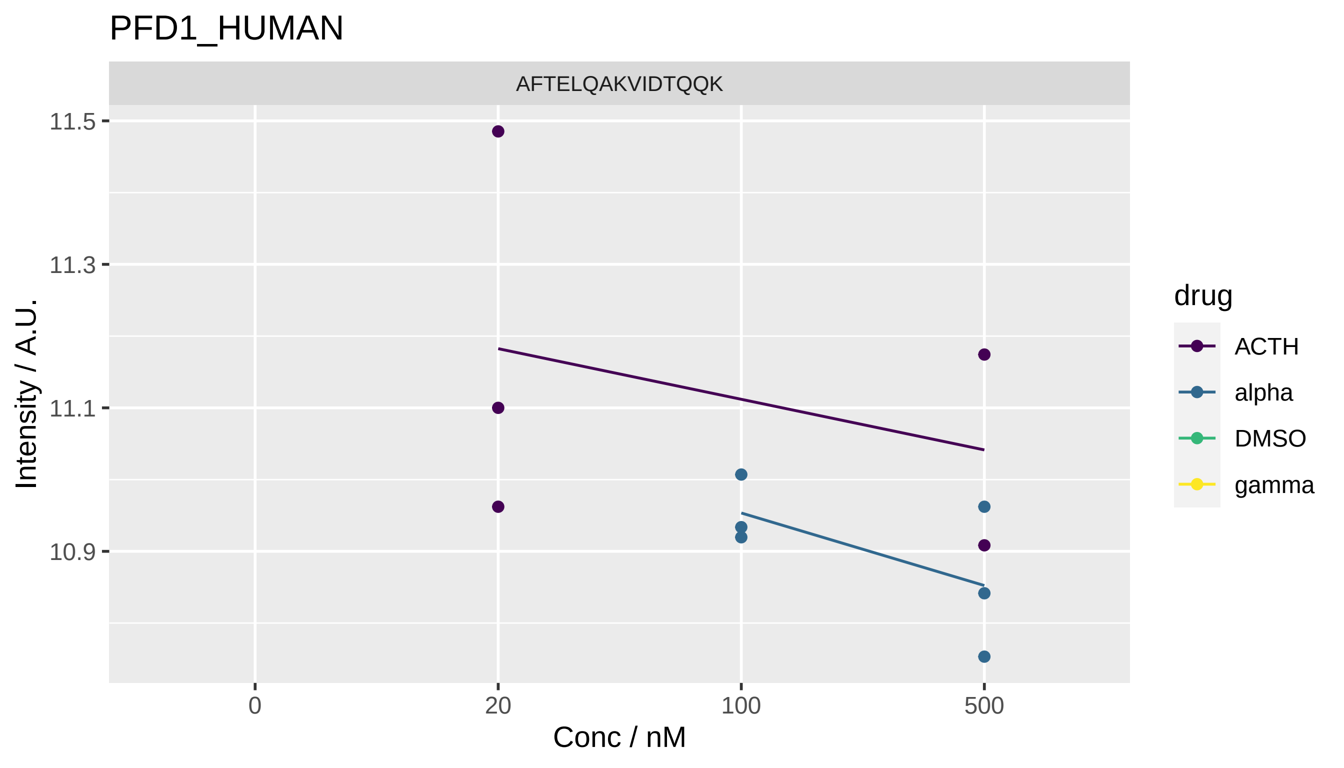
PFD1_HUMAN
AFTELQAKVIDTQQK
11.5
11.3
11.1
10.9
Intensity / A.U.
0
20
100
500
Conc / nM
drug
ACTH
alpha
DMSO
gamma

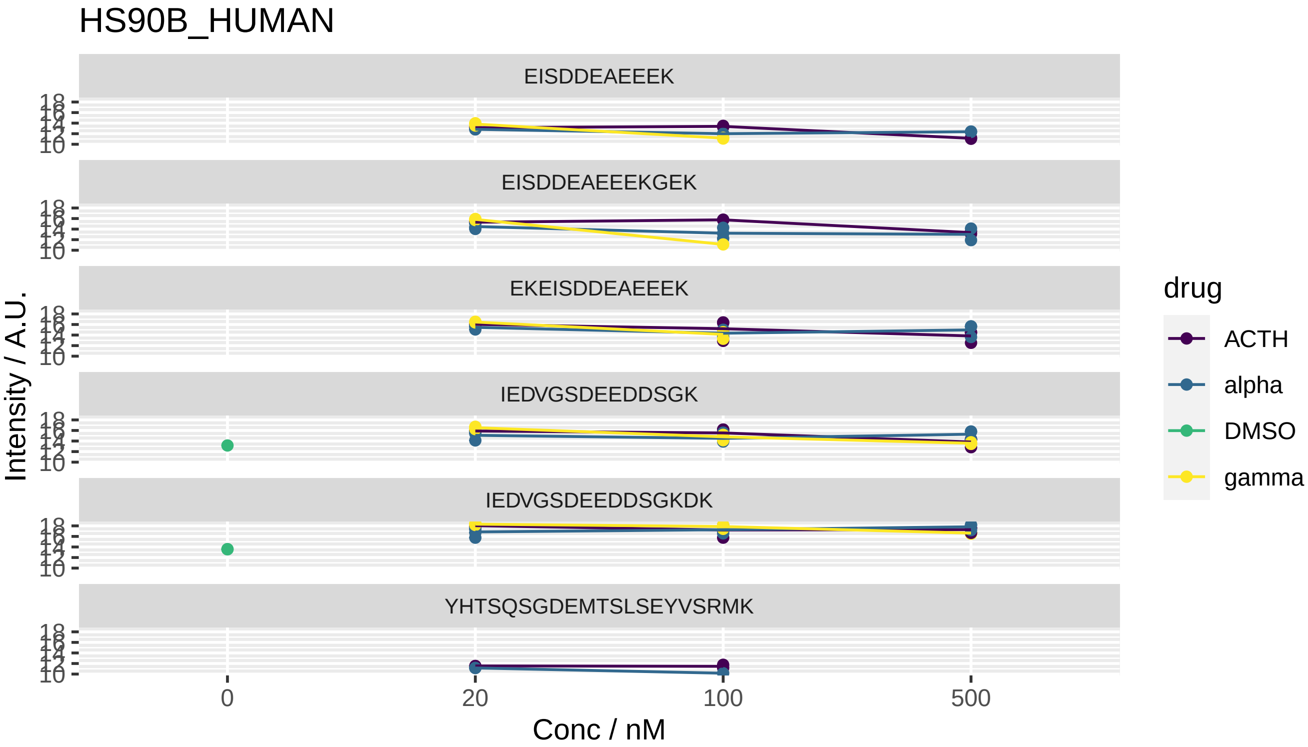

HS90B_HUMAN
EISDDEAEEEK
EISDDEAEEEKGEK
EKEISDDEAEEEK
IEDVGSDEEDDSGK
IEDVGSDEEDDSGKDK
YHTSQSGDEMTSLSEYVSRMK
10
12
14
16
18
0
20
100
500
Conc / nM
Intensity / A.U.
drug
ACTH
alpha
DMSO
gamma

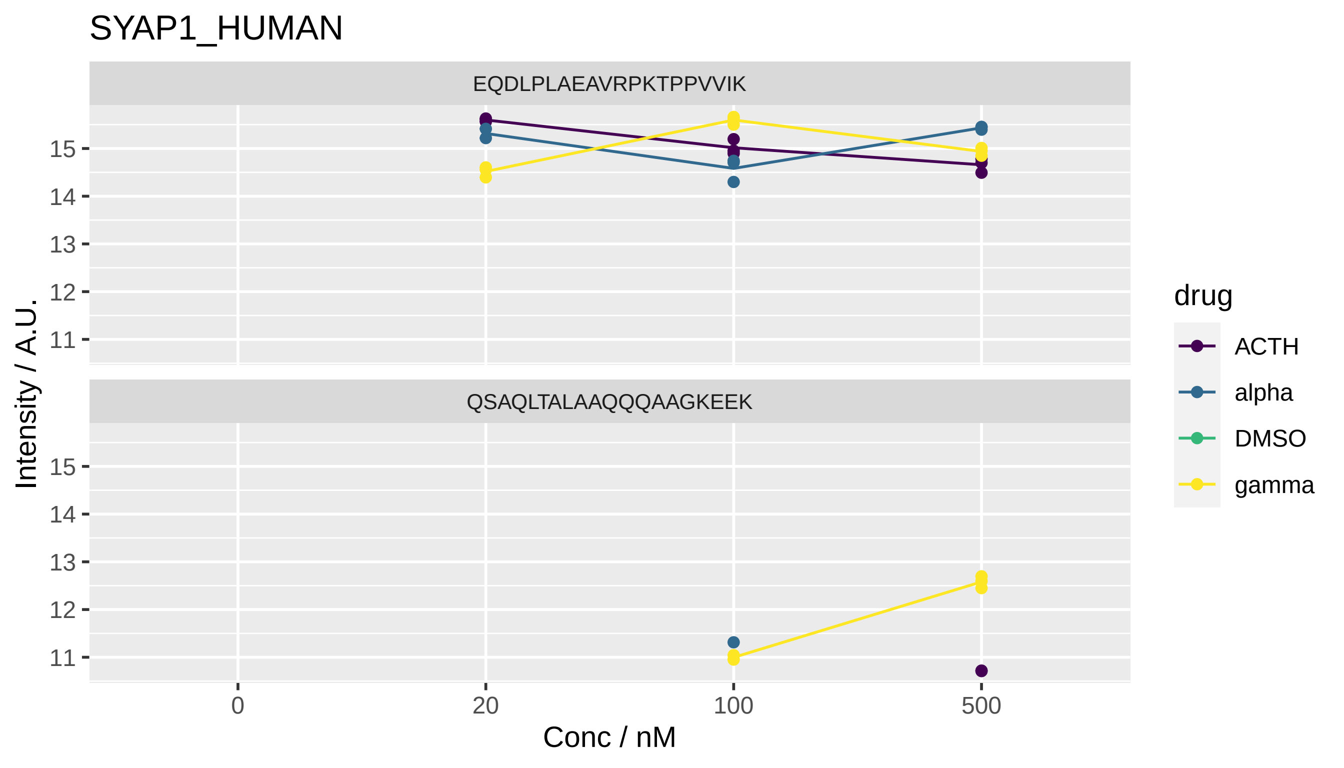
SYAP1_HUMAN
EQDLPLAEAVRPKTPPVVIK
QSAQLTALAAQQQAAGKEEK
Intensity / A.U.
15
14
13
12
11
0
20
100
500
Conc / nM
drug
ACTH
alpha
DMSO
gamma

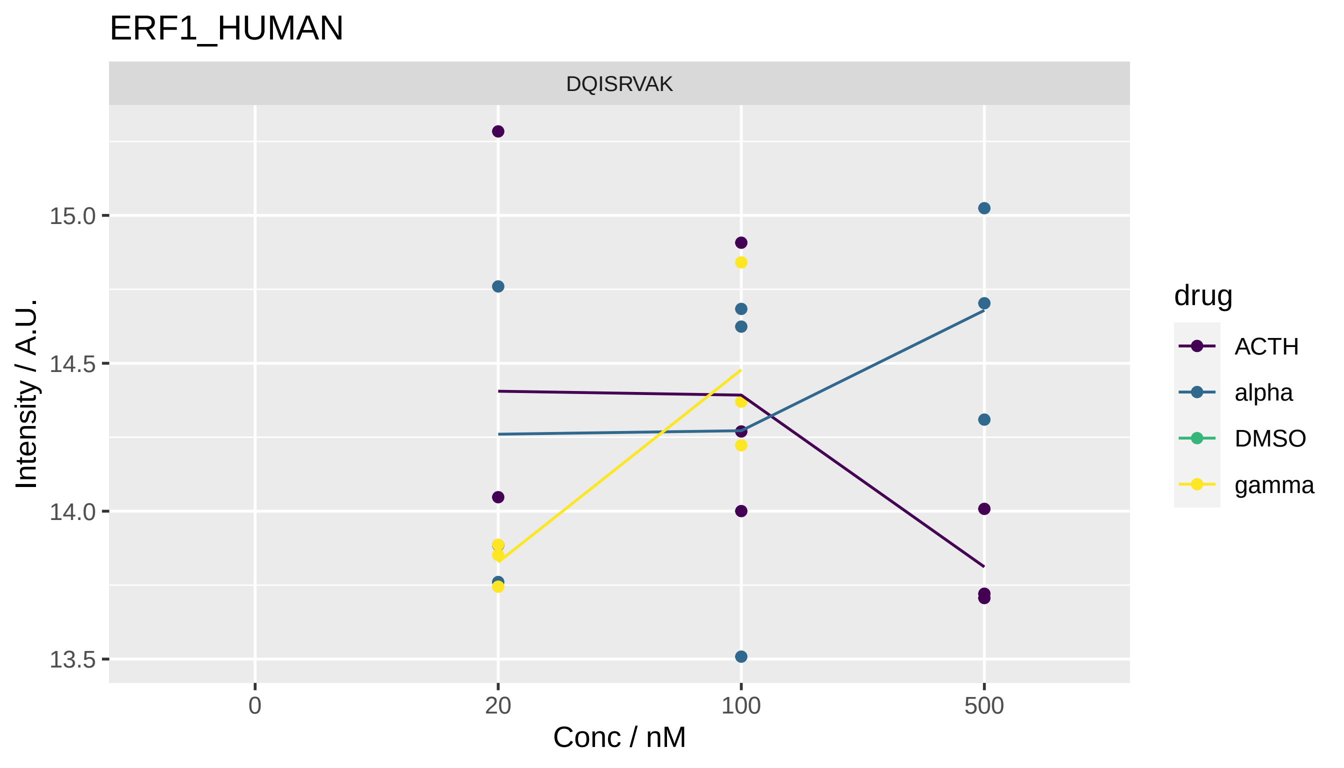
ERF1_HUMAN
DQISRVAK
Intensity / A.U.
15.0
14.5
14.0
13.5
0
20
100
500
Conc / nM
drug
ACTH
alpha
DMSO
gamma

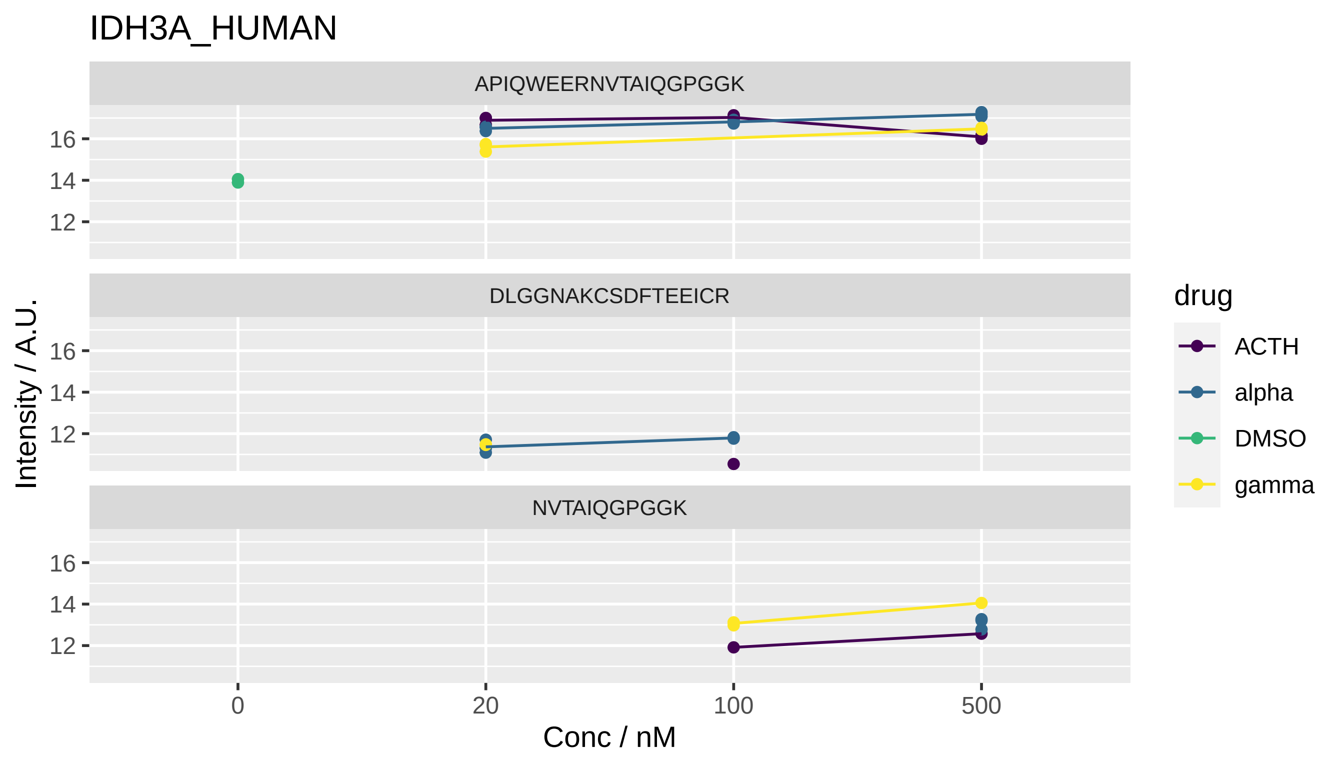

IDH3A_HUMAN
APIQWEERNVTAIQGPGGK
DLGGNAKCSDFTEEICR
NVTAIQGPGGK
Intensity / A.U.
12
14
16
0
20
100
500
Conc / nM
drug
ACTH
alpha
DMSO
gamma

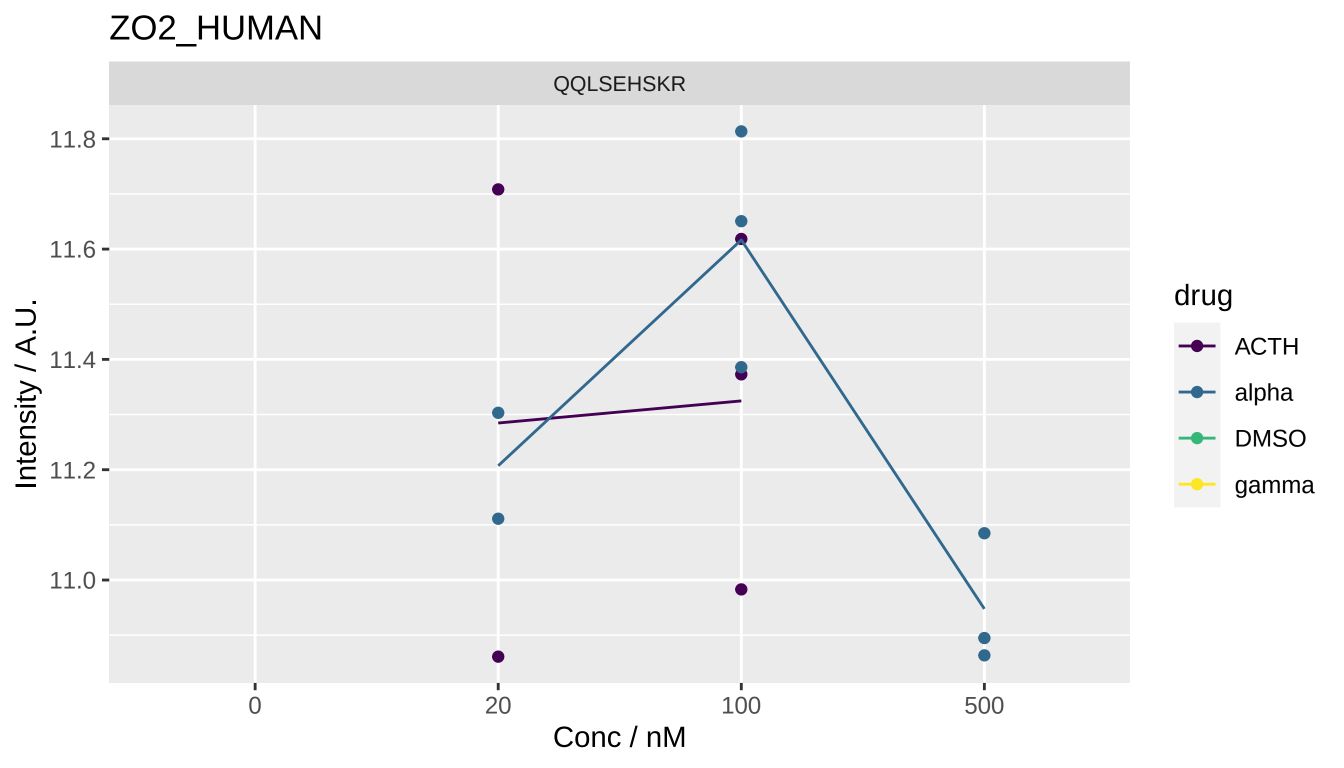
ZO2_HUMAN
QQLSEHSKR
Intensity / A.U.
11.8
11.6
11.4
11.2
11.0
0
20
100
500
Conc / nM
drug
ACTH
alpha
DMSO
gamma

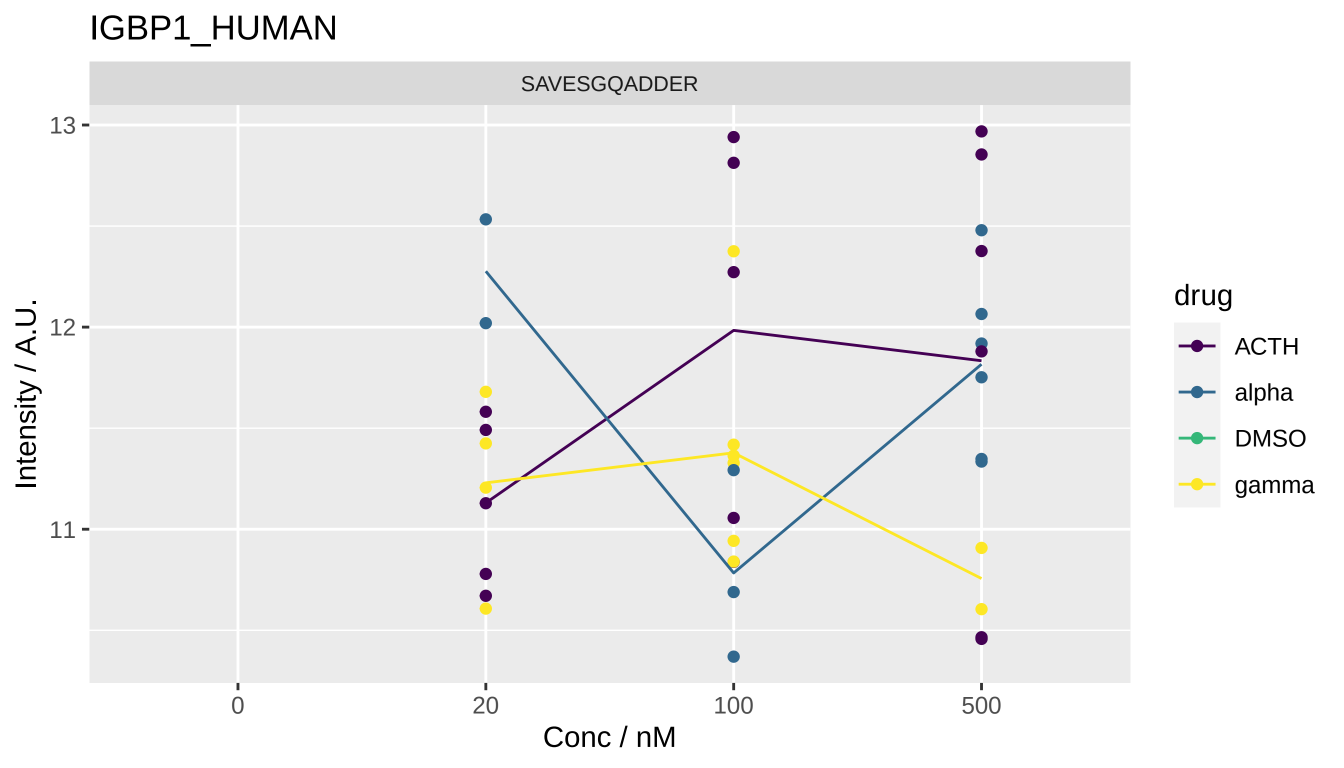
IGBP1_HUMAN
SAVESGQADDER
Intensity / A.U.
13
12
11
0
20
100
500
Conc / nM
drug
ACTH
alpha
DMSO
gamma

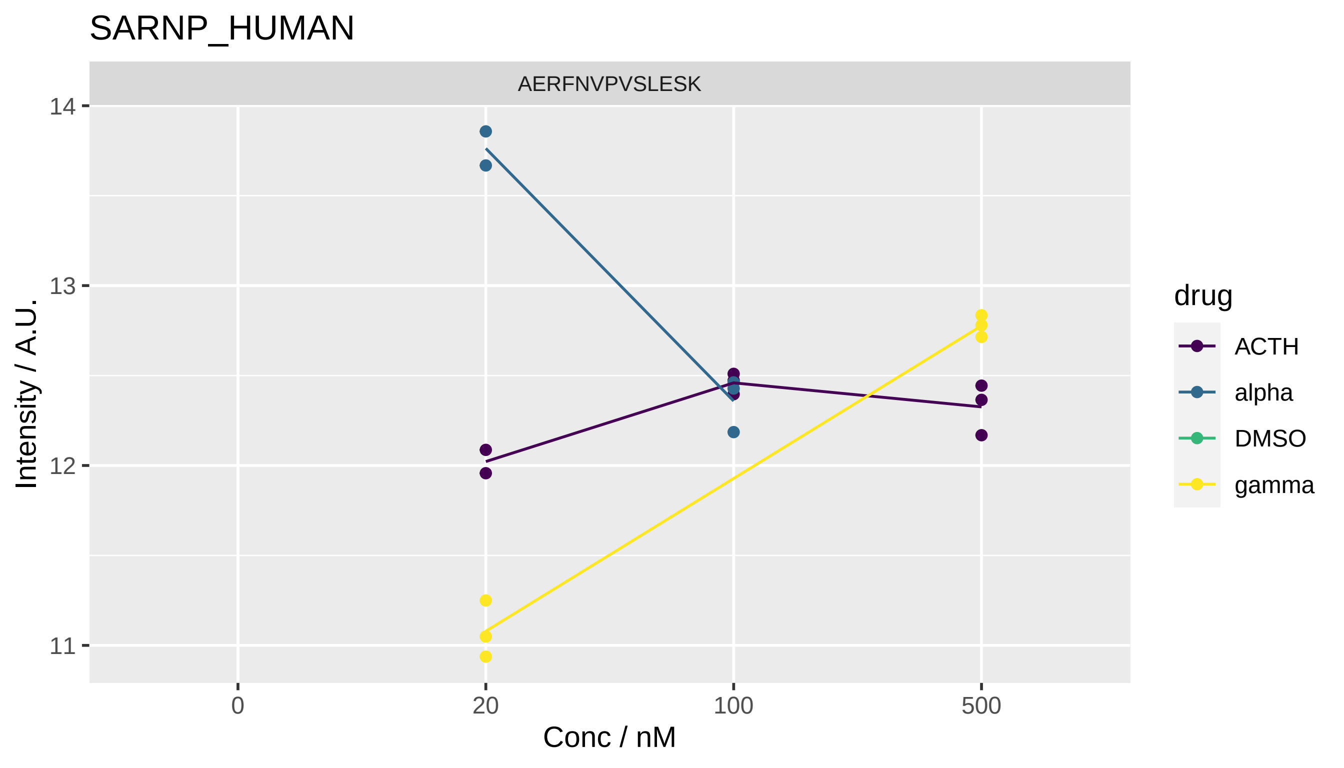

SARNP_HUMAN
AERFNVPVSLESK
14
13
12
11
Intensity / A.U.
0
20
100
500
Conc / nM
drug
ACTH
alpha
DMSO
gamma

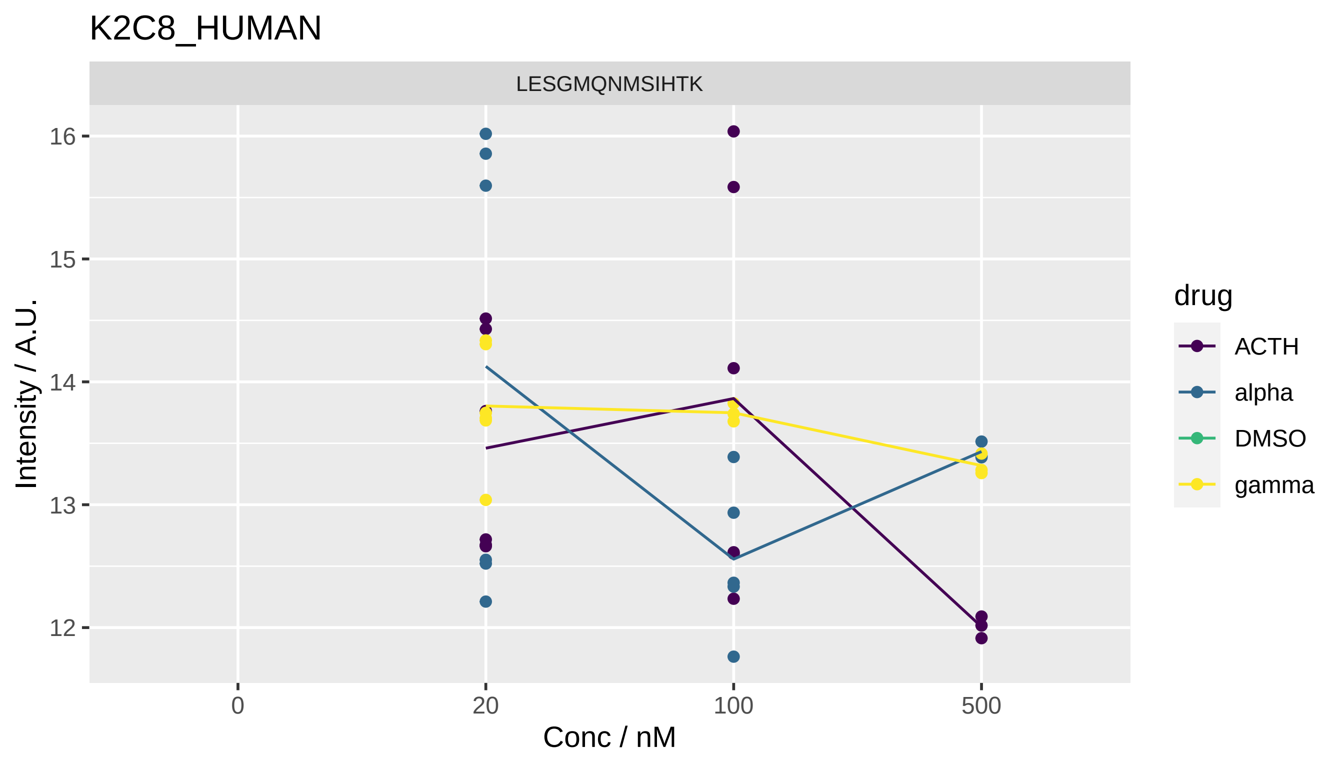

K2C8_HUMAN
LESGMQNMSIHTK
Intensity / A.U.
16
15
14
13
12
0
20
100
500
Conc / nM
drug
ACTH
alpha
DMSO
gamma

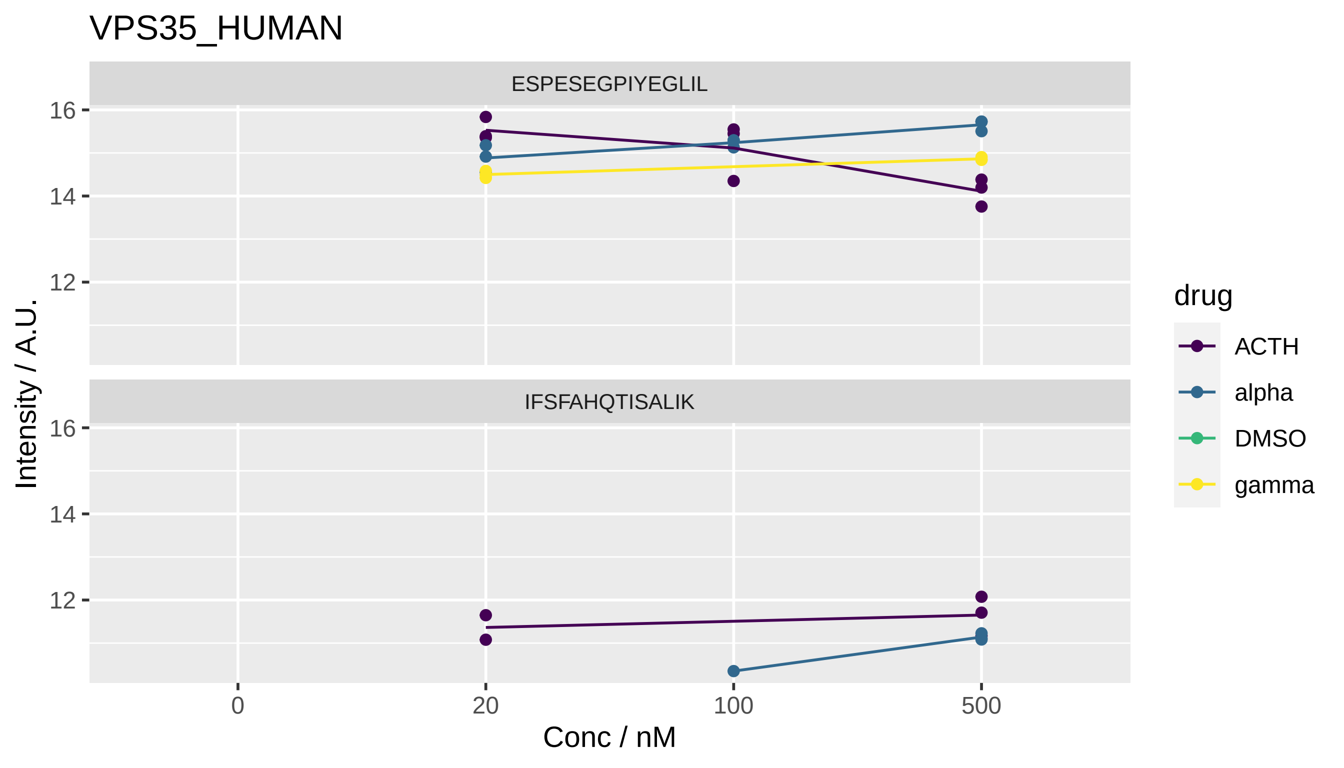
VPS35_HUMAN
ESPESEGPIYEGLIL
IFSFAHQTISALIK
Intensity / A.U.
16
14
12
0
20
100
500
Conc / nM
drug
ACTH
alpha
DMSO
gamma

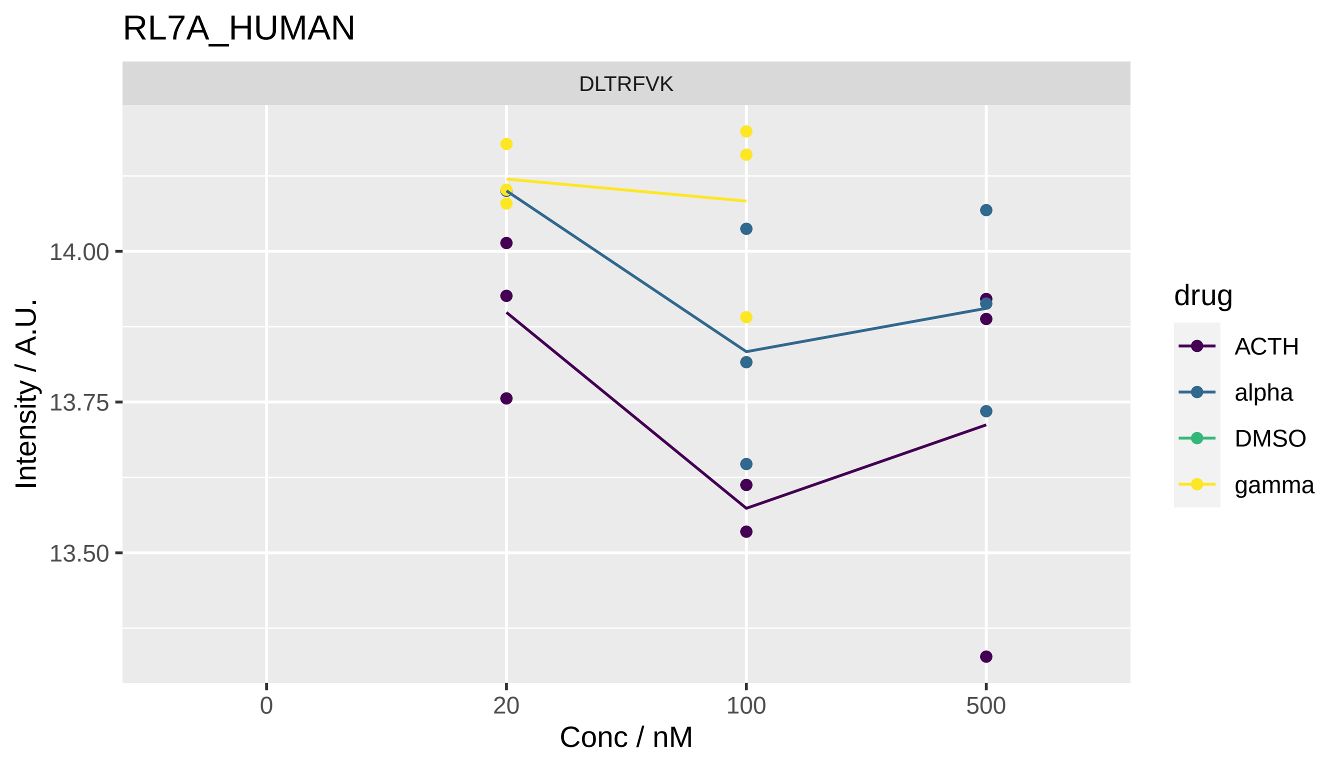
RL7A_HUMAN
DLTRFVK
Intensity / A.U.
14.00
13.75
13.50
0
20
100
500
Conc / nM
drug
ACTH
alpha
DMSO
gamma

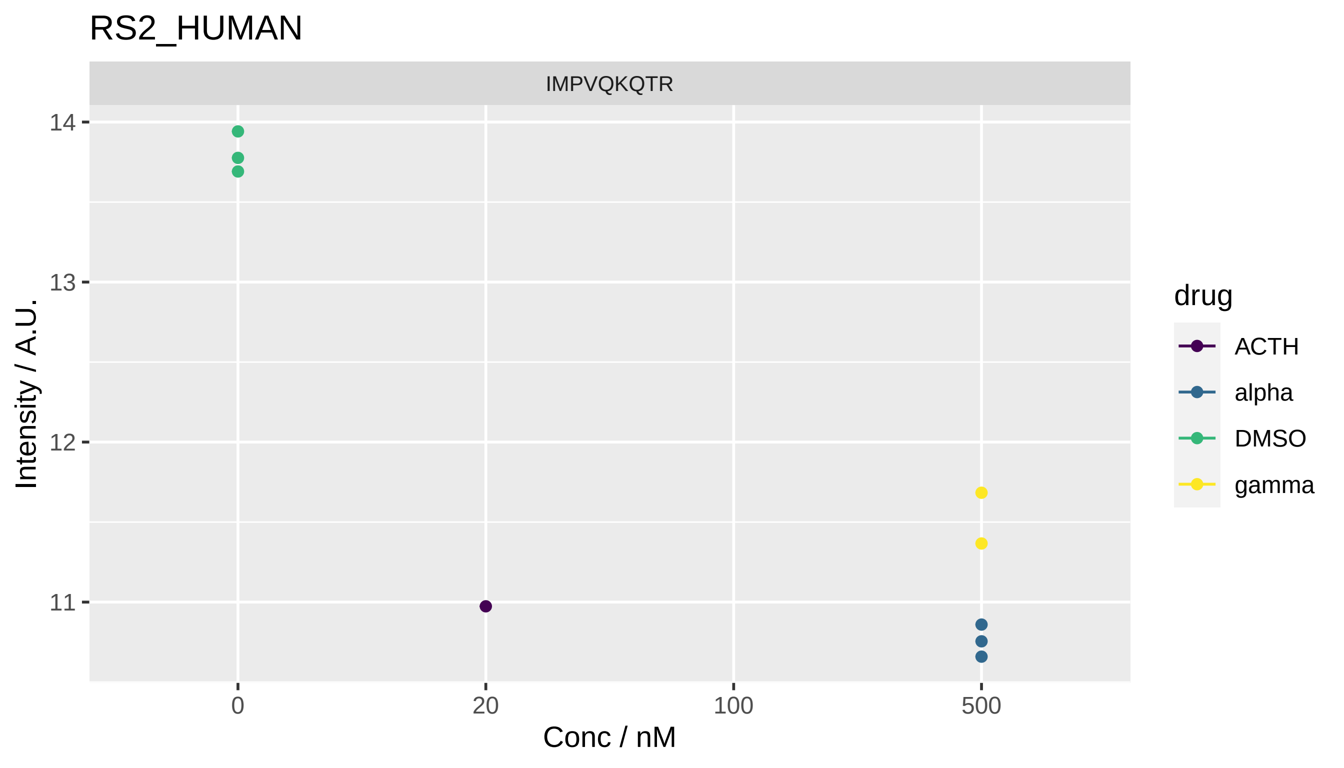
RS2_HUMAN
IMPVQKQTR
Intensity / A.U.
14
13
12
11
0
20
100
500
Conc / nM
drug
ACTH
alpha
DMSO
gamma

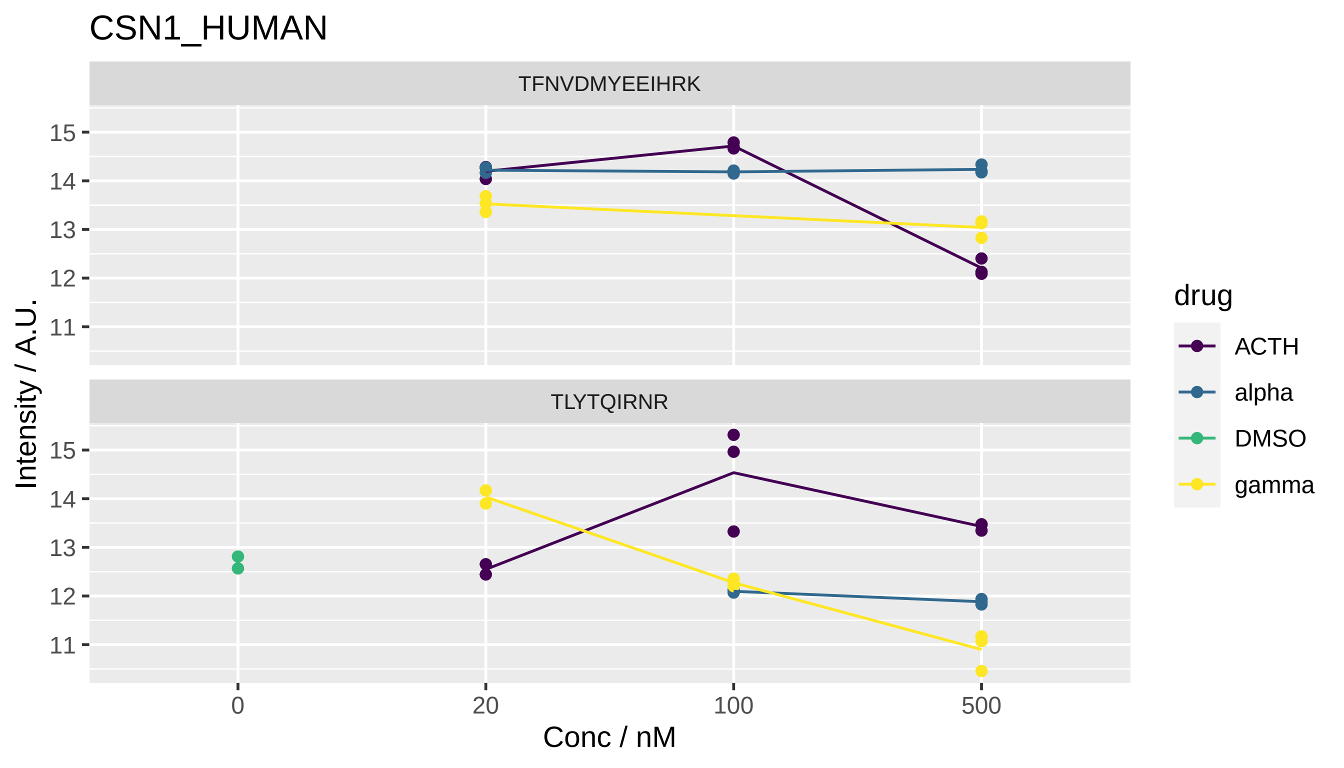

CSN1_HUMAN
TFNVDMYEEIHRK
TLYTQIRNR
Intensity / A.U.
15
14
13
12
11
0
20
100
500
Conc / nM
drug
ACTH
alpha
DMSO
gamma

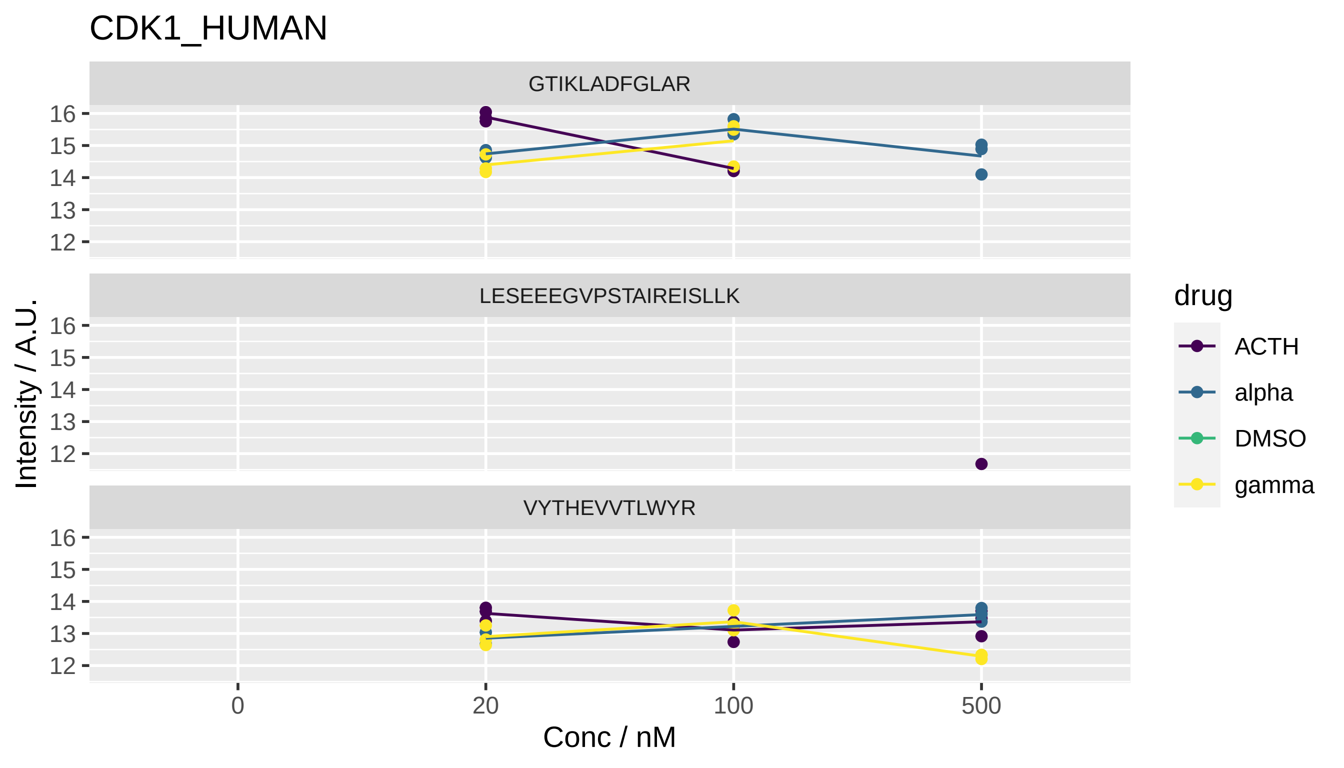
CDK1_HUMAN
GTIKLADFGLAR
LESEEEGVPSTAIREISLLK
VYTHEVVTLWYR
Intensity / A.U.
16
15
14
13
12
0
20
100
500
Conc / nM
drug
ACTH
alpha
DMSO
gamma

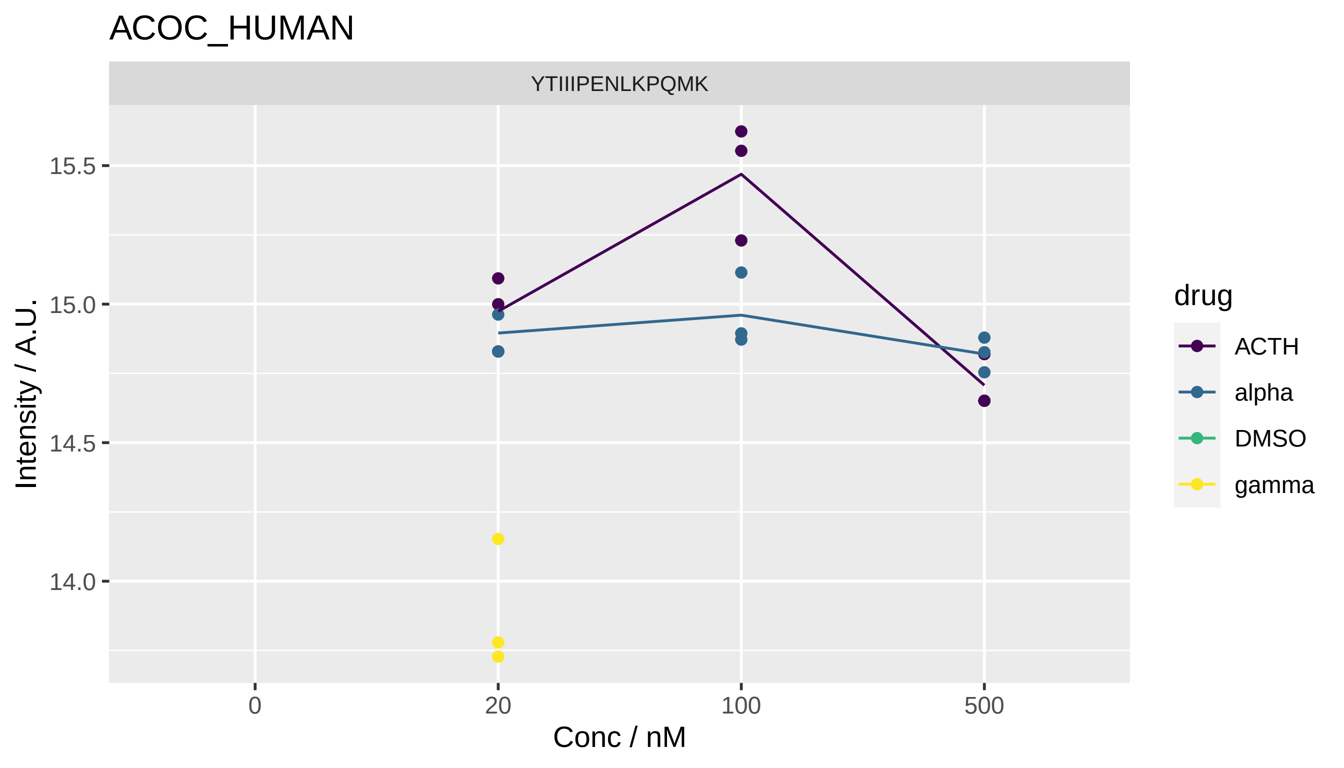

ACOC_HUMAN
YTIIIPENLKPQMK
15.5
15.0
14.5
14.0
Intensity / A.U.
0
20
100
500
Conc / nM
drug
ACTH
alpha
DMSO
gamma

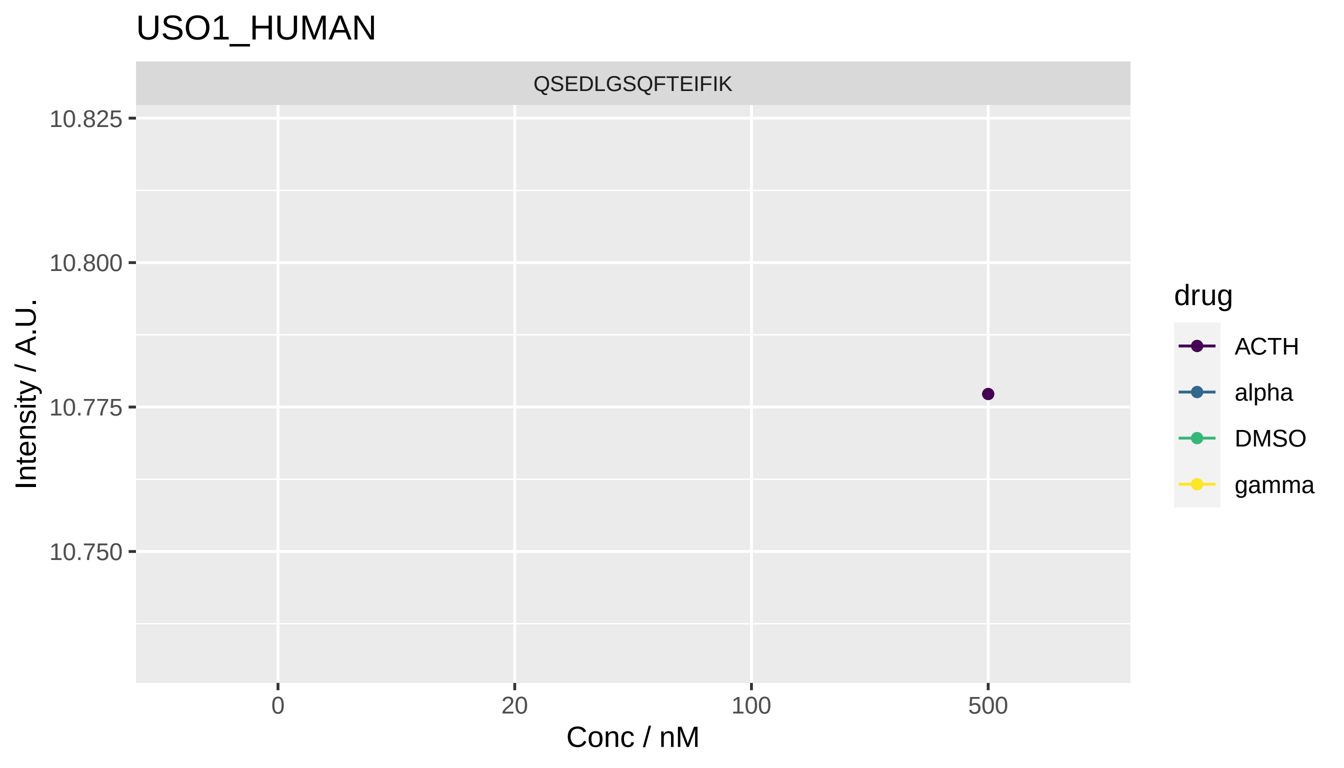

USO1_HUMAN
QSEDLGSQFTEIFIK
Intensity / A.U.
10.825
10.800
10.775
10.750
0
20
100
500
Conc / nM
drug
ACTH
alpha
DMSO
gamma

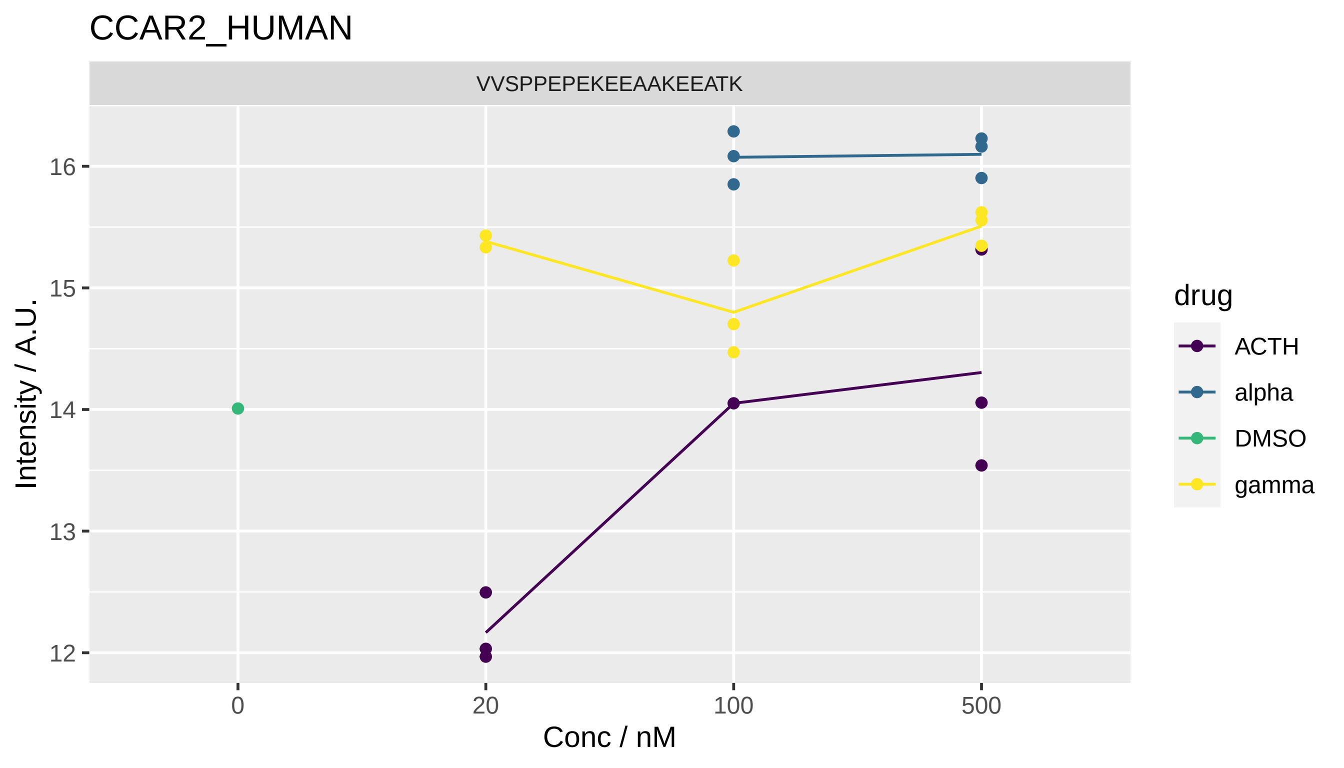

CCAR2_HUMAN
VVSPPEPEKEEAAKEEATK
Intensity / A.U.
16
15
14
13
12
0
20
100
500
Conc / nM
drug
ACTH
alpha
DMSO
gamma

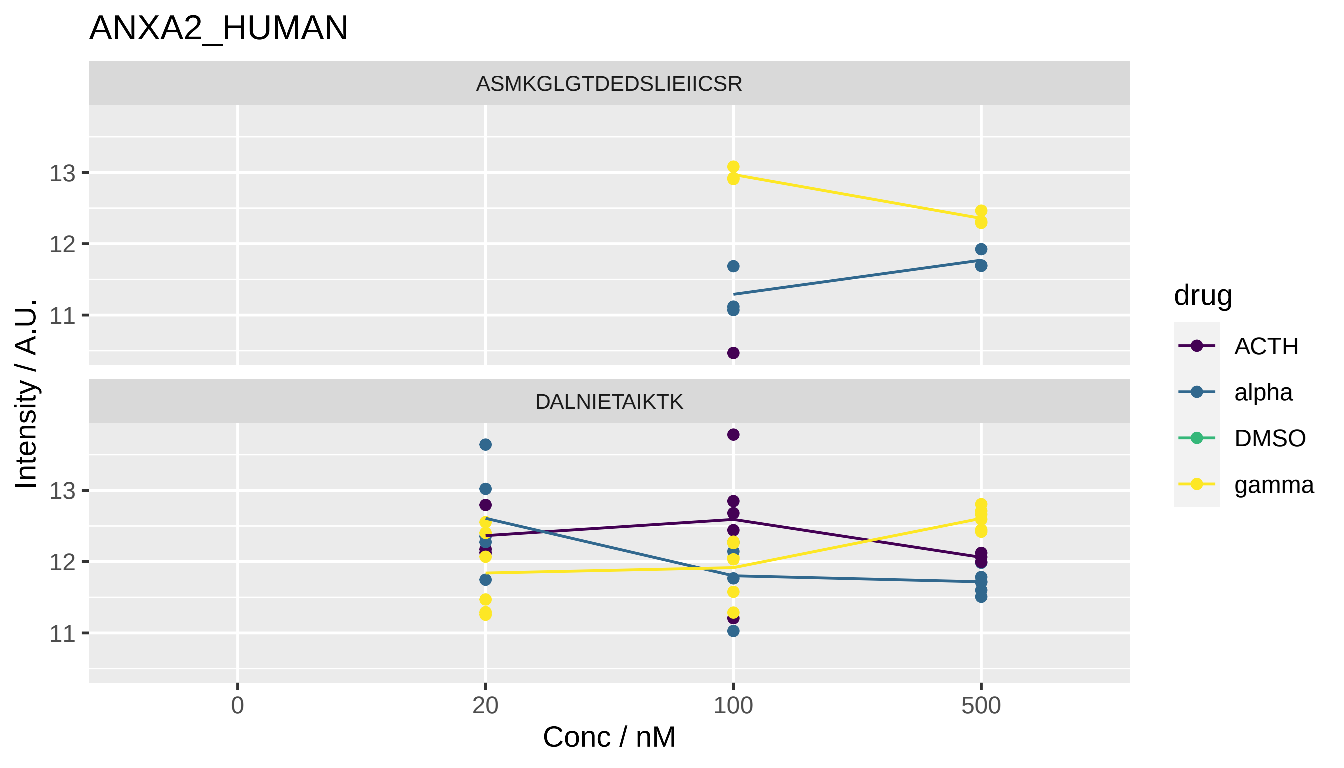

ANXA2_HUMAN
ASMKGLGTDEDSLIEIICSR
DALNIETAIKTK
Intensity / A.U.
13
12
11
13
12
11
0
20
100
500
Conc / nM
drug
ACTH
alpha
DMSO
gamma

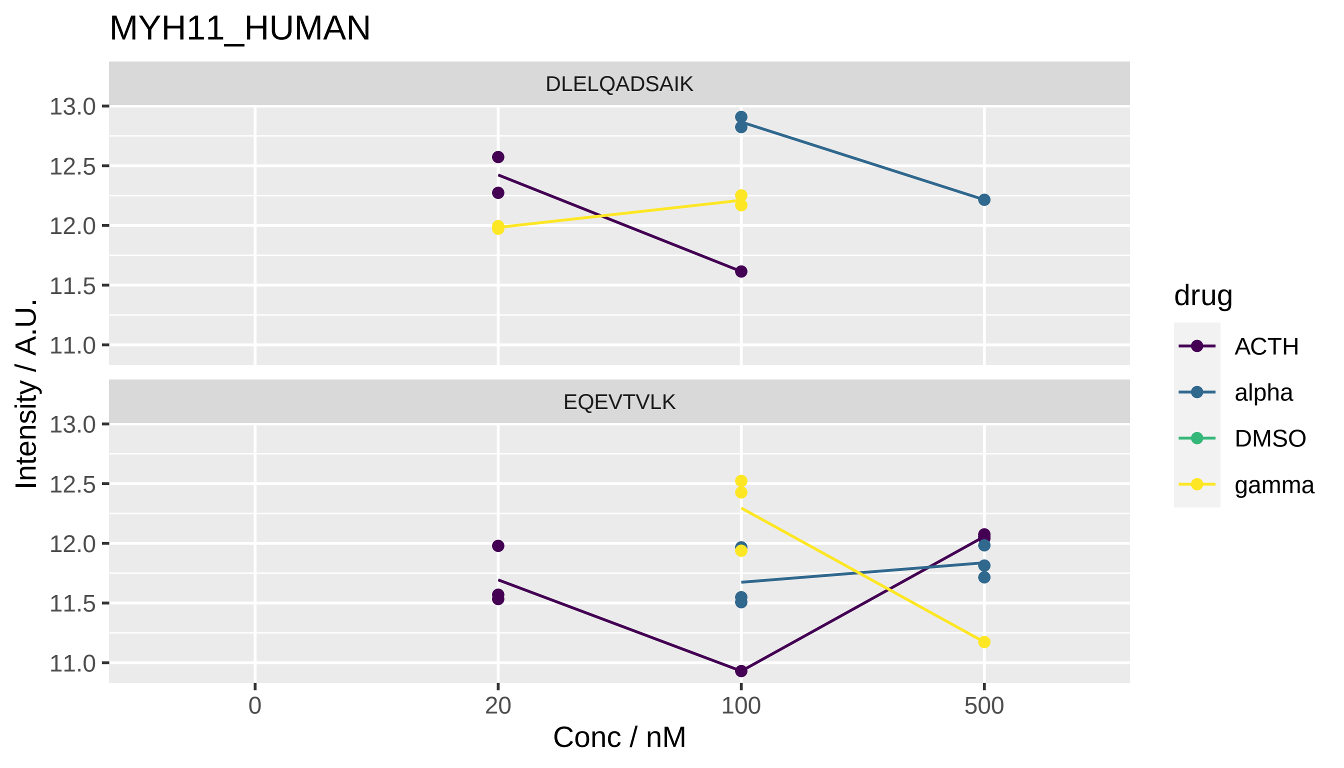

MYH11_HUMAN
DLELQADSAIK
EQEVTVLK
13.0
12.5
12.0
11.5
11.0
Intensity / A.U.
0
20
100
500
Conc / nM
drug
ACTH
alpha
DMSO
gamma

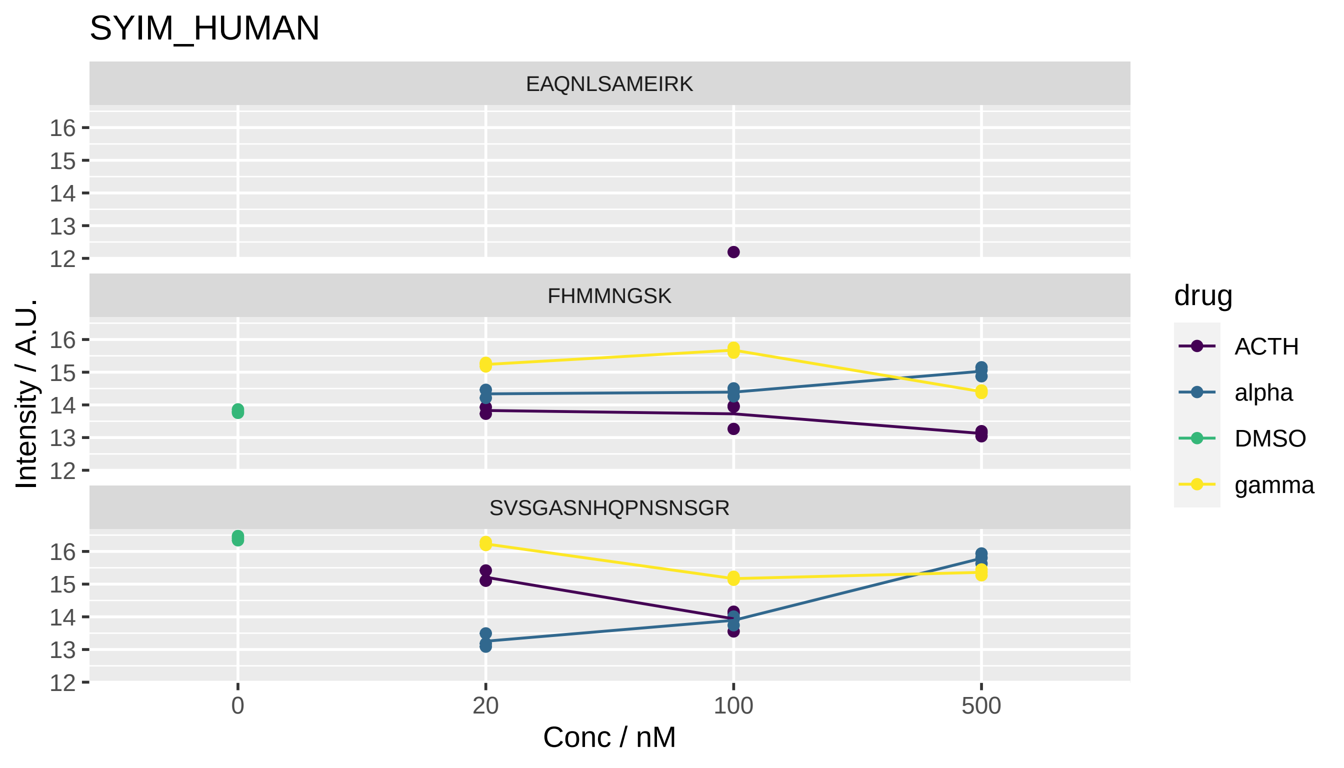
SYIM_HUMAN
EAQNLSAMEIRK
FHMMNGSK
SVSGASNHQPNSNSGR
Intensity / A.U.
16
15
14
13
12
0
20
100
500
Conc / nM
drug
ACTH
alpha
DMSO
gamma

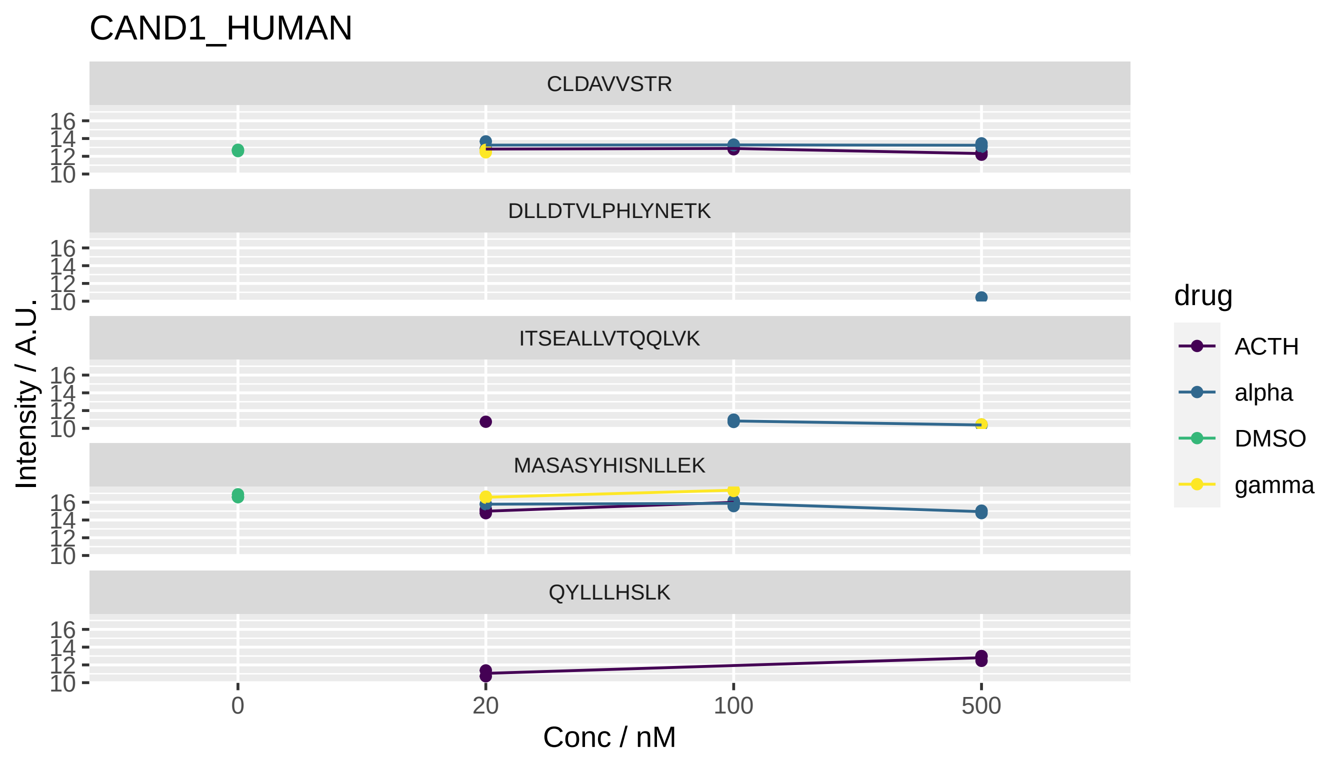

CAND1_HUMAN
CLDAVVSTR
DLLDTVLPHLYNETK
ITSEALLVTQQLVK
MASASYHISNLLEK
QYLLLHSLK
Intensity / A.U.
10
12
14
16
0
20
100
500
Conc / nM
drug
ACTH
alpha
DMSO
gamma

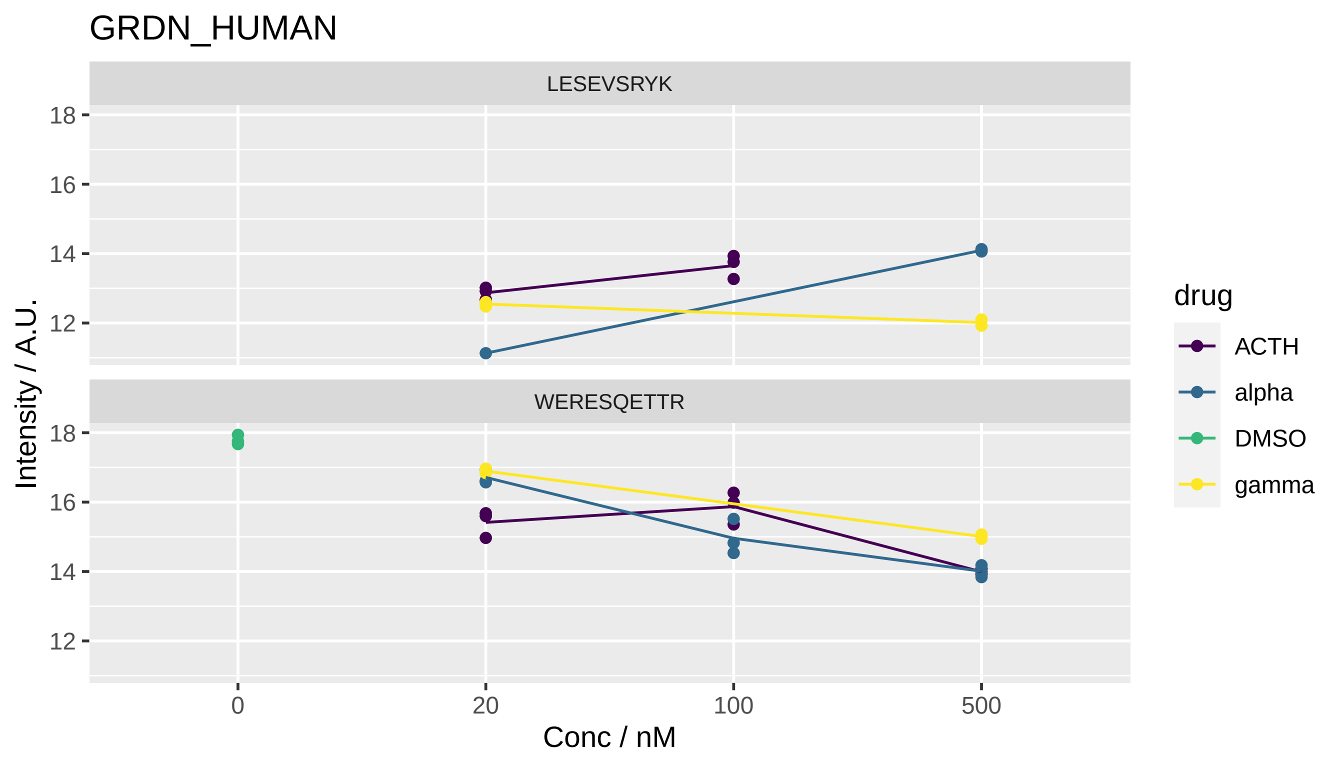

GRDN_HUMAN
LESEVSRYK
WERESQETTR
18
16
14
12
Intensity / A.U.
0
20
100
500
Conc / nM
drug
ACTH
alpha
DMSO
gamma

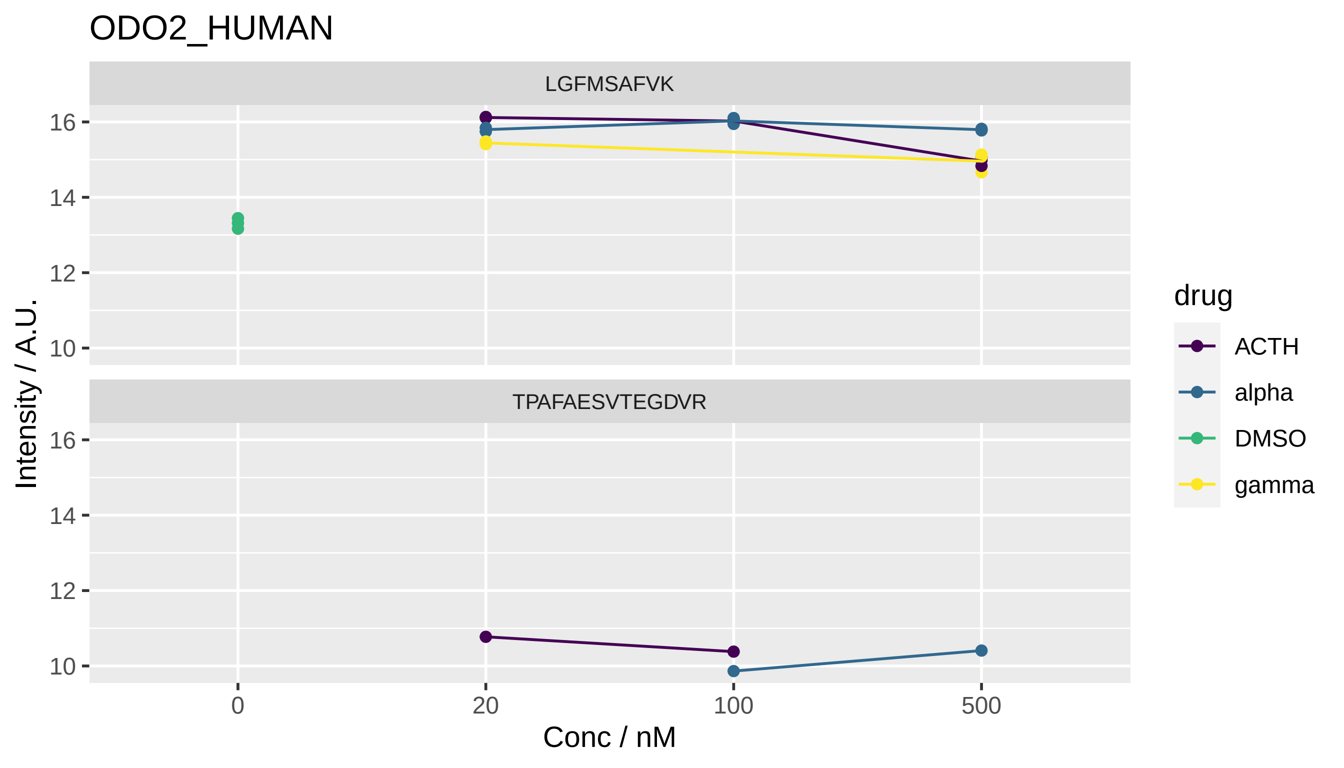
ODO2_HUMAN
LGFMSAFVK
TPAFAESVTEGDVR
Intensity / A.U.
16
14
12
10
0
20
100
500
Conc / nM
drug
ACTH
alpha
DMSO
gamma

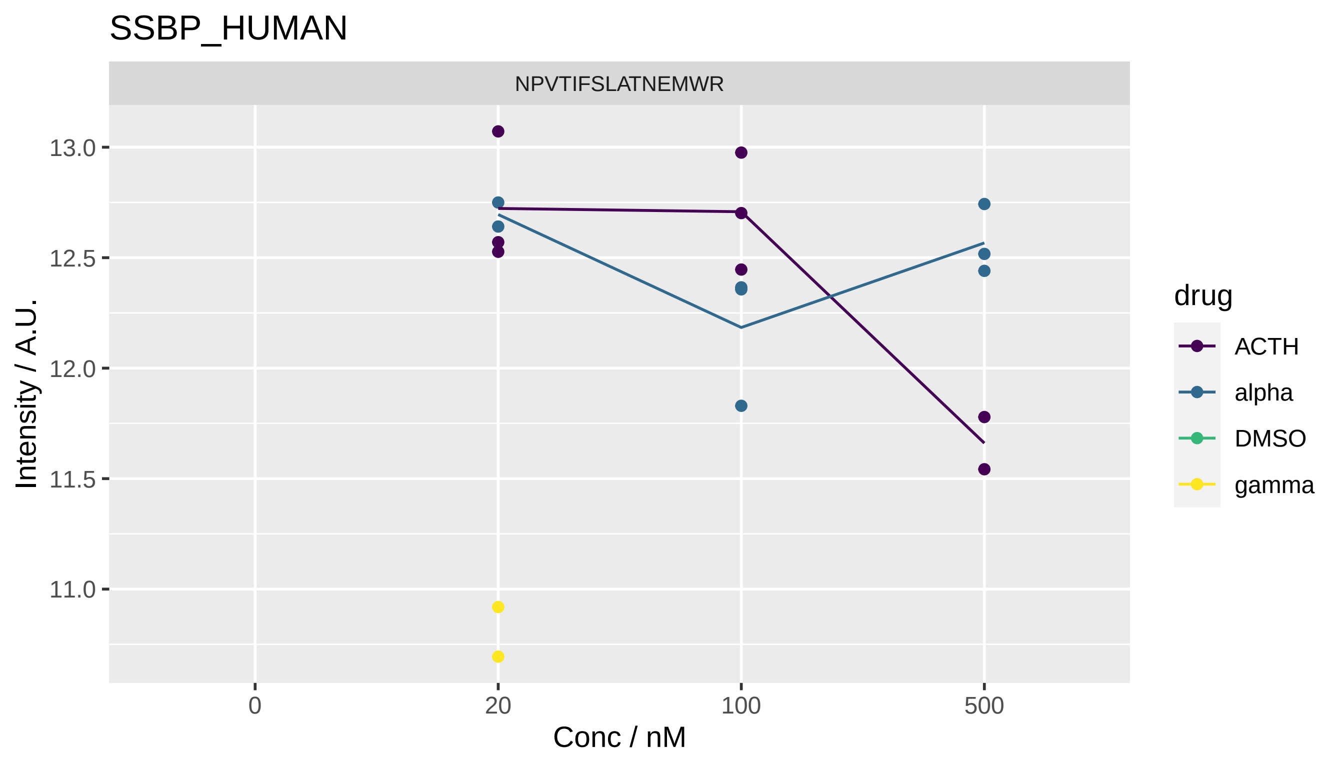
SSBP_HUMAN
NPVTIFSLATNEMWR
13.0
12.5
12.0
11.5
11.0
Intensity / A.U.
0
20
100
500
Conc / nM
drug
ACTH
alpha
DMSO
gamma

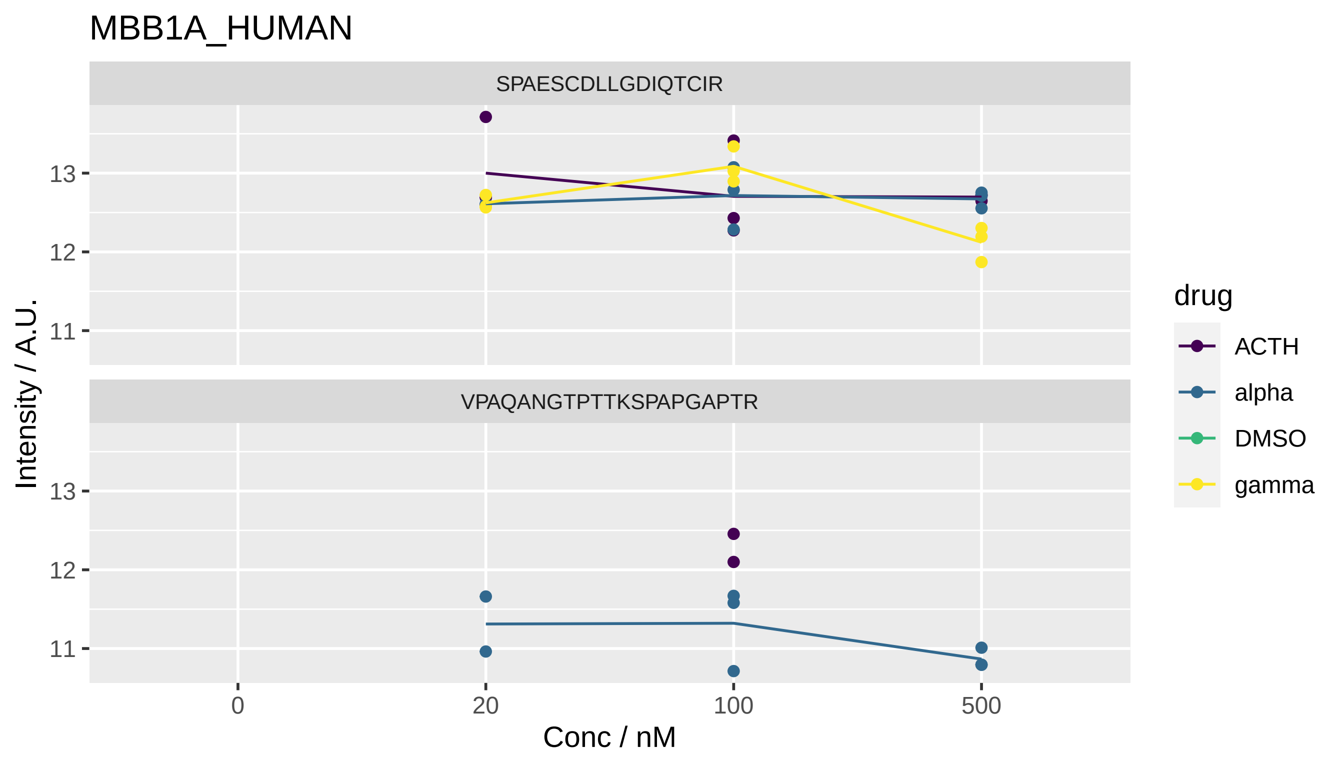

MBB1A_HUMAN
SPAESCDLLGDIQTCIR
VPAQANGTPTTKSPAPGAPTR
Intensity / A.U.
13
12
11
13
12
11
0
20
100
500
Conc / nM
drug
ACTH
alpha
DMSO
gamma

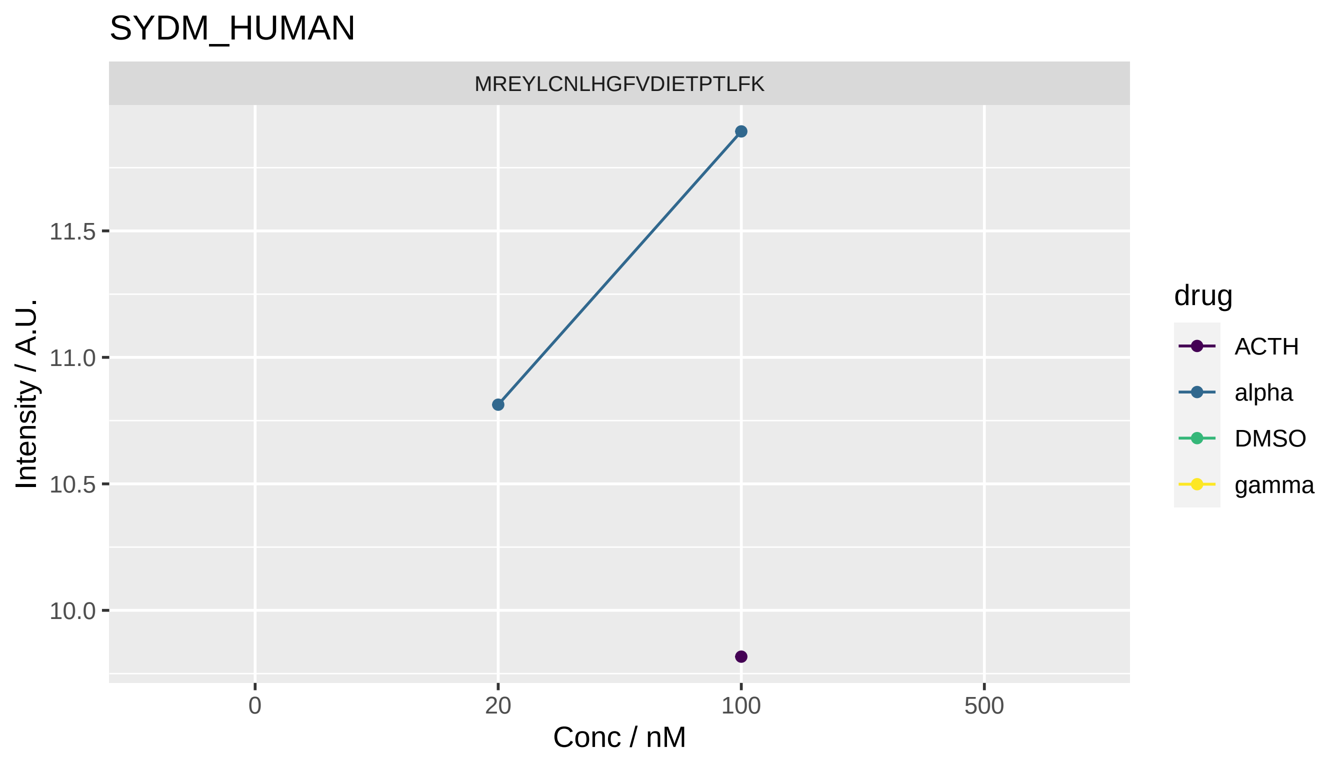
SYDM_HUMAN
MREYLCNLHGFVDIETPTLFK
Intensity / A.U.
11.5
11.0
10.5
10.0
0
20
100
500
Conc / nM
drug
ACTH
alpha
DMSO
gamma

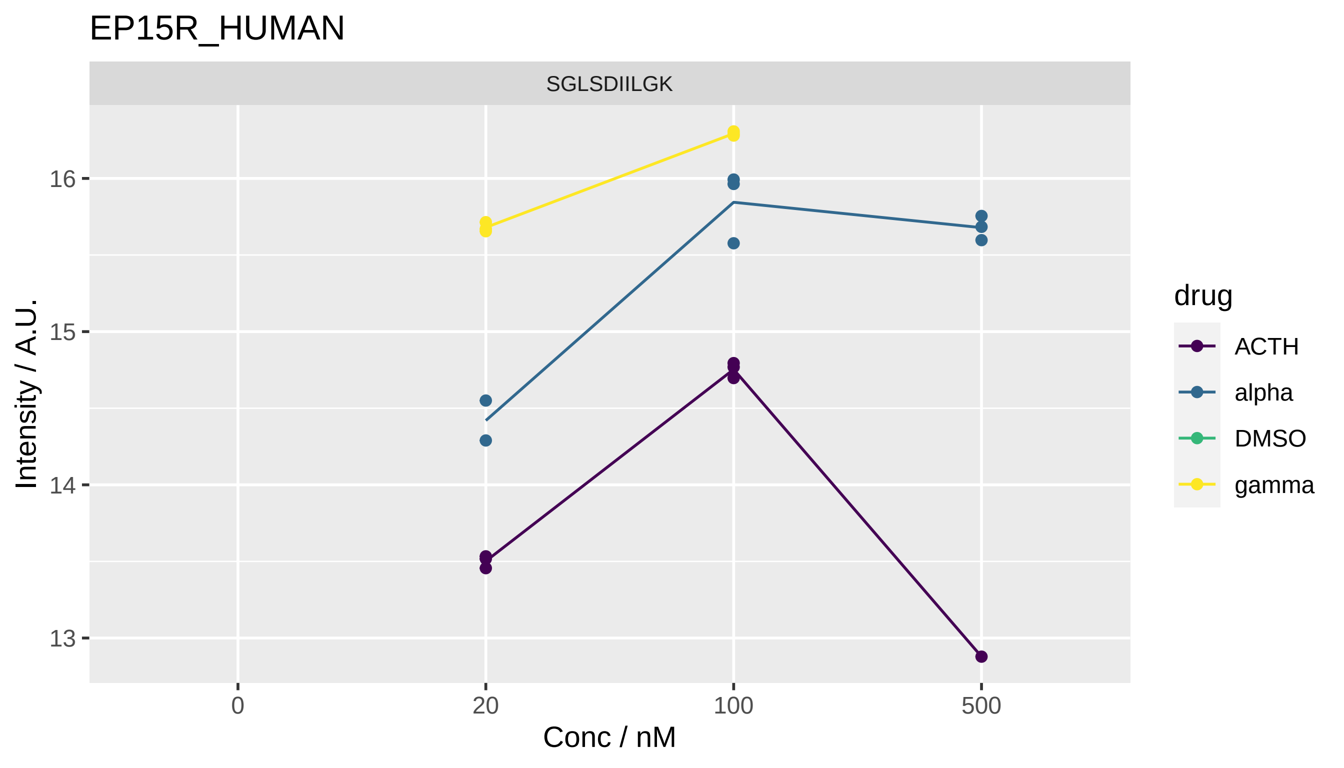
EP15R_HUMAN
SGLSDIILGK
Intensity / A.U.
16
15
14
13
0
20
100
500
Conc / nM
drug
ACTH
alpha
DMSO
gamma

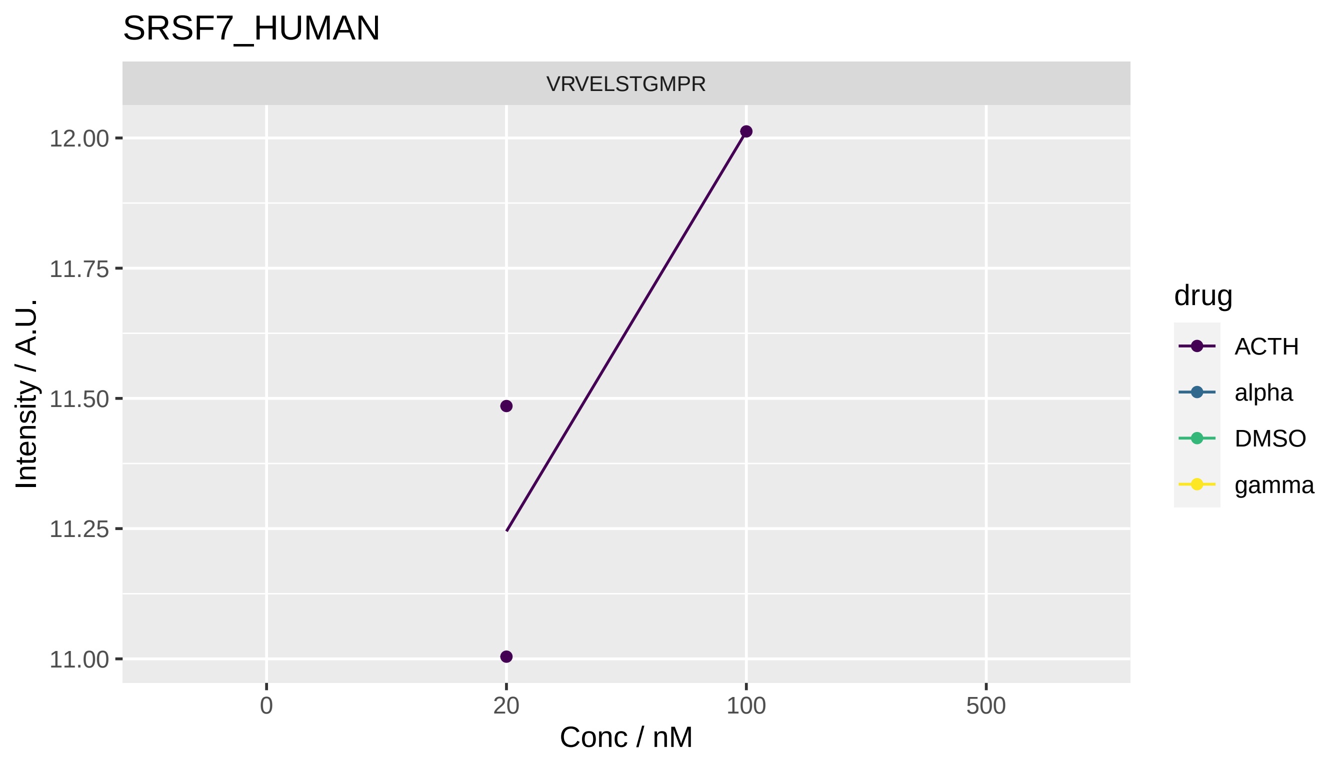
SRSF7_HUMAN
VRVELSTGMPR
12.00
11.75
11.50
11.25
11.00
Intensity / A.U.
0
20
100
500
Conc / nM
drug
ACTH
alpha
DMSO
gamma

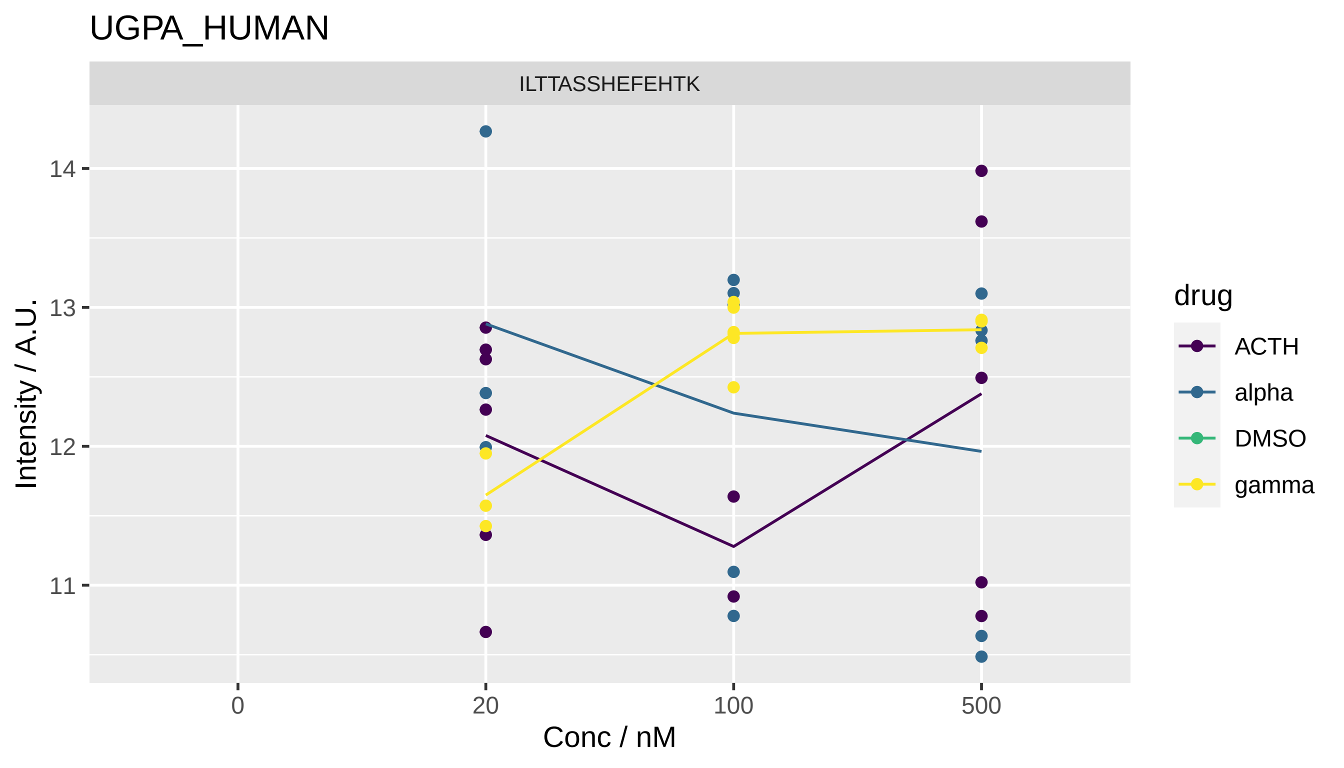
UGPA_HUMAN
ILTTASSHEFEHTK
Intensity / A.U.
14
13
12
11
0
20
100
500
Conc / nM
drug
ACTH
alpha
DMSO
gamma

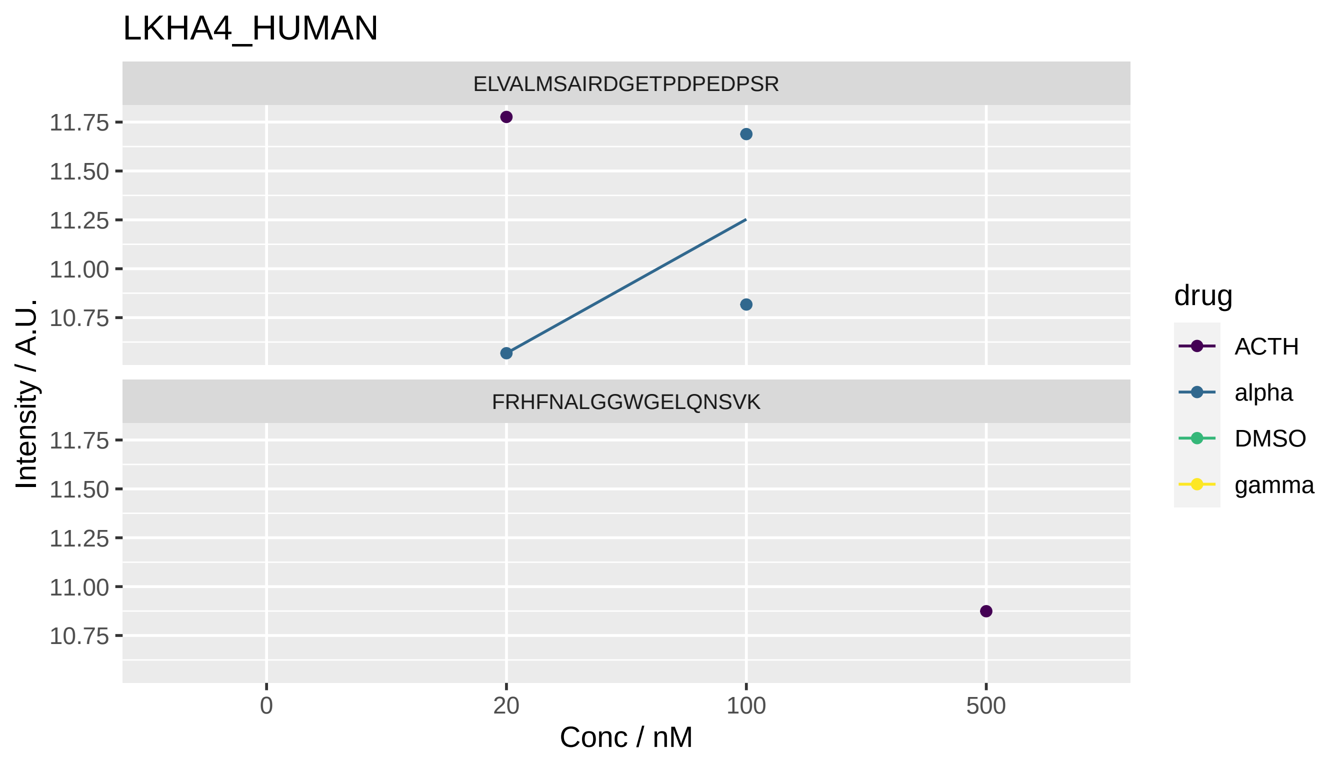
LKHA4_HUMAN
ELVALMSAIRDGETPDPEDPSR
FRHFNALGGWGELQNSVK
11.75
11.50
11.25
11.00
10.75
Intensity / A.U.
0
20
100
500
Conc / nM
drug
ACTH
alpha
DMSO
gamma

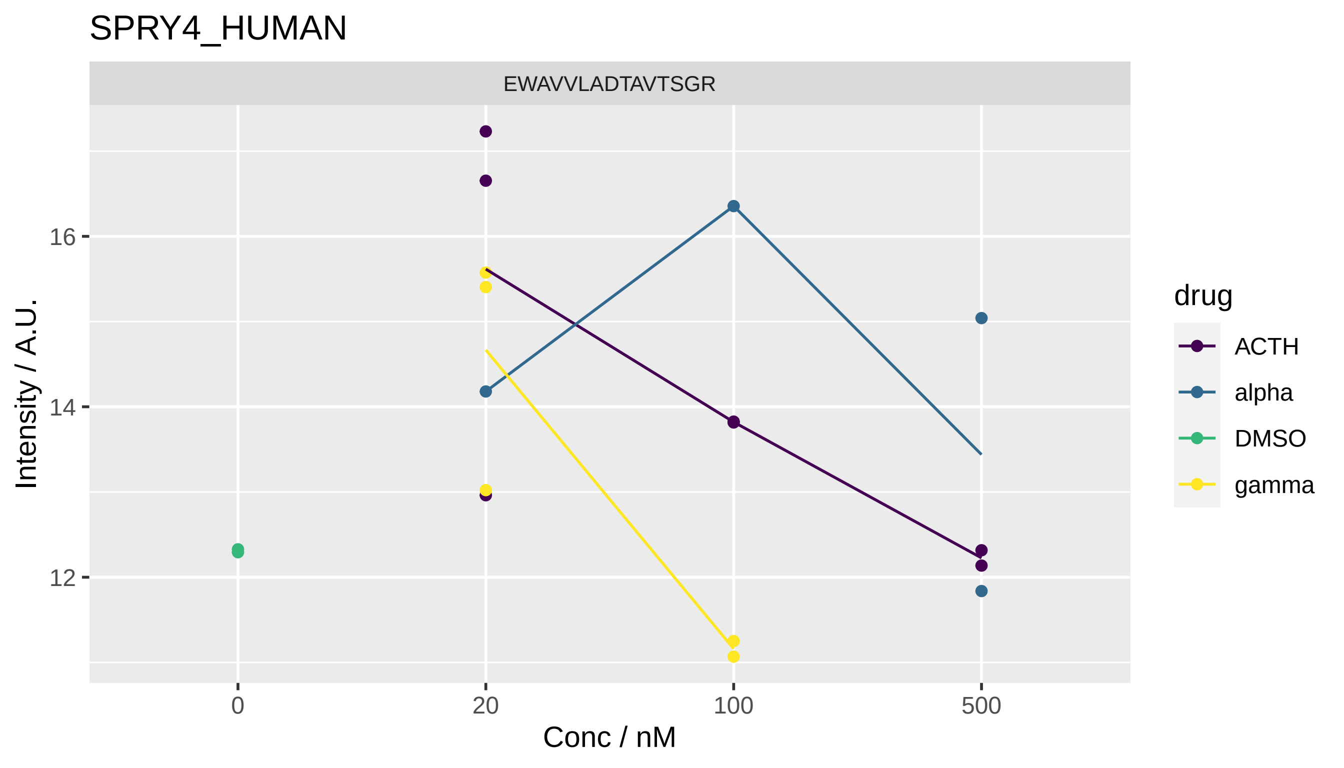
SPRY4_HUMAN
EWAVVLADTAVTSGR
Intensity / A.U.
16
14
12
0
20
100
500
Conc / nM
drug
ACTH
alpha
DMSO
gamma

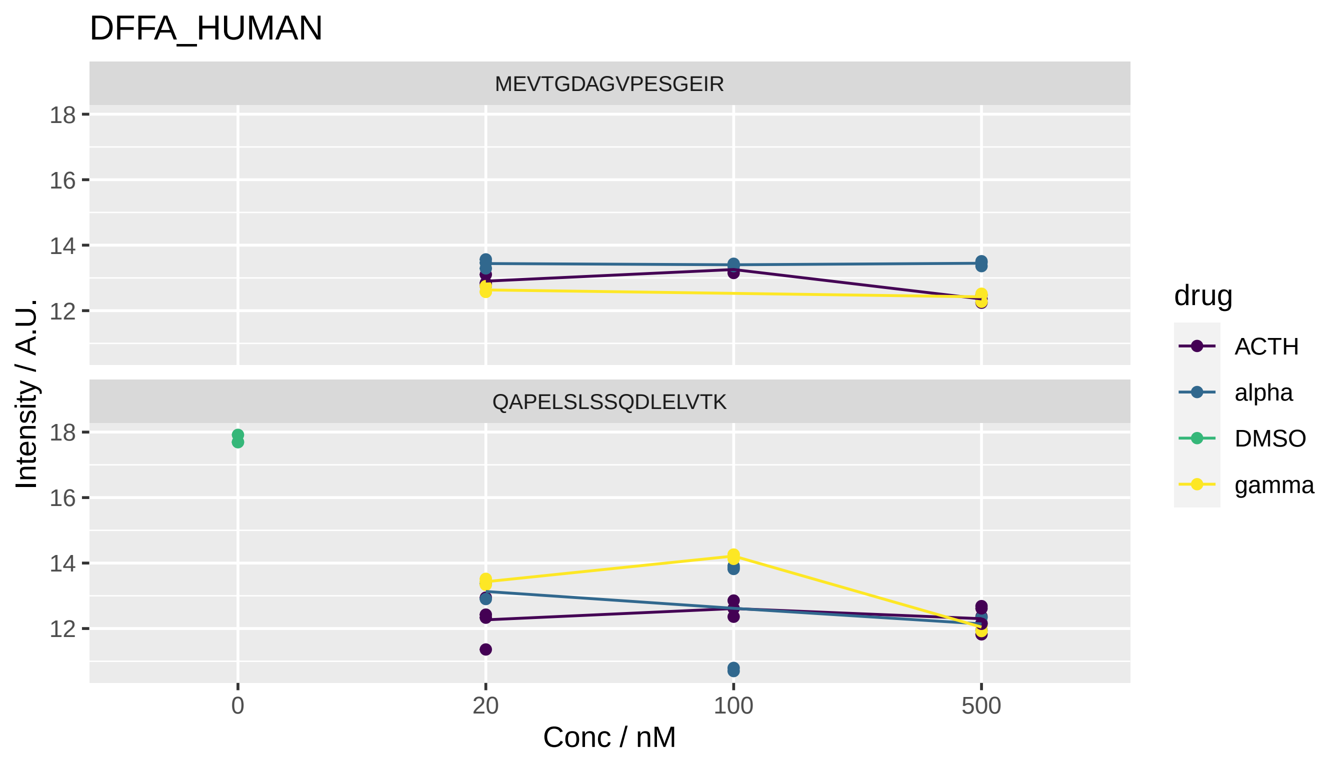

DFFA_HUMAN
MEVTGDAGVPESGEIR
QAPELSLSSQDLELVTK
Intensity / A.U.
18
16
14
12
0
20
100
500
Conc / nM
drug
ACTH
alpha
DMSO
gamma

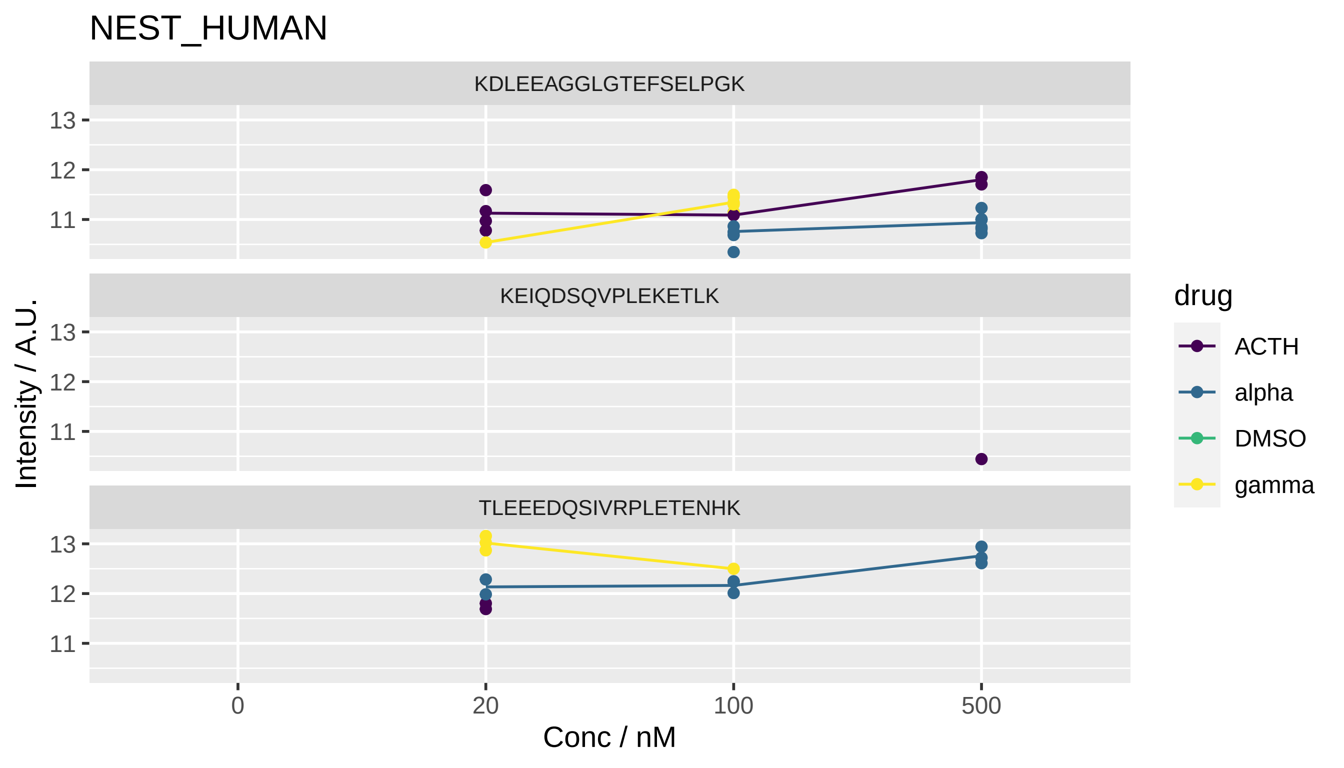

NEST_HUMAN
KDLEEAGGLGTEFSELPGK
KEIQDSQVPLEKETLK
TLEEEDQSIVRPLETENHK
13
12
11
13
12
11
13
12
11
0
20
100
500
Conc / nM
Intensity / A.U.
drug
ACTH
alpha
DMSO
gamma

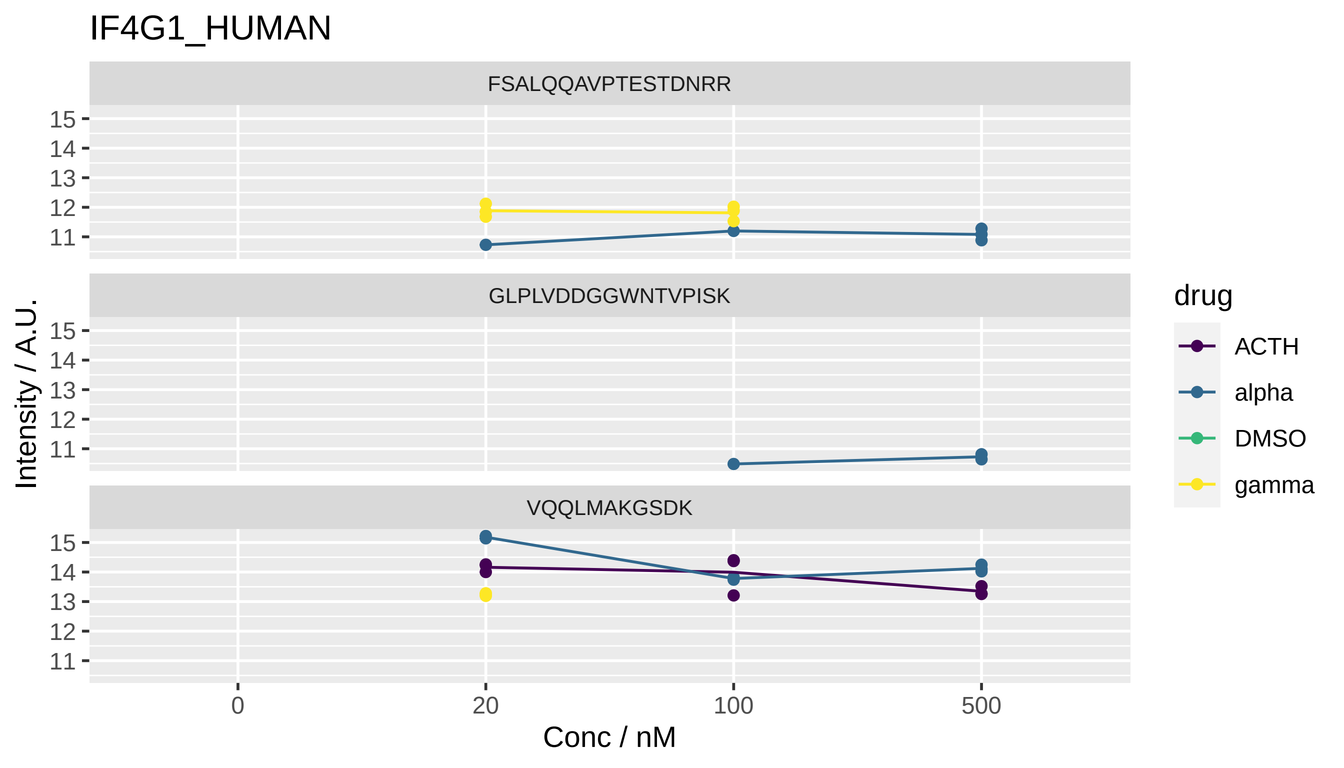

IF4G1_HUMAN
FSALQQAVPTESTDNRR
GLPLVDDGGWNTVPISK
VQQLMAKGSDK
15
14
13
12
11
Intensity / A.U.
0
20
100
500
Conc / nM
drug
ACTH
alpha
DMSO
gamma

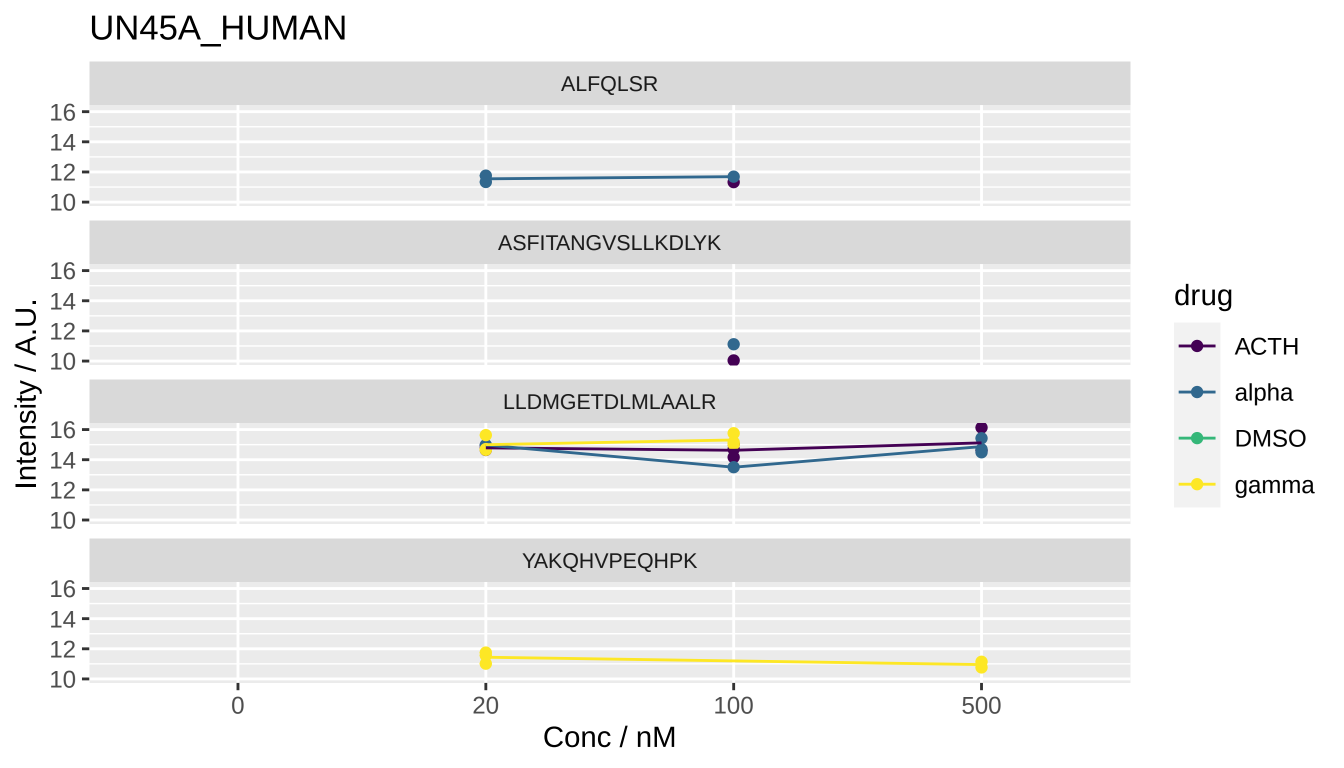

UN45A_HUMAN
ALFQLSR
ASFITANGVSLLKDLYK
LLDMGETDLMLAALR
YAKQHVPEQHPK
Intensity / A.U.
16
14
12
10
0
20
100
500
Conc / nM
drug
ACTH
alpha
DMSO
gamma

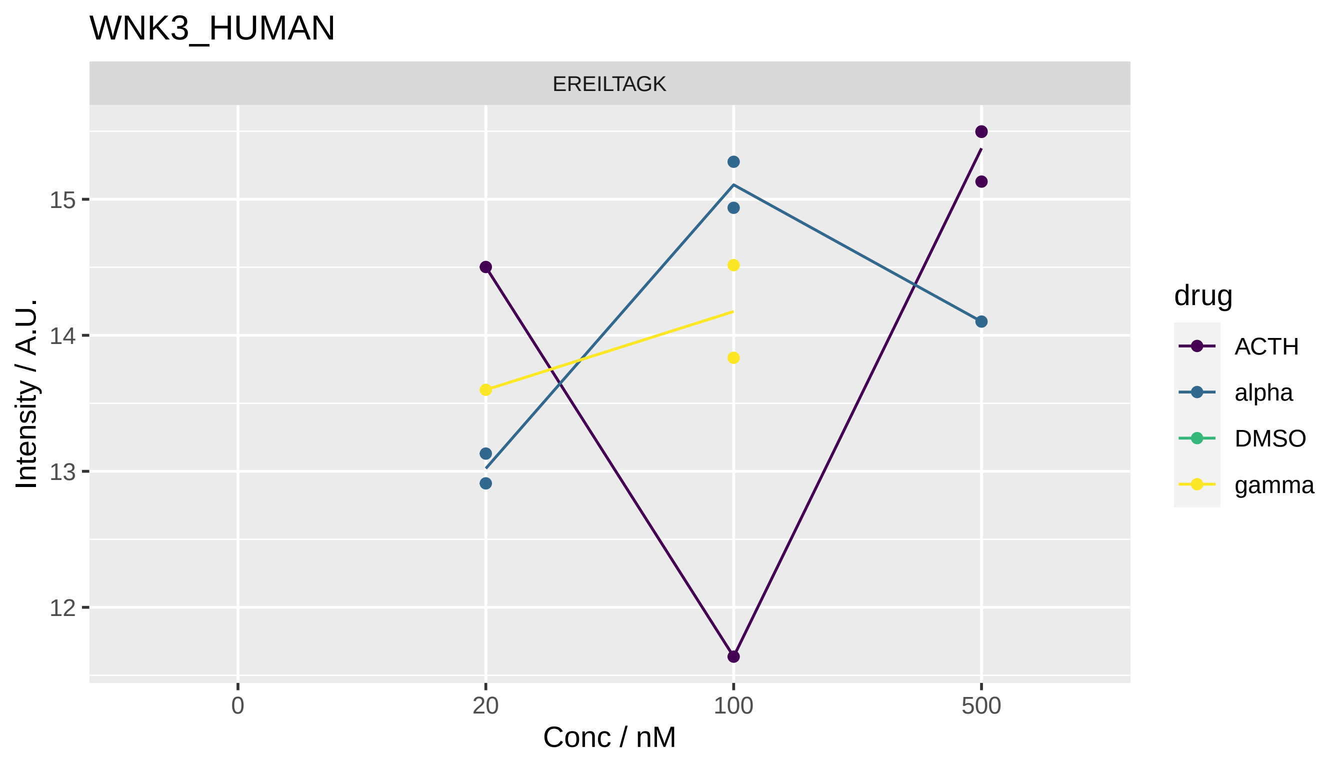

WNK3_HUMAN
EREILTAGK
Intensity / A.U.
15
14
13
12
0
20
100
500
Conc / nM
drug
ACTH
alpha
DMSO
gamma

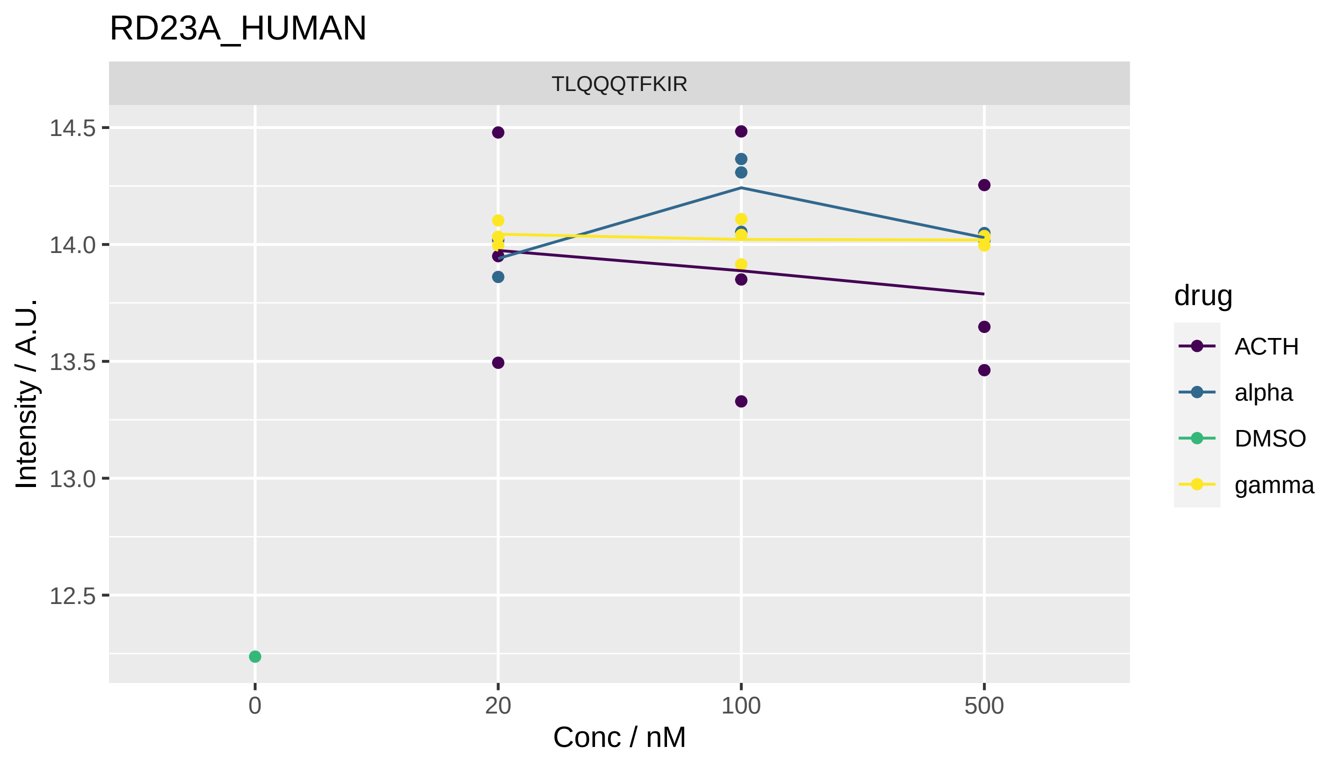

RD23A_HUMAN
TLQQQTFKIR
14.5
14.0
13.5
13.0
12.5
Intensity / A.U.
0
20
100
500
Conc / nM
drug
ACTH
alpha
DMSO
gamma

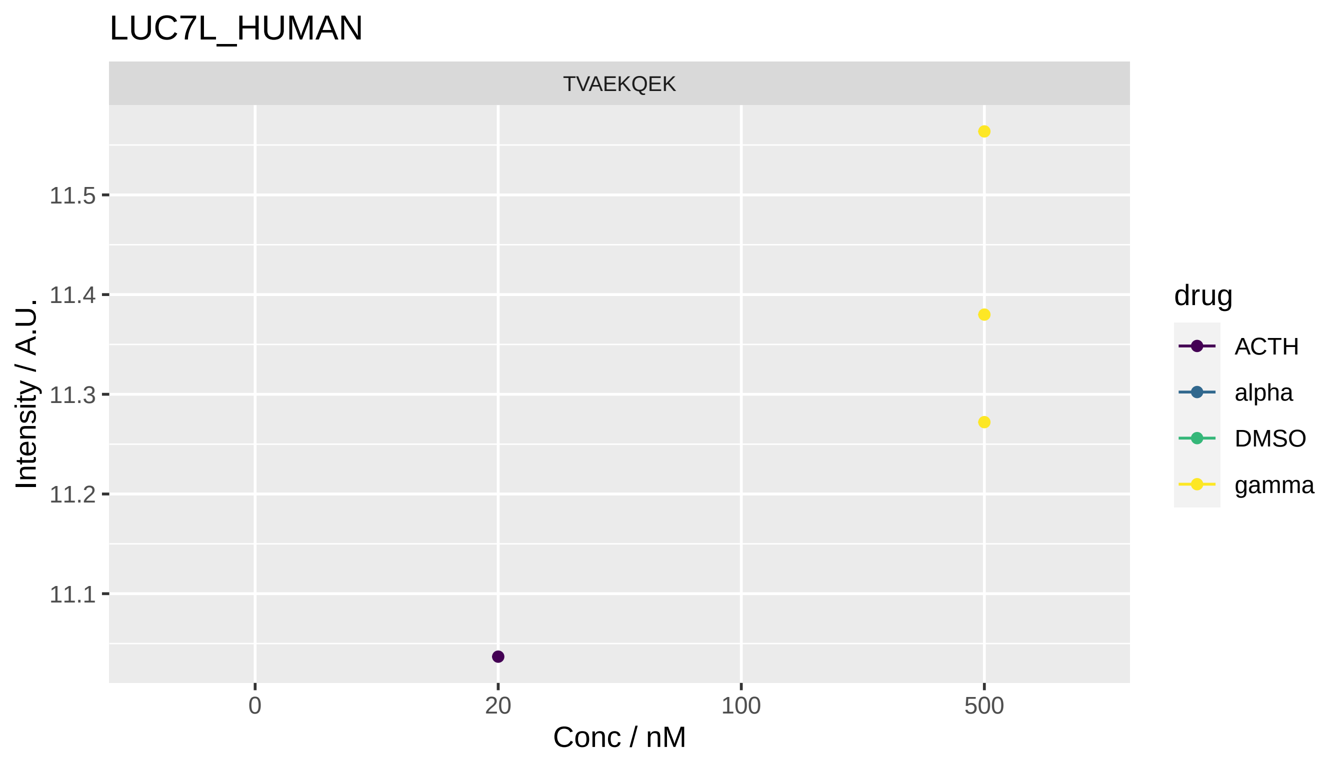
LUC7L_HUMAN
TVAEKQEK
Intensity / A.U.
11.5
11.4
11.3
11.2
11.1
0
20
100
500
Conc / nM
drug
ACTH
alpha
DMSO
gamma

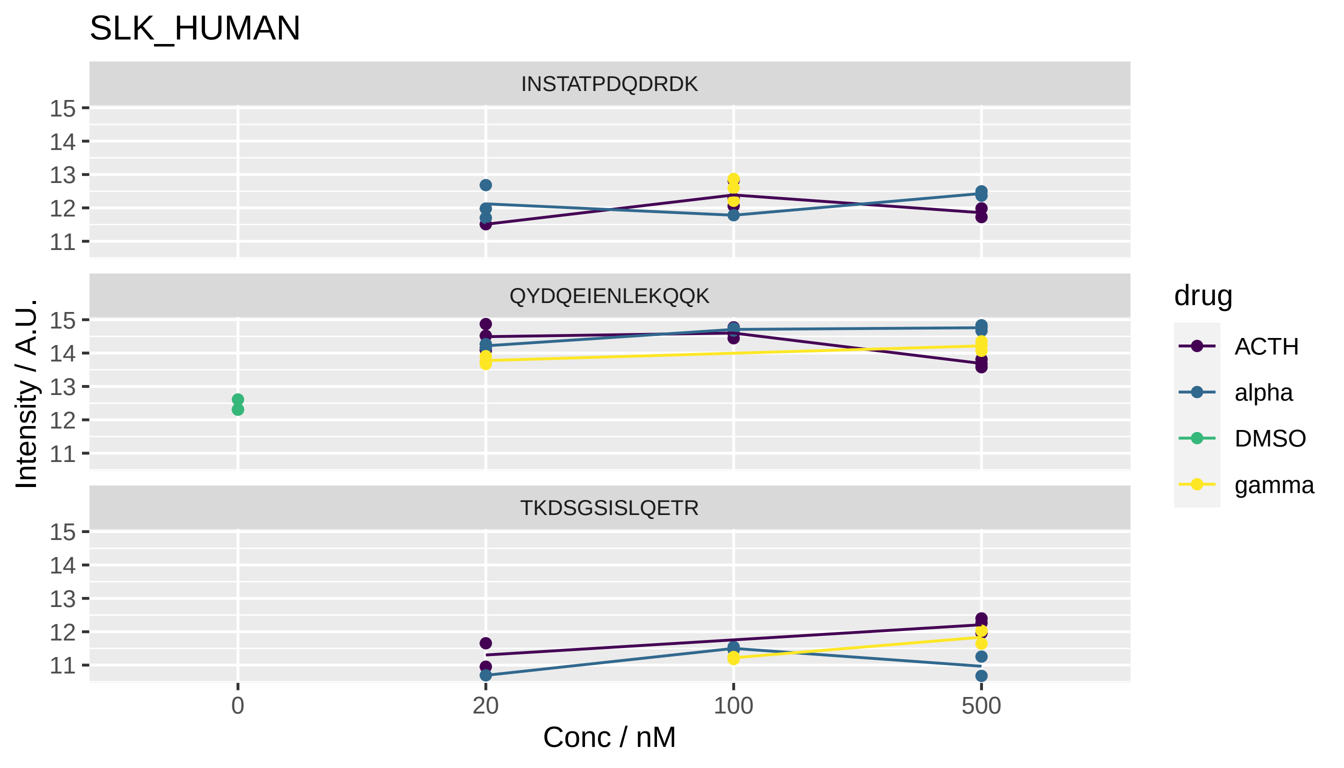
SLK_HUMAN
INSTATPDQDRDK
QYDQEIENLEKQQK
TKDSGSISLQETR
Intensity / A.U.
11
12
13
14
15
Conc / nM
0
20
100
500
drug
ACTH
alpha
DMSO
gamma

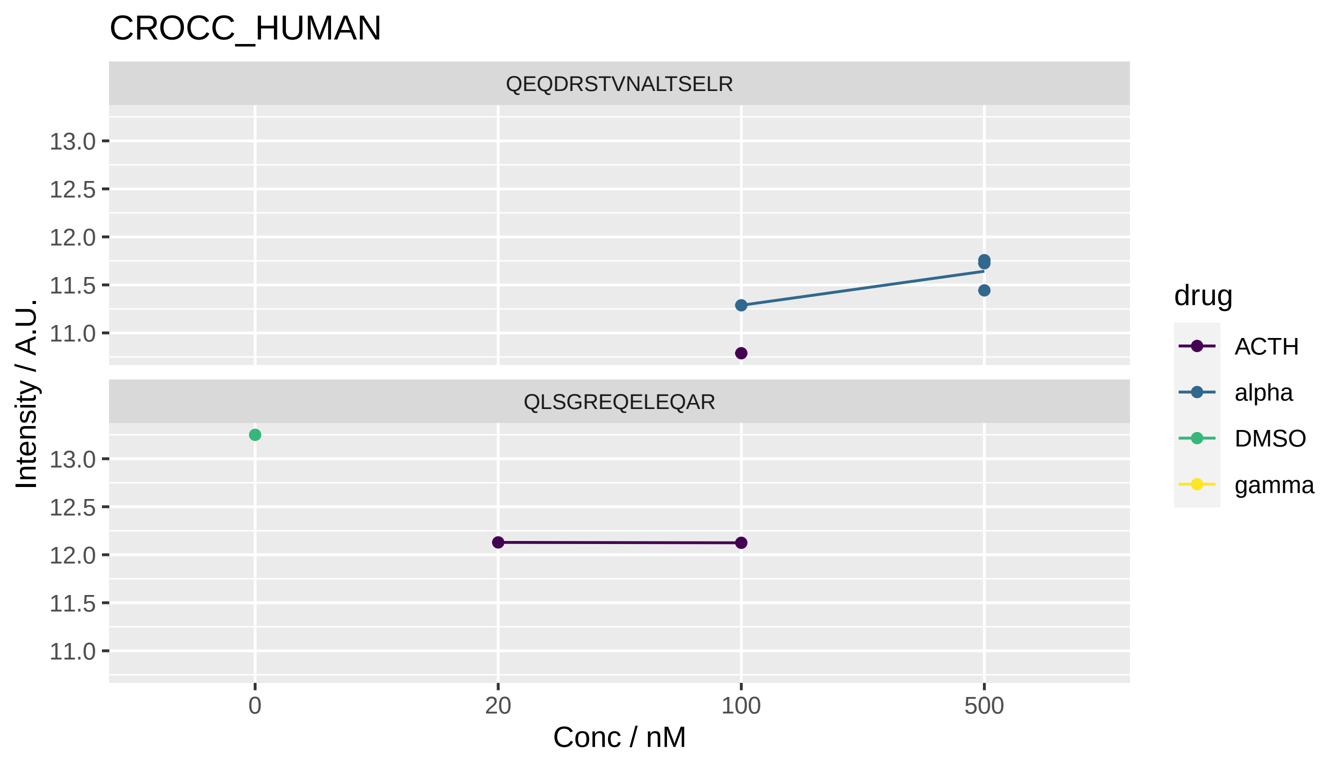
CROCC_HUMAN
QEQDRSTVNALTSELR
QLSGREQELEQAR
13.0
12.5
12.0
11.5
11.0
Intensity / A.U.
0
20
100
500
Conc / nM
drug
ACTH
alpha
DMSO
gamma

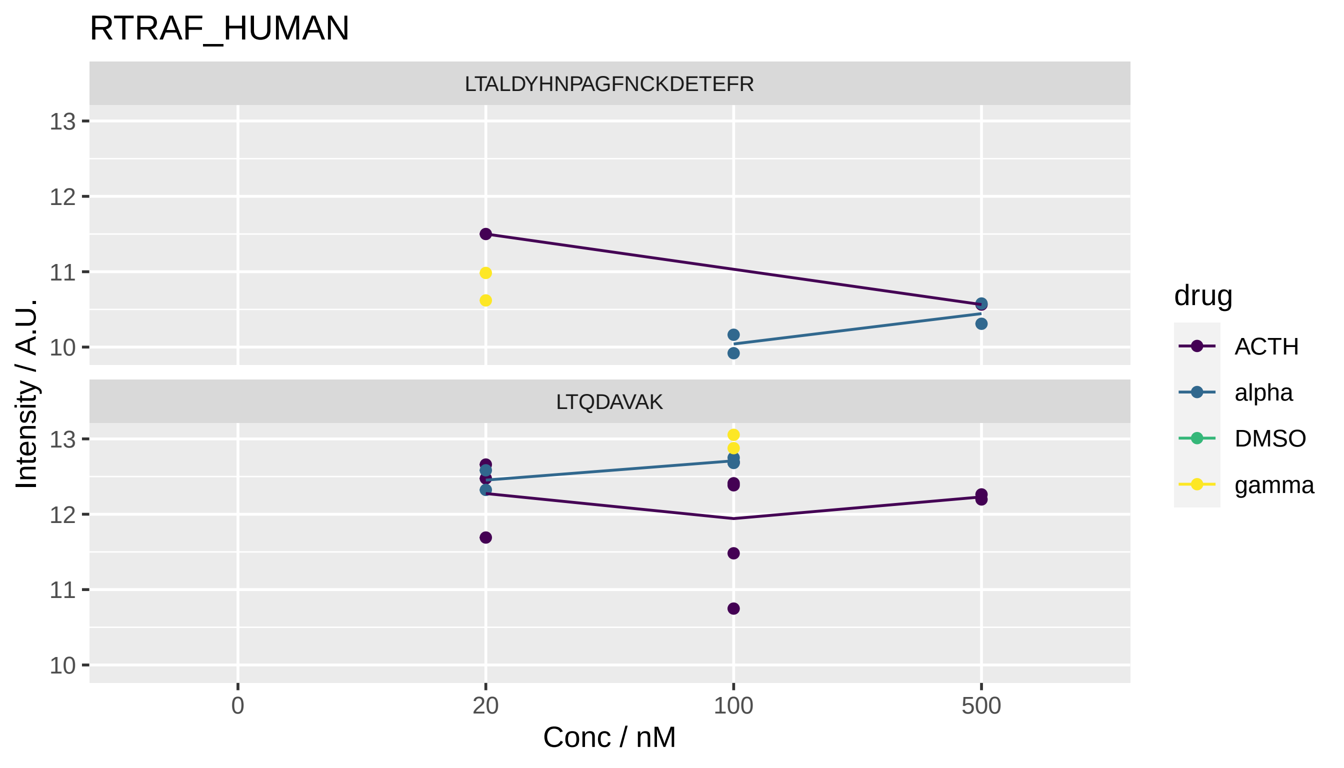
RTRAF_HUMAN
LTALDYHNPAGFNCKDETEFR
LTQDAVAK
Intensity / A.U.
Conc / nM
0
20
100
500
10
11
12
13
drug
ACTH
alpha
DMSO
gamma

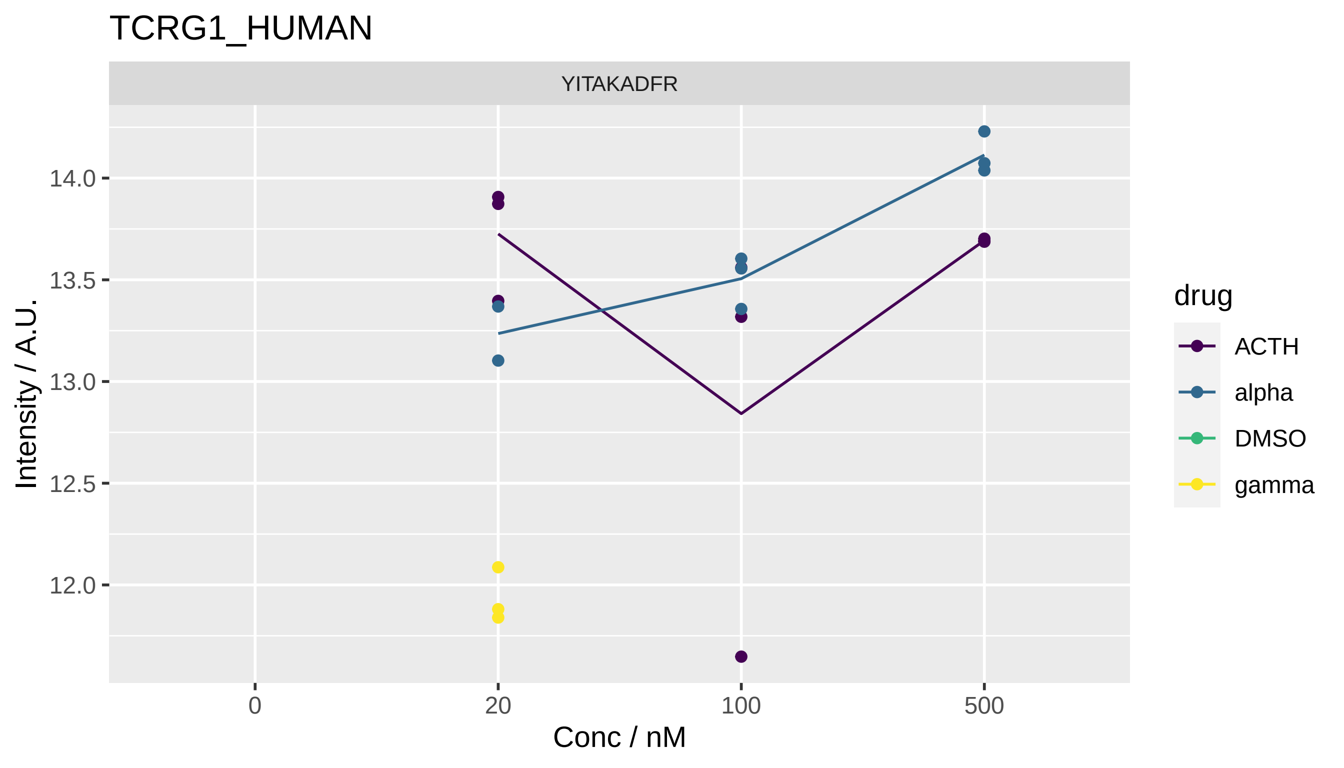
TCRG1_HUMAN
YITAKADFR
Intensity / A.U.
14.0
13.5
13.0
12.5
12.0
0
20
100
500
Conc / nM
drug
ACTH
alpha
DMSO
gamma

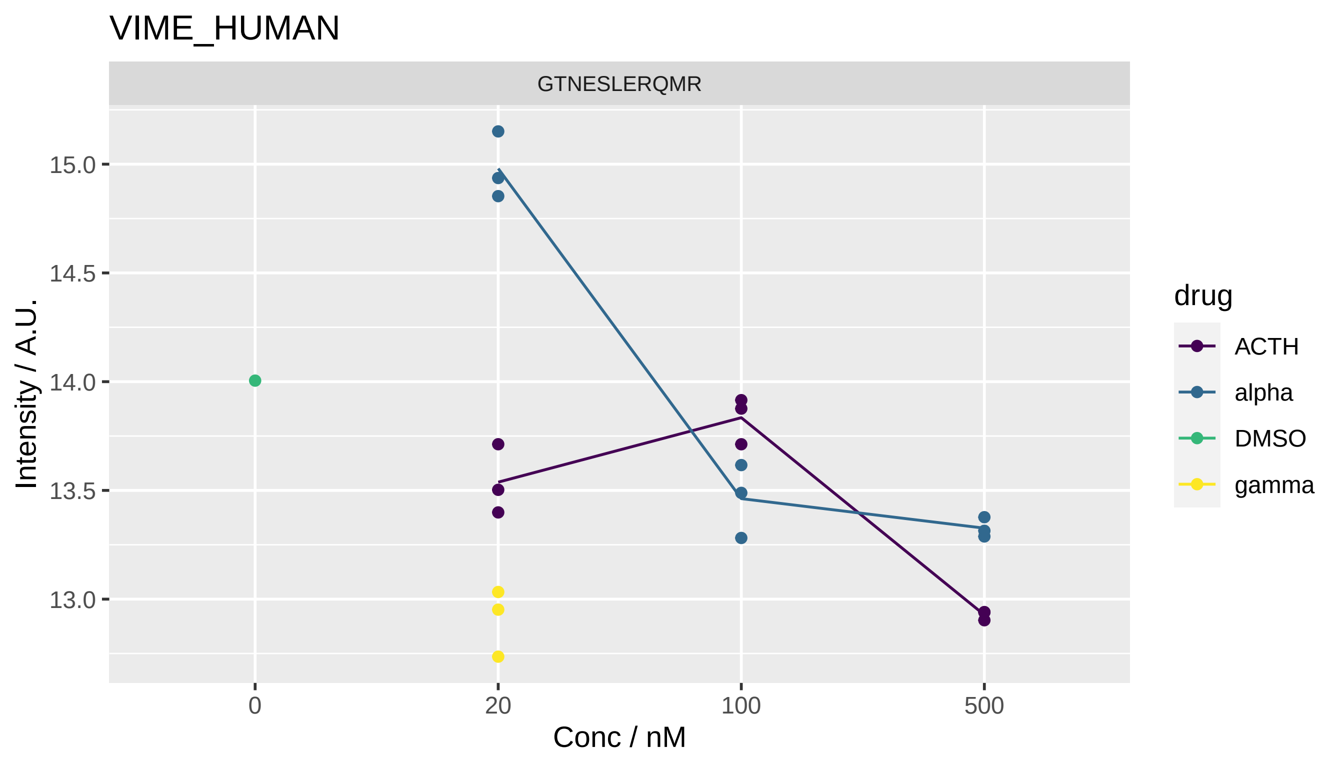
VIME_HUMAN
GTNESLERQMR
Intensity / A.U.
15.0
14.5
14.0
13.5
13.0
0
20
100
500
Conc / nM
drug
ACTH
alpha
DMSO
gamma

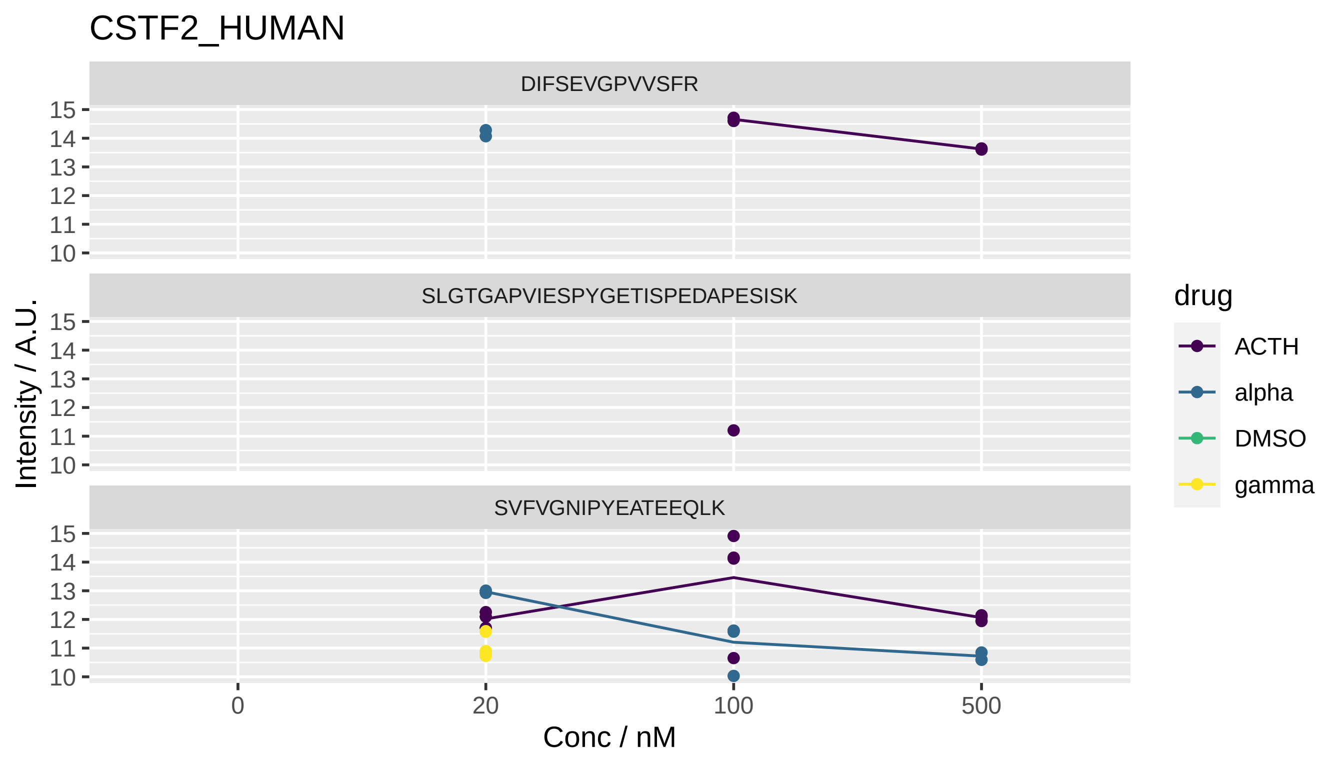
CSTF2_HUMAN
DIFSEVGPVVSFR
SLGTGAPVIESPYGETISPEDAPESISK
SVFVGNIPYEATEEQLK
Intensity / A.U.
15
14
13
12
11
10
Conc / nM
0
20
100
500
drug
ACTH
alpha
DMSO
gamma

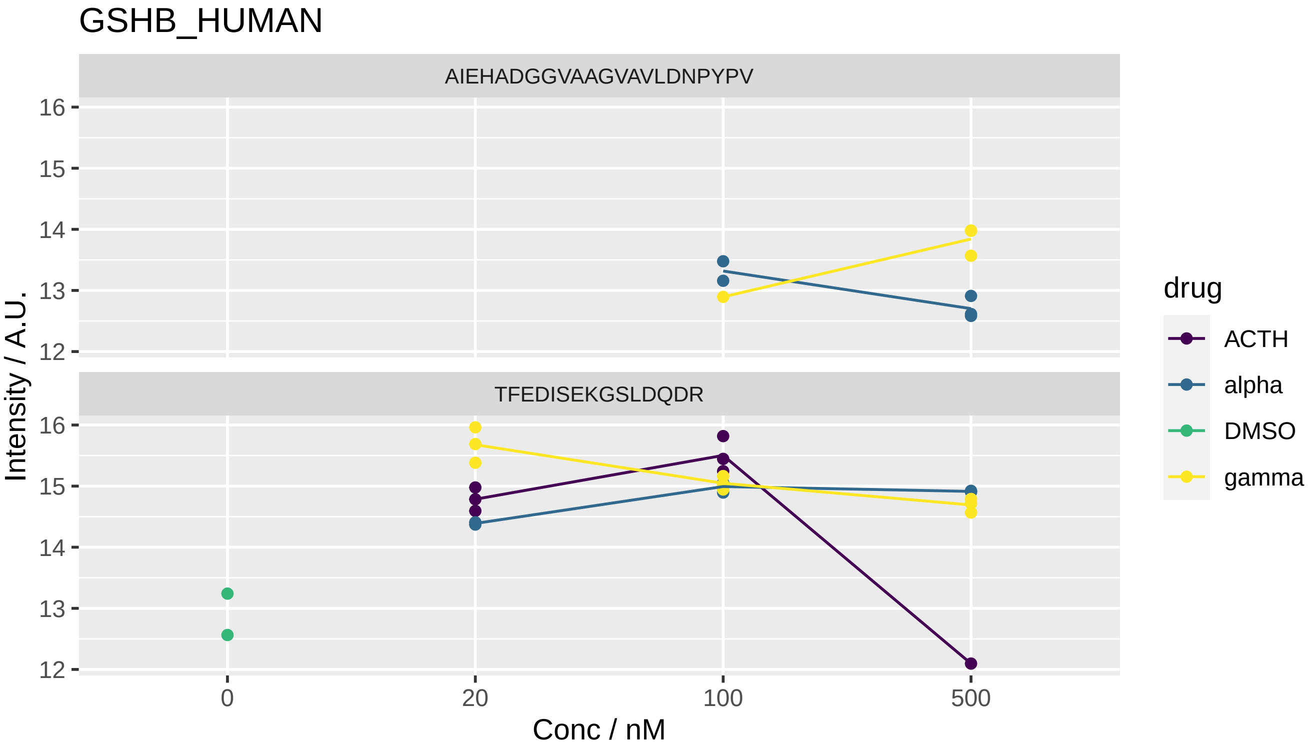

GSHB_HUMAN
AIEHADGGVAAGVAVLDNPYPV
TFEDISEKGSLDQDR
Intensity / A.U.
16
15
14
13
12
0
20
100
500
Conc / nM
drug
ACTH
alpha
DMSO
gamma

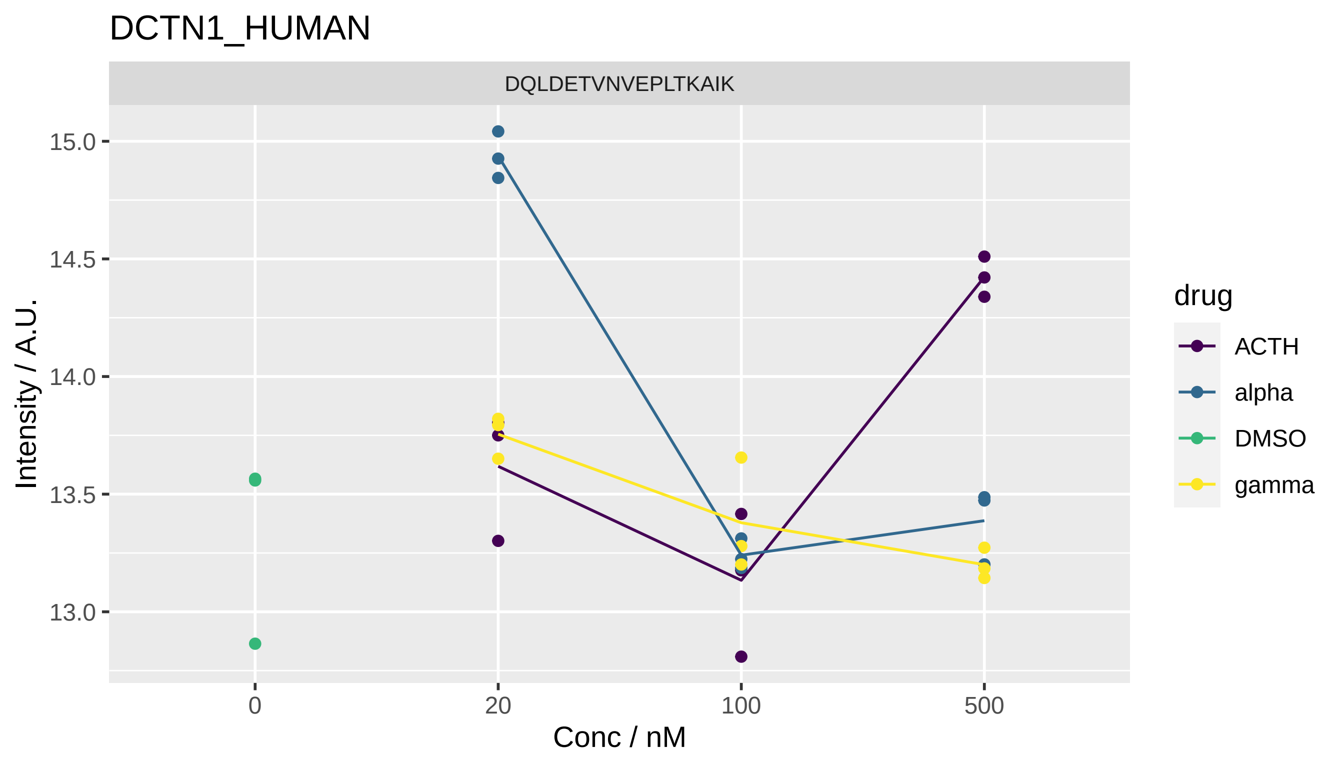
DCTN1_HUMAN
DQLDETVNVEPLTKAIK
Intensity / A.U.
15.0
14.5
14.0
13.5
13.0
0
20
100
500
Conc / nM
drug
ACTH
alpha
DMSO
gamma

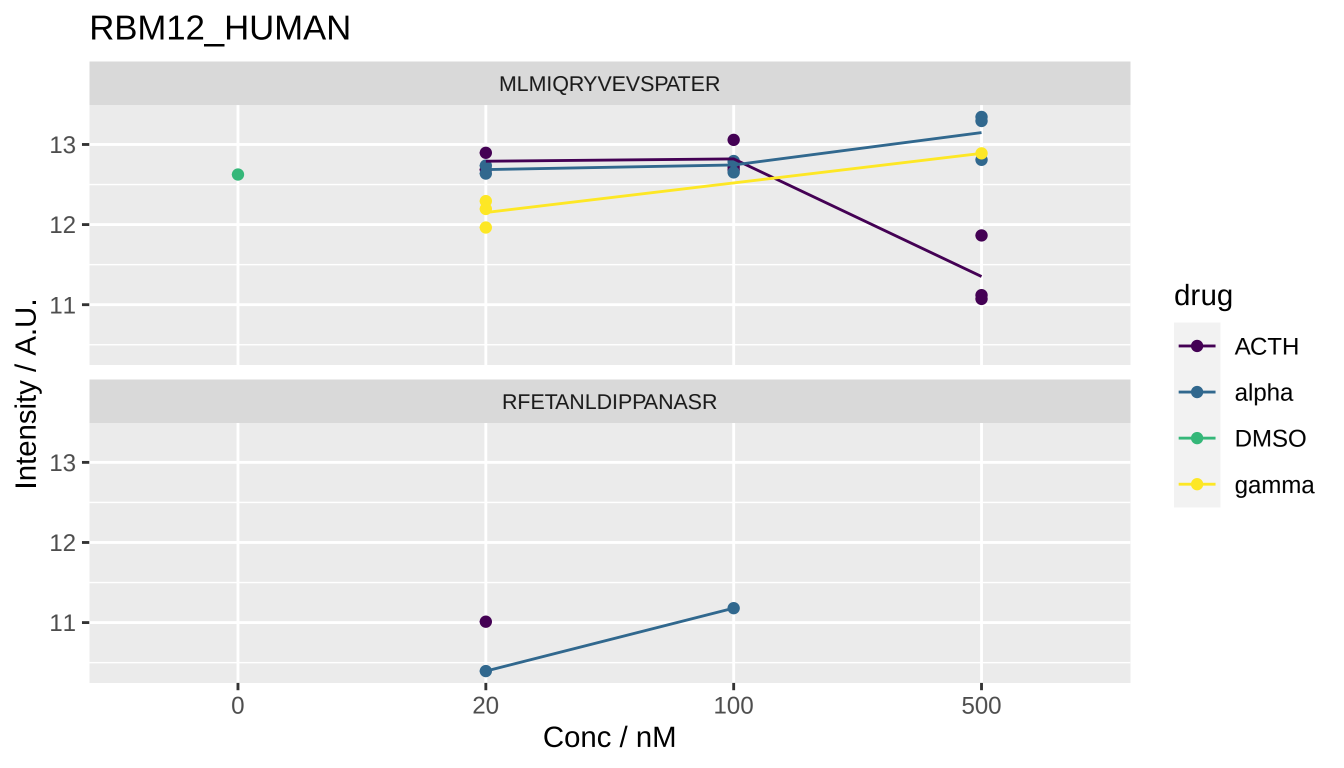

RBM12_HUMAN
MLMIQRYVEVSPATER
RFETANLDIPPANASR
13
12
11
13
12
11
0
20
100
500
Conc / nM
Intensity / A.U.
drug
ACTH
alpha
DMSO
gamma

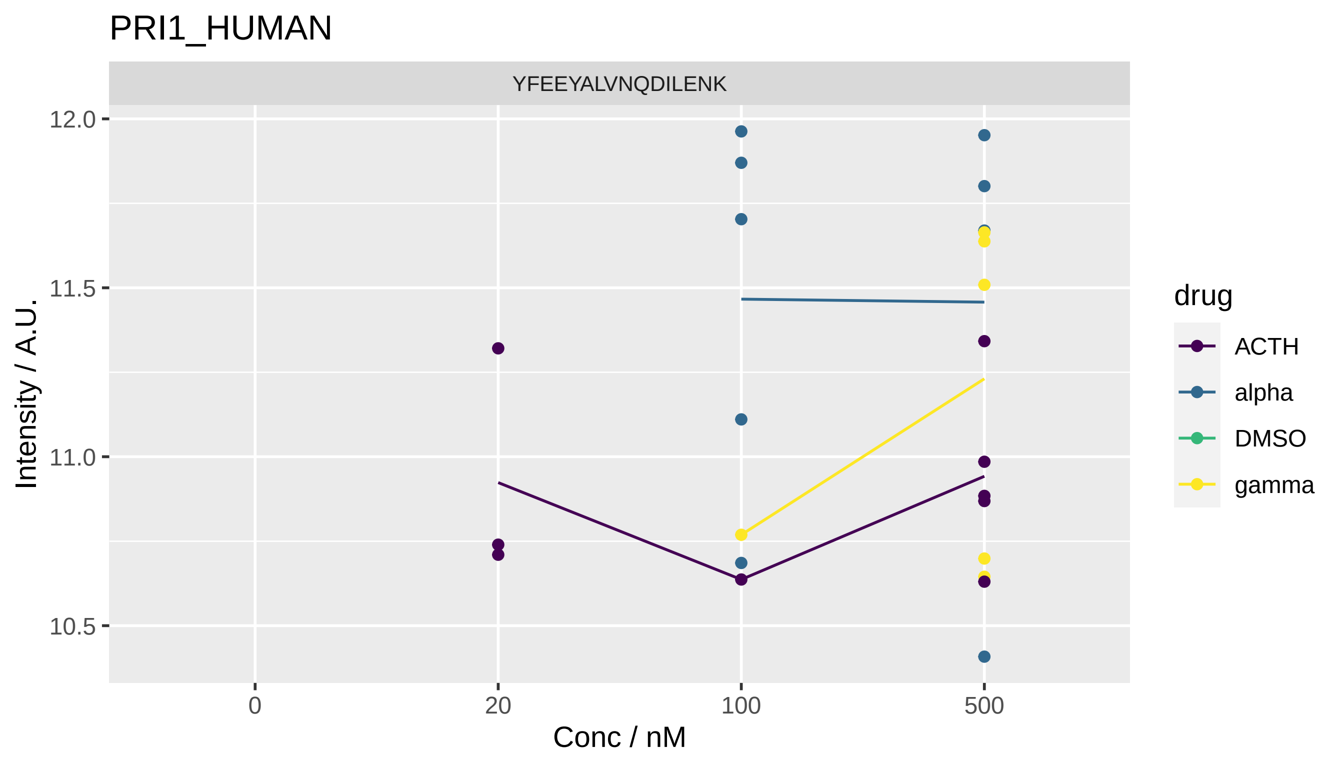
PRI1_HUMAN
YFEEYALVNQDILENK
12.0
11.5
11.0
10.5
Intensity / A.U.
0
20
100
500
Conc / nM
drug
ACTH
alpha
DMSO
gamma

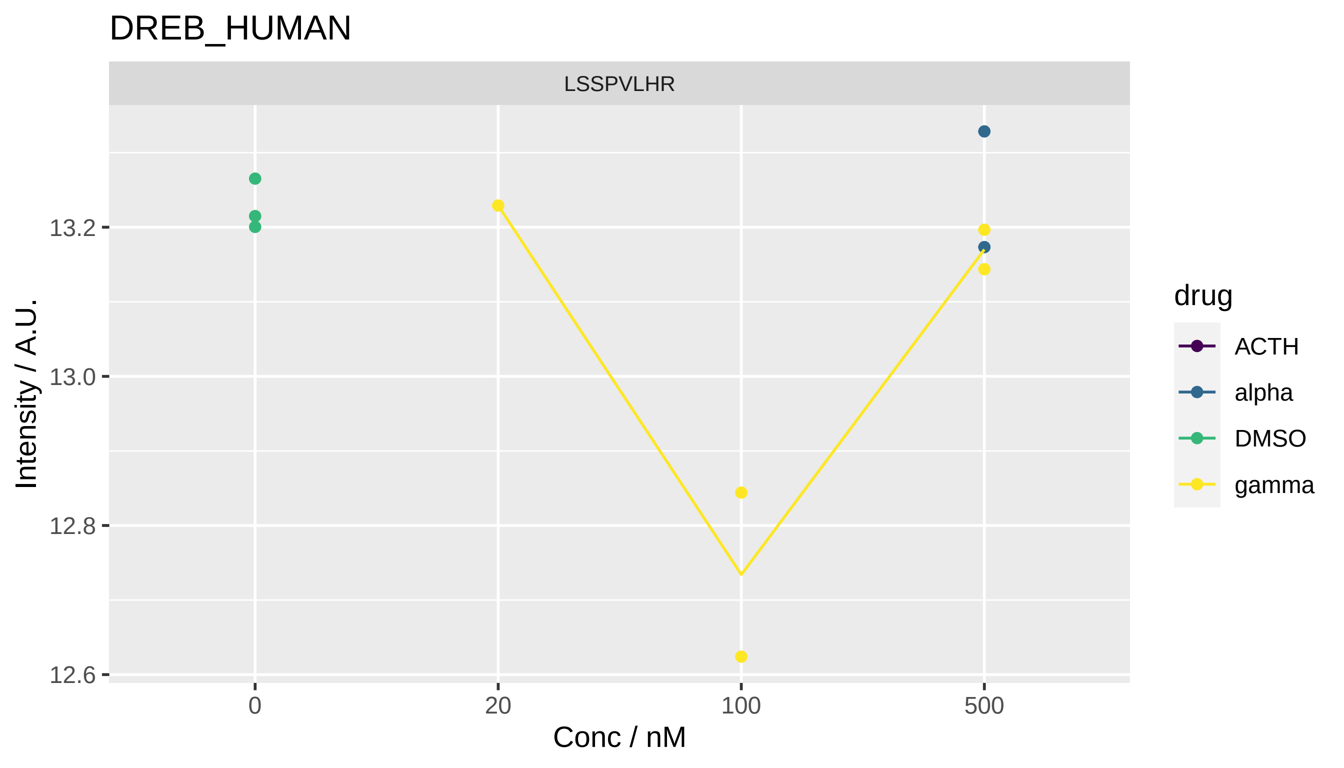
DREB_HUMAN
LSSPVLHR
Intensity / A.U.
13.2
13.0
12.8
12.6
0
20
100
500
Conc / nM
drug
ACTH
alpha
DMSO
gamma

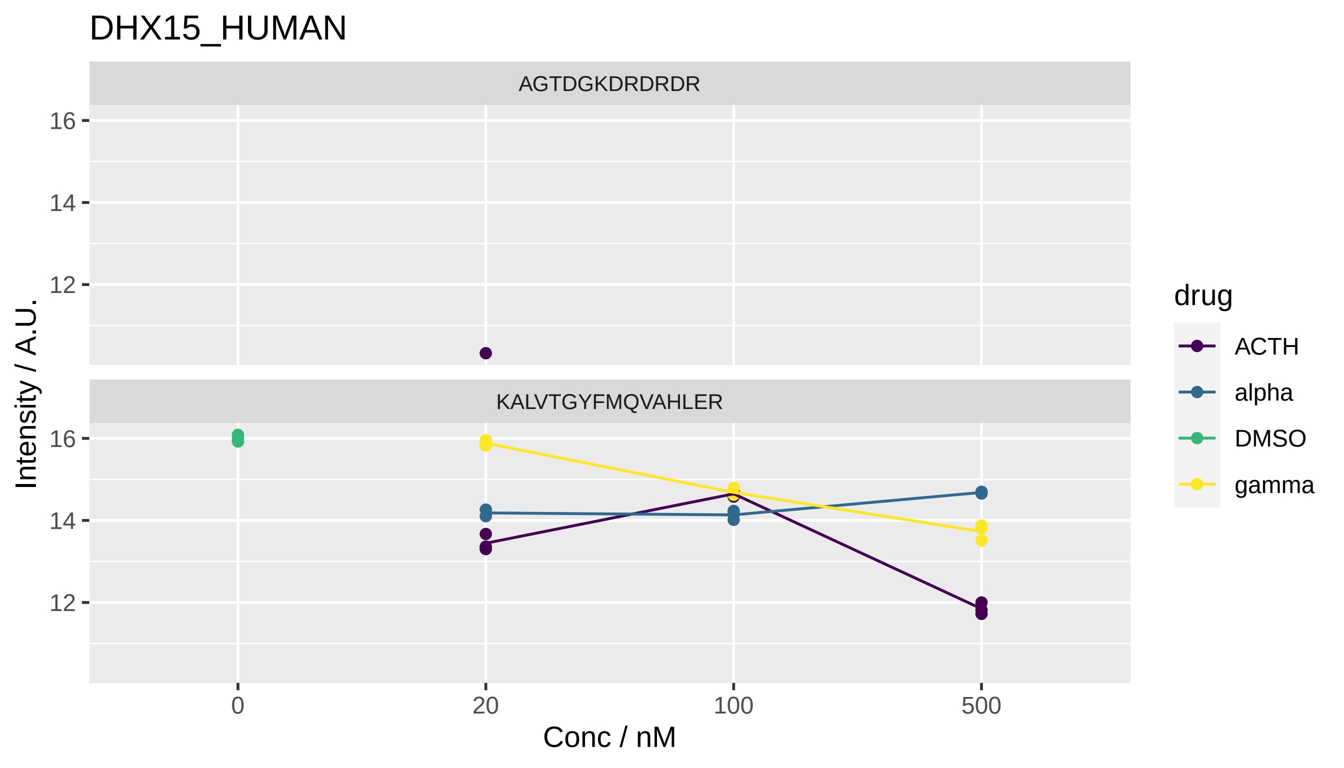

DHX15_HUMAN
AGTDGKDRDRDR
KALVTGYFMQVAHLER
Intensity / A.U.
16
14
12
0
20
100
500
Conc / nM
drug
ACTH
alpha
DMSO
gamma

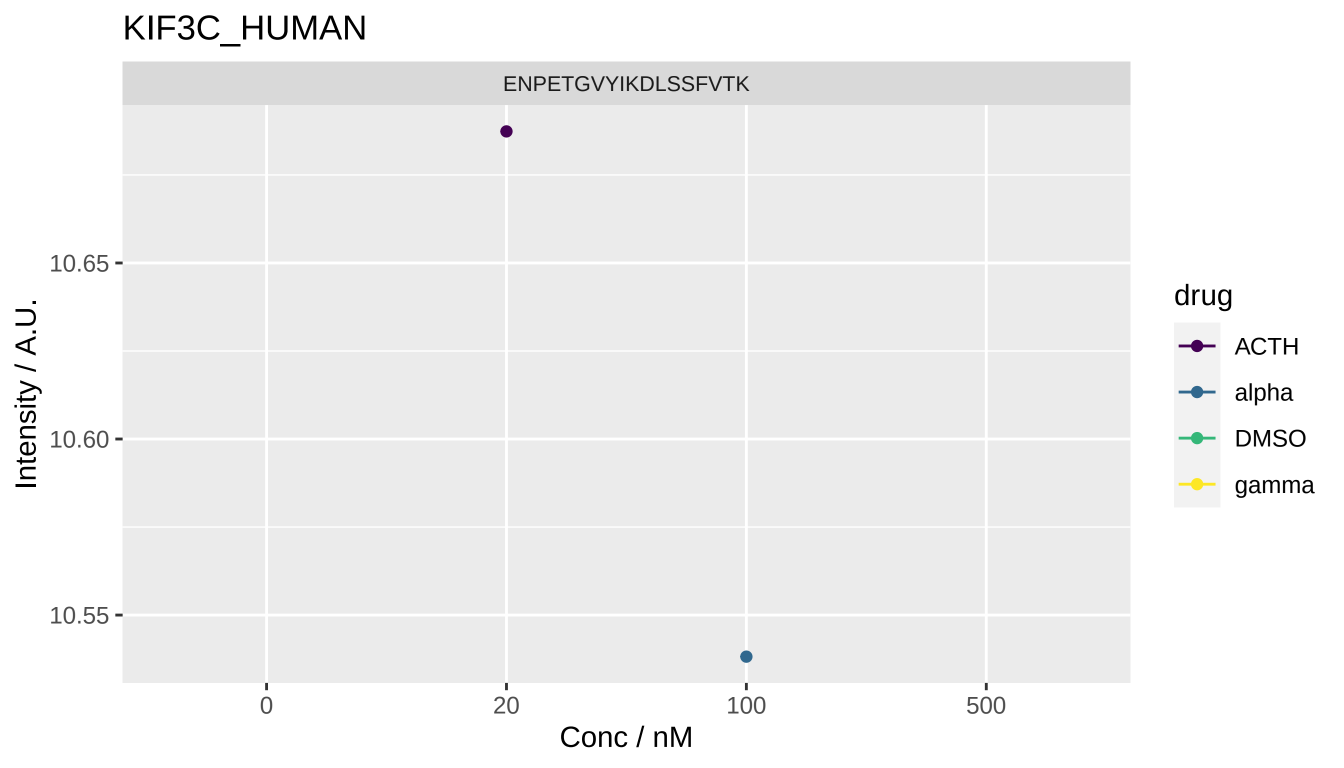
KIF3C_HUMAN
ENPETGVYIKDLSSFVTK
Intensity / A.U.
10.65
10.60
10.55
0
20
100
500
Conc / nM
drug
ACTH
alpha
DMSO
gamma

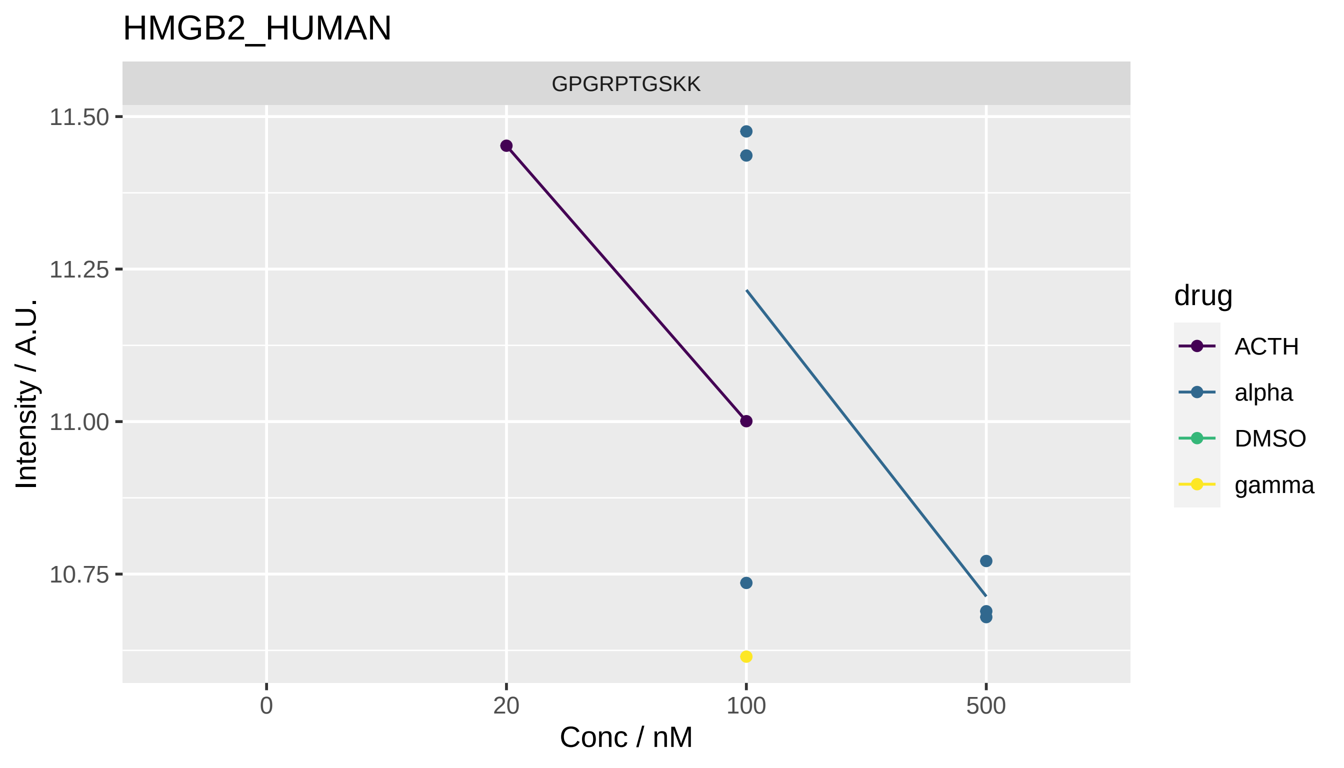

HMGB2_HUMAN
GPGRPTGSKK
Intensity / A.U.
11.50
11.25
11.00
10.75
0
20
100
500
Conc / nM
drug
ACTH
alpha
DMSO
gamma

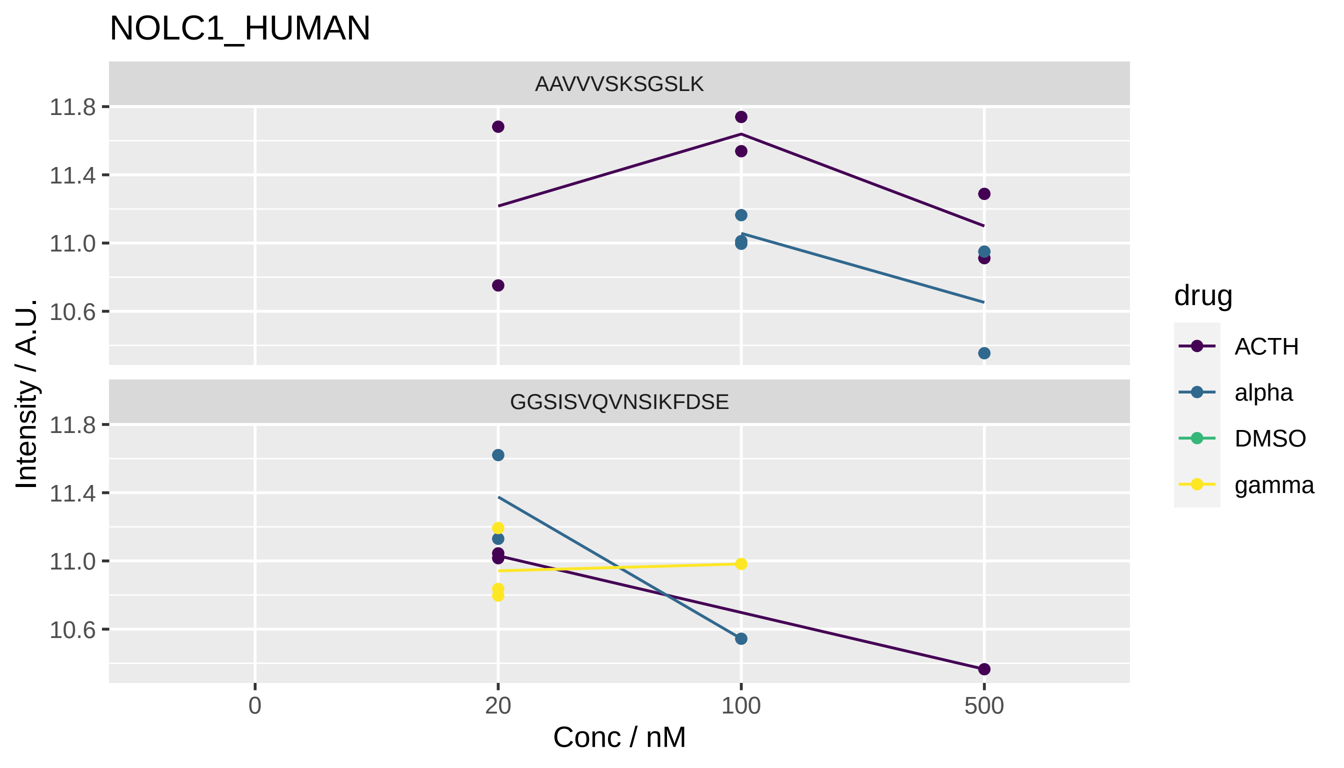

NOLC1_HUMAN
AAVVVSKSGSLK
GGSISVQVNSIKFDSE
11.8
11.4
11.0
10.6
Intensity / A.U.
0
20
100
500
Conc / nM
drug
ACTH
alpha
DMSO
gamma

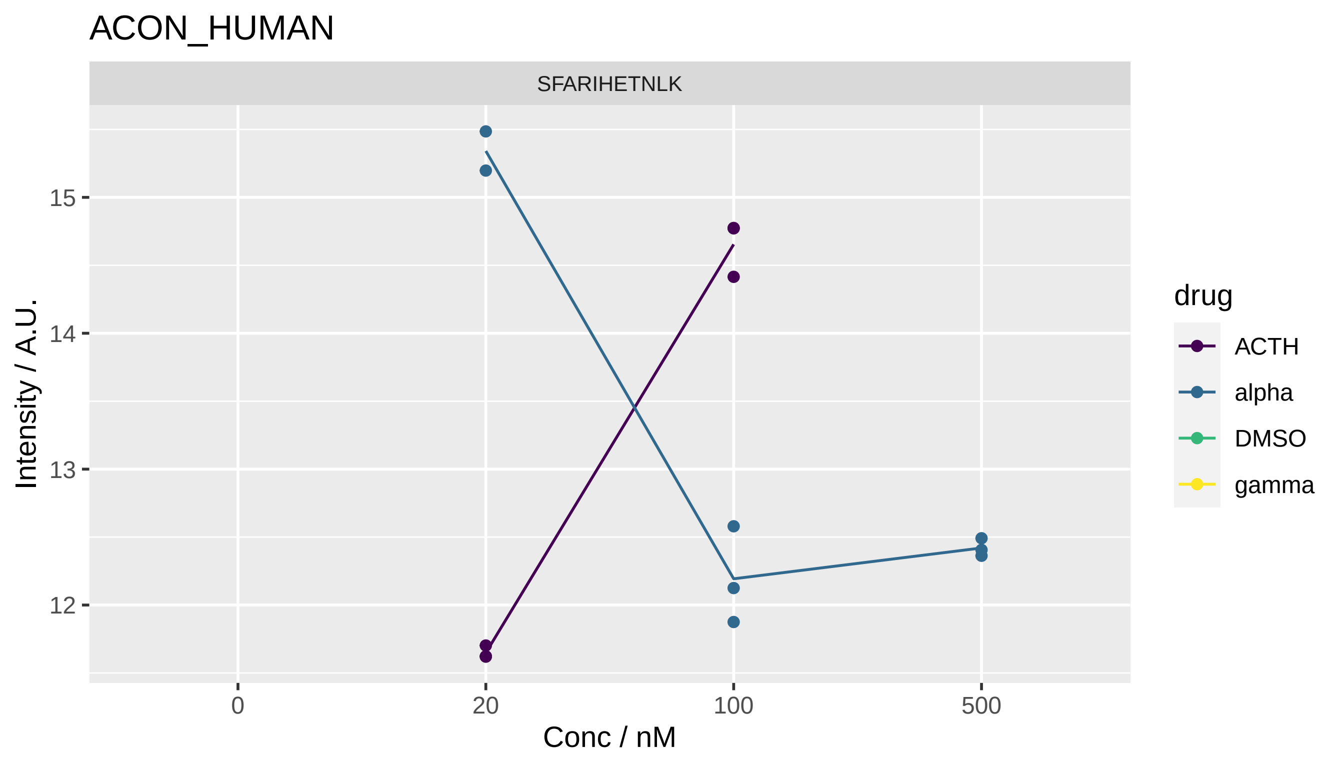

ACON_HUMAN
SFARIHETNLK
Intensity / A.U.
15
14
13
12
0
20
100
500
Conc / nM
drug
ACTH
alpha
DMSO
gamma

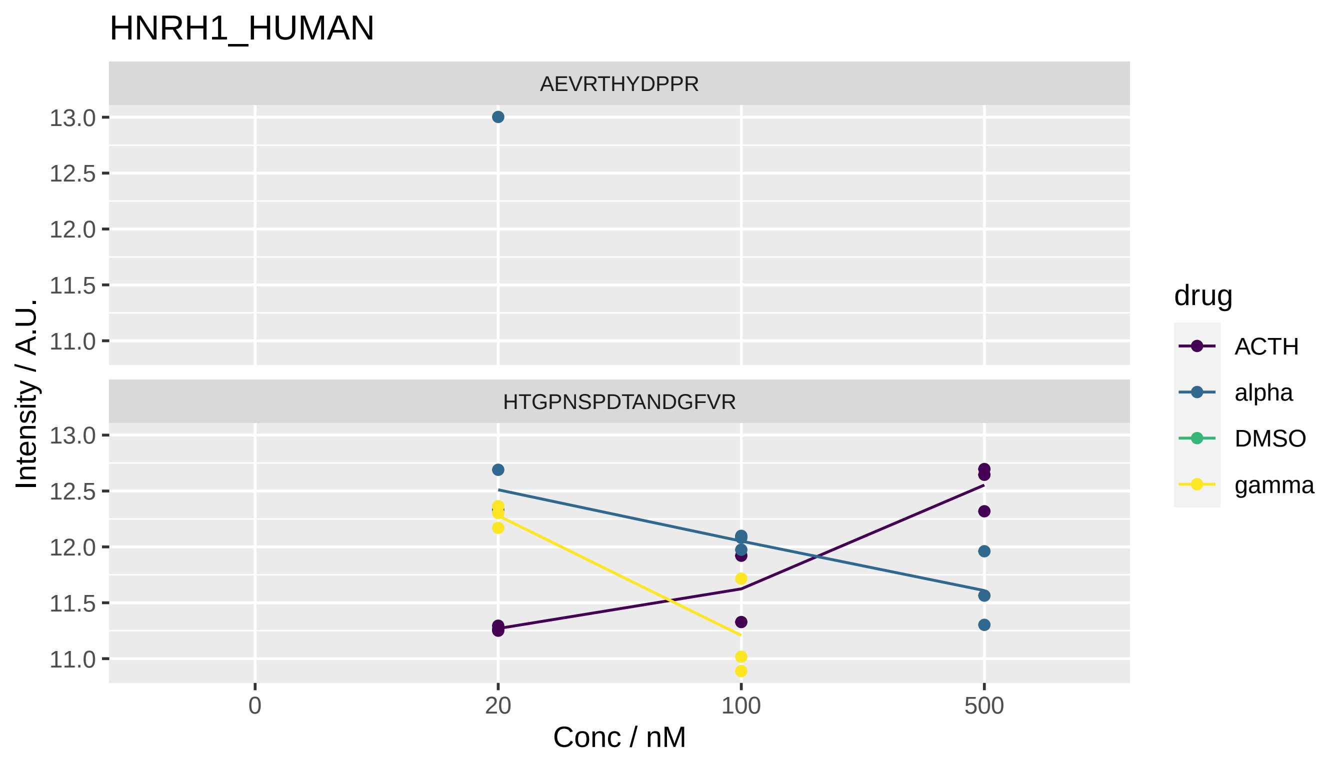
HNRH1_HUMAN
AEVRTHYDPPR
HTGPNSPDTANDGFVR
Intensity / A.U.
13.0
12.5
12.0
11.5
11.0
0
20
100
500
Conc / nM
drug
ACTH
alpha
DMSO
gamma

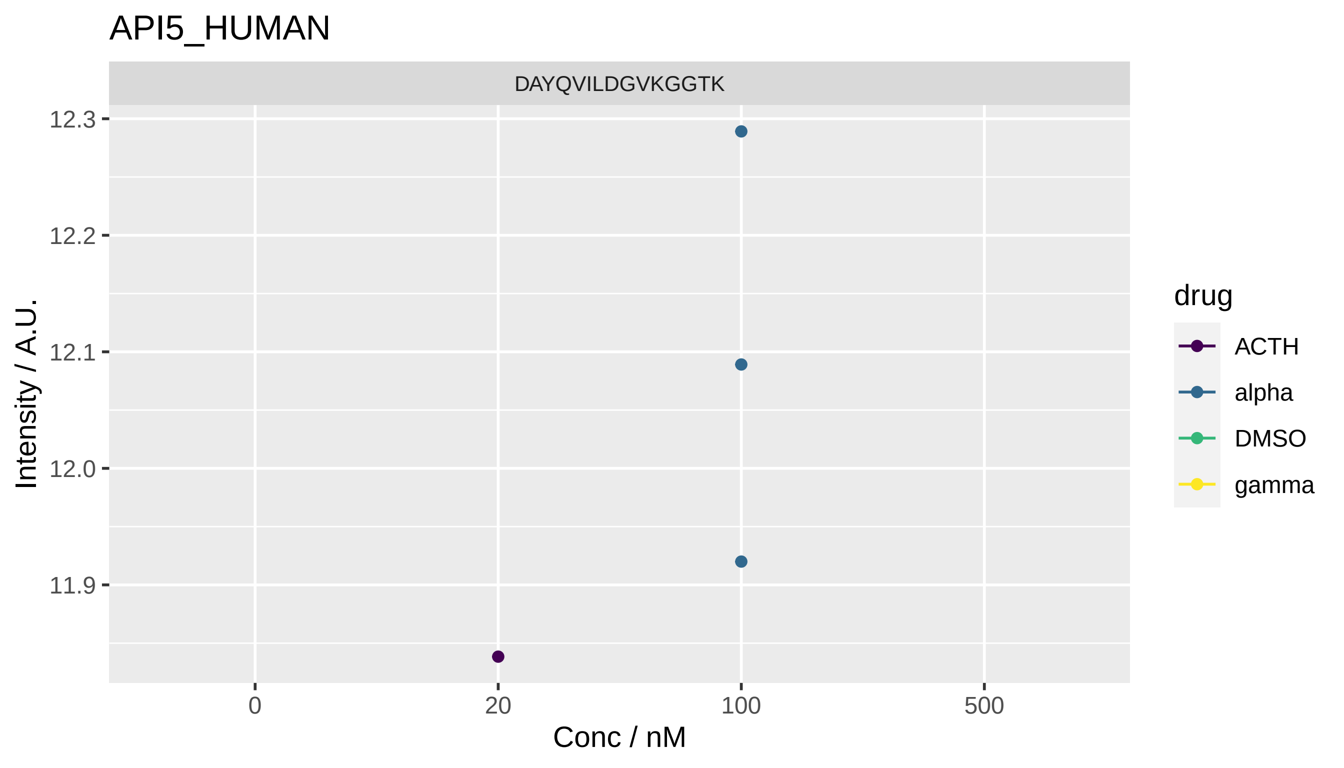
API5_HUMAN
DAYQVILDGVKGGTK
Intensity / A.U.
12.3
12.2
12.1
12.0
11.9
0
20
100
500
Conc / nM
drug
ACTH
alpha
DMSO
gamma

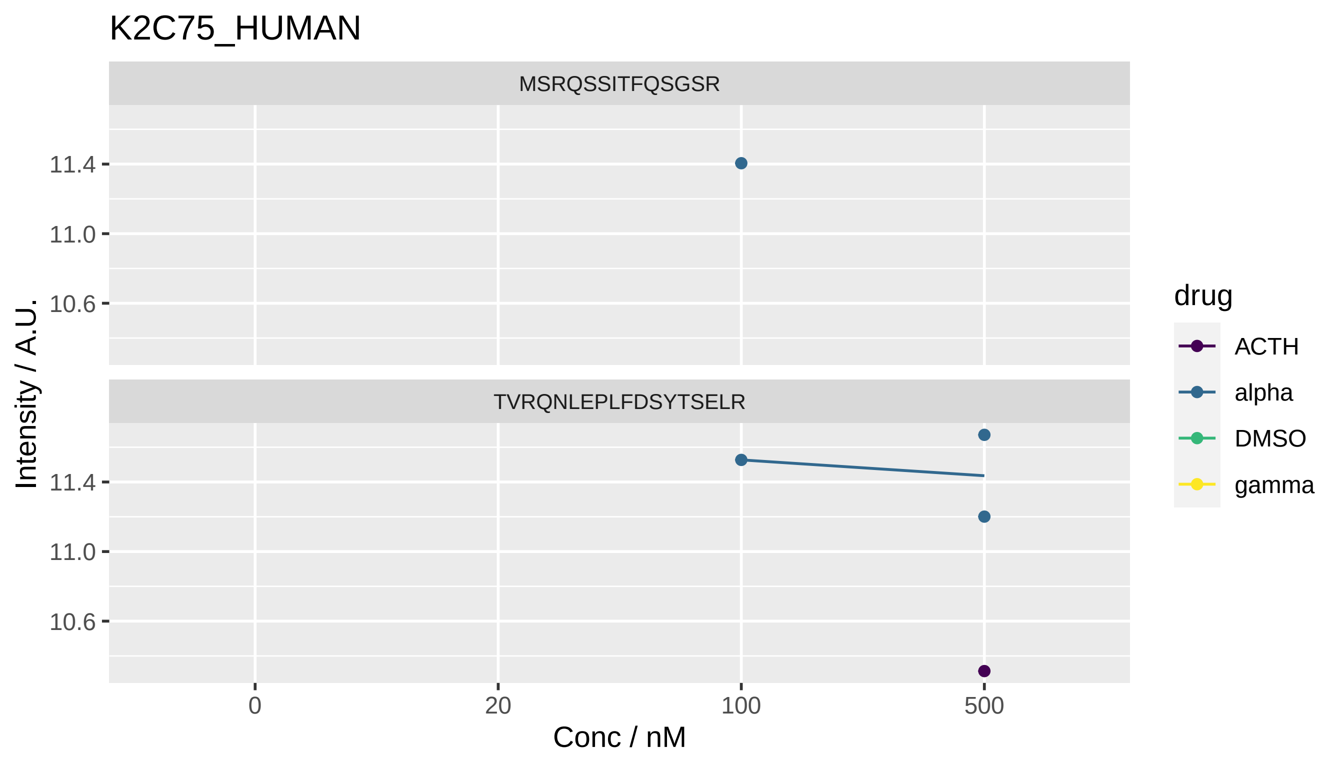
K2C75_HUMAN
MSRQSSITFQSGSR
TVRQNLEPLFDSYTSELR
11.4
11.0
10.6
Intensity / A.U.
0
20
100
500
Conc / nM
drug
ACTH
alpha
DMSO
gamma

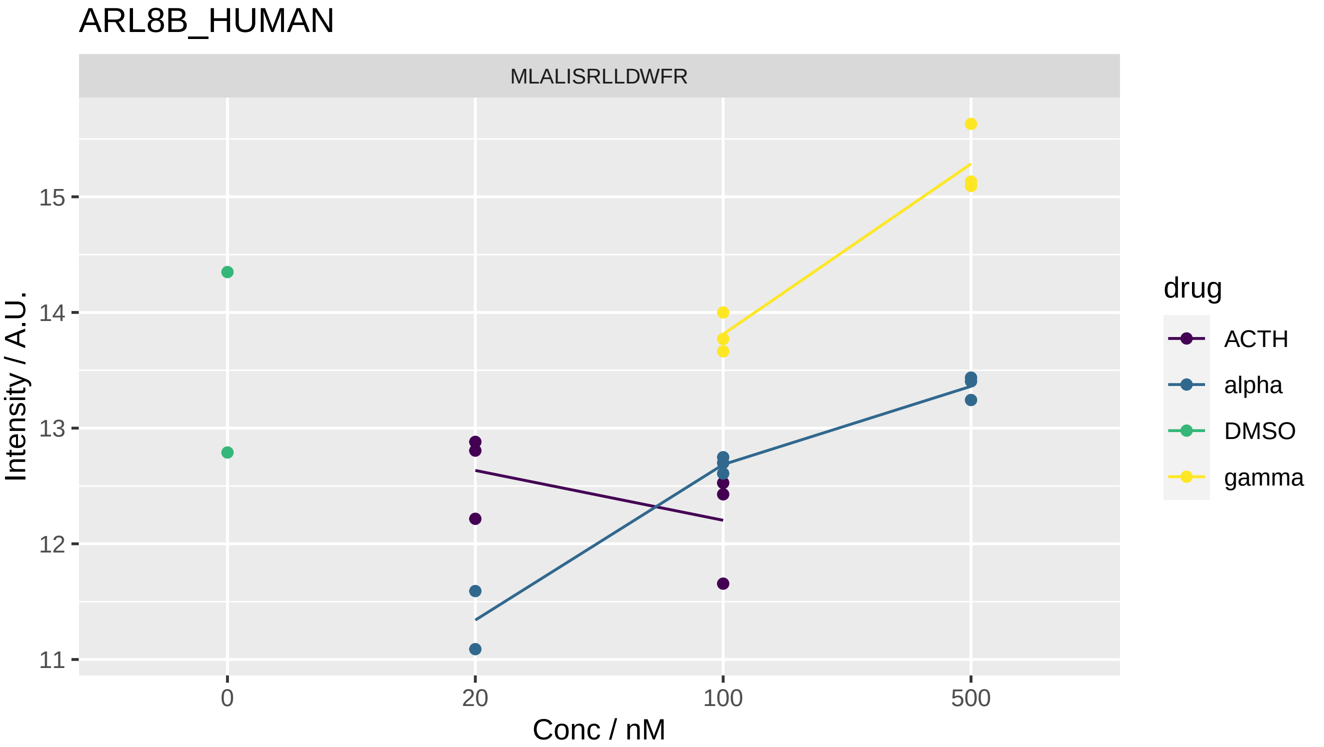
ARL8B_HUMAN
MLALISRLLDWFR
Intensity / A.U.
15
14
13
12
11
0
20
100
500
Conc / nM
drug
ACTH
alpha
DMSO
gamma

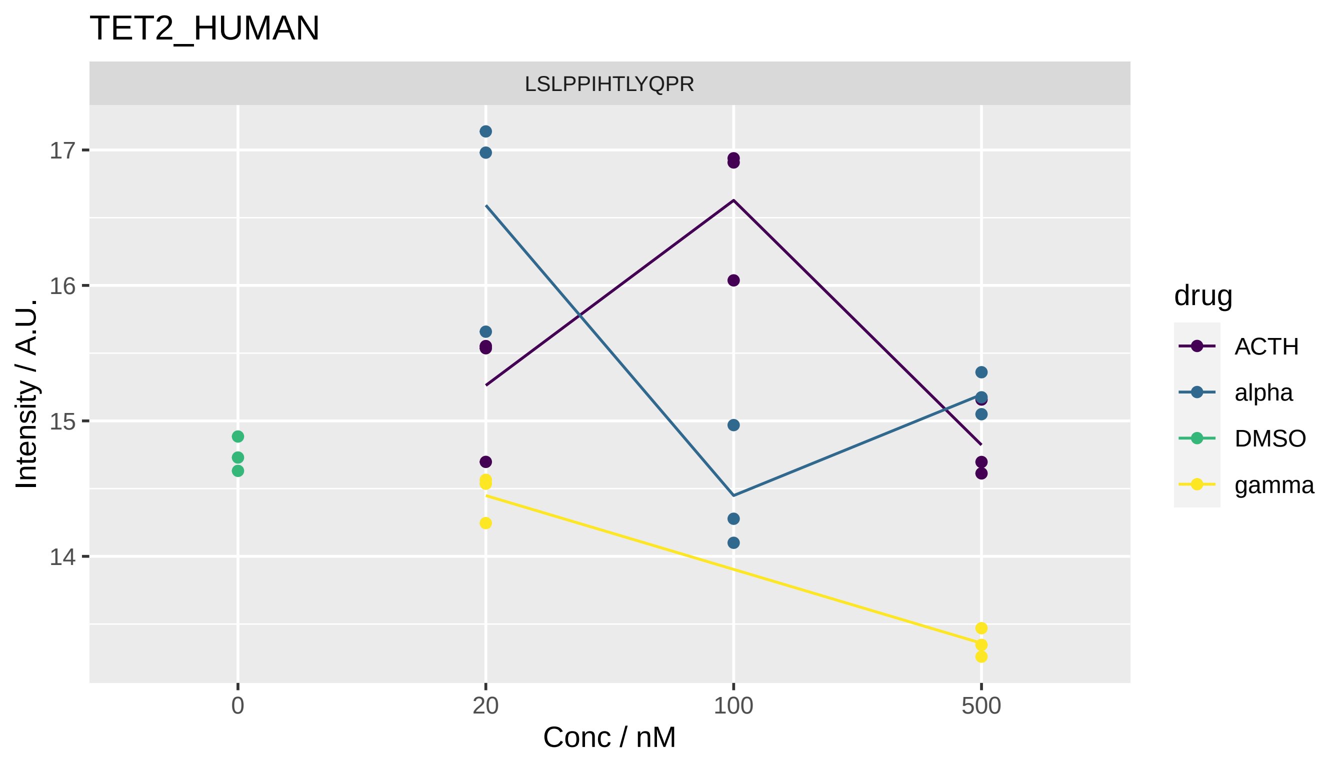
TET2_HUMAN
LSLPPIHTLYQPR
Intensity / A.U.
17
16
15
14
0
20
100
500
Conc / nM
drug
ACTH
alpha
DMSO
gamma

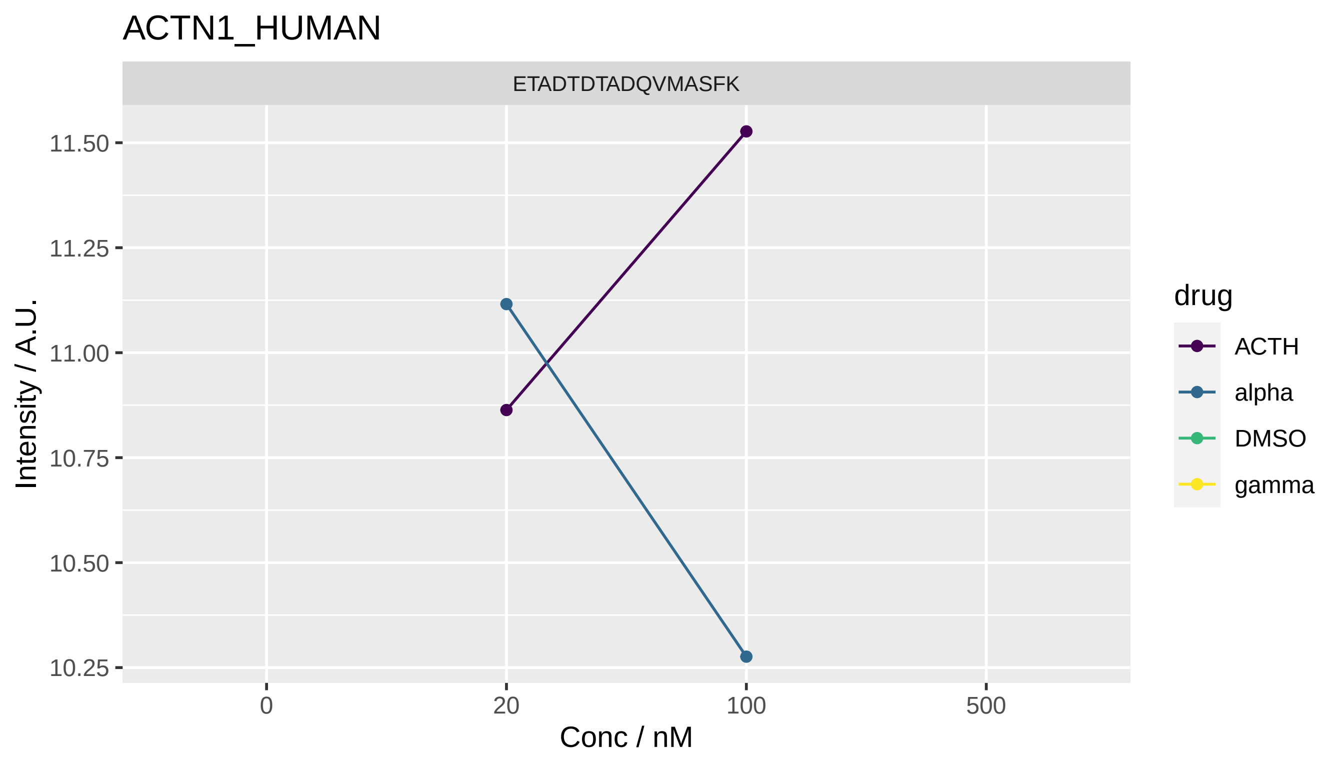
ACTN1_HUMAN
ETADTDTADQVMASFK
Intensity / A.U.
11.50
11.25
11.00
10.75
10.50
10.25
0
20
100
500
Conc / nM
drug
ACTH
alpha
DMSO
gamma

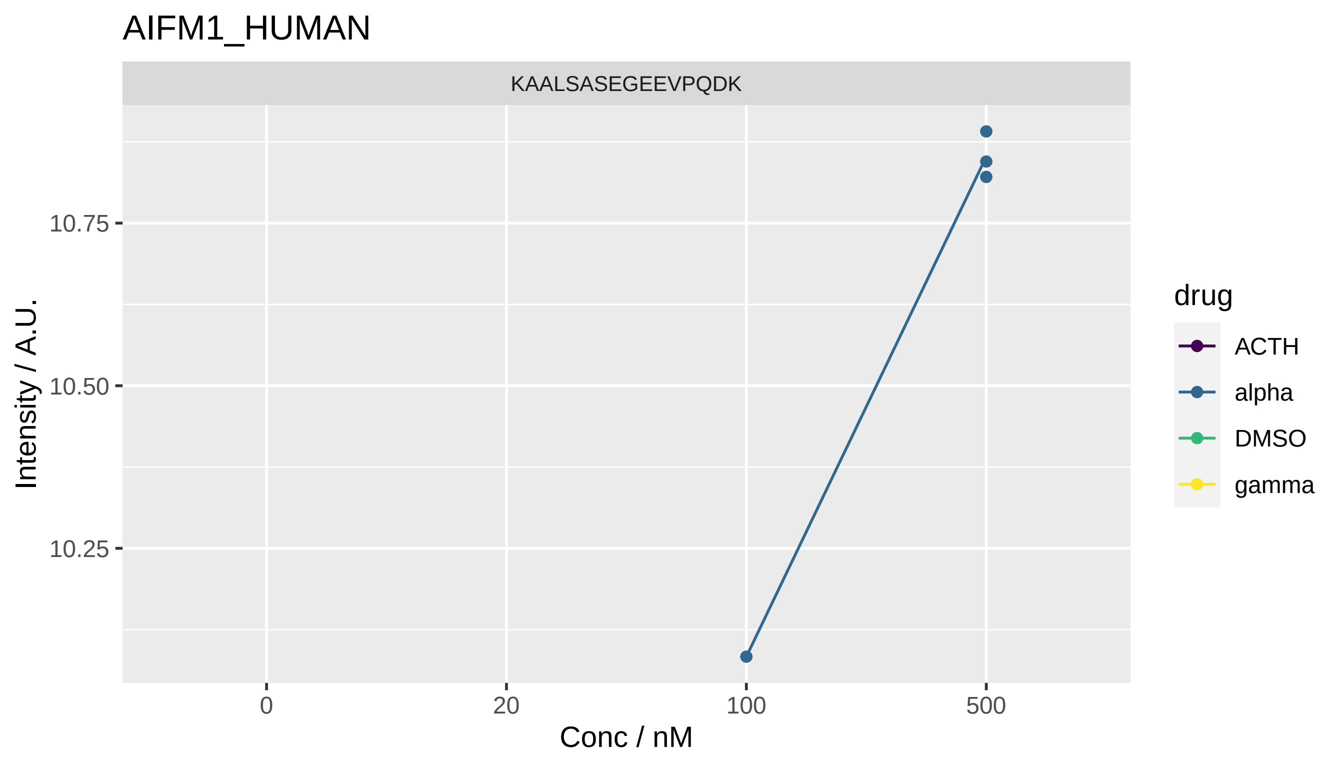
AIFM1_HUMAN
KAALSASEGEEVPQDK
Intensity / A.U.
10.75
10.50
10.25
0
20
100
500
Conc / nM
drug
ACTH
alpha
DMSO
gamma

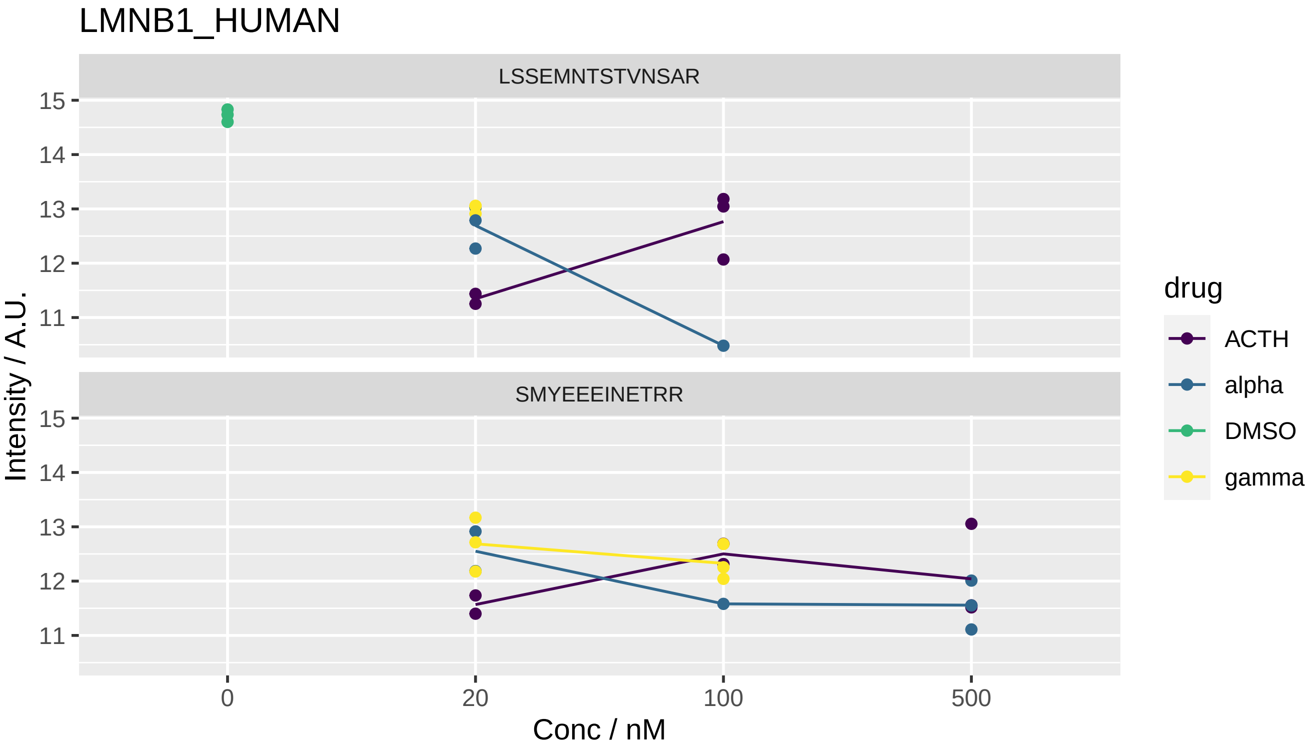
LMNB1_HUMAN
LSSEMNTSTVNSAR
SMYEEEINETRR
Intensity / A.U.
15
14
13
12
11
0
20
100
500
Conc / nM
drug
ACTH
alpha
DMSO
gamma

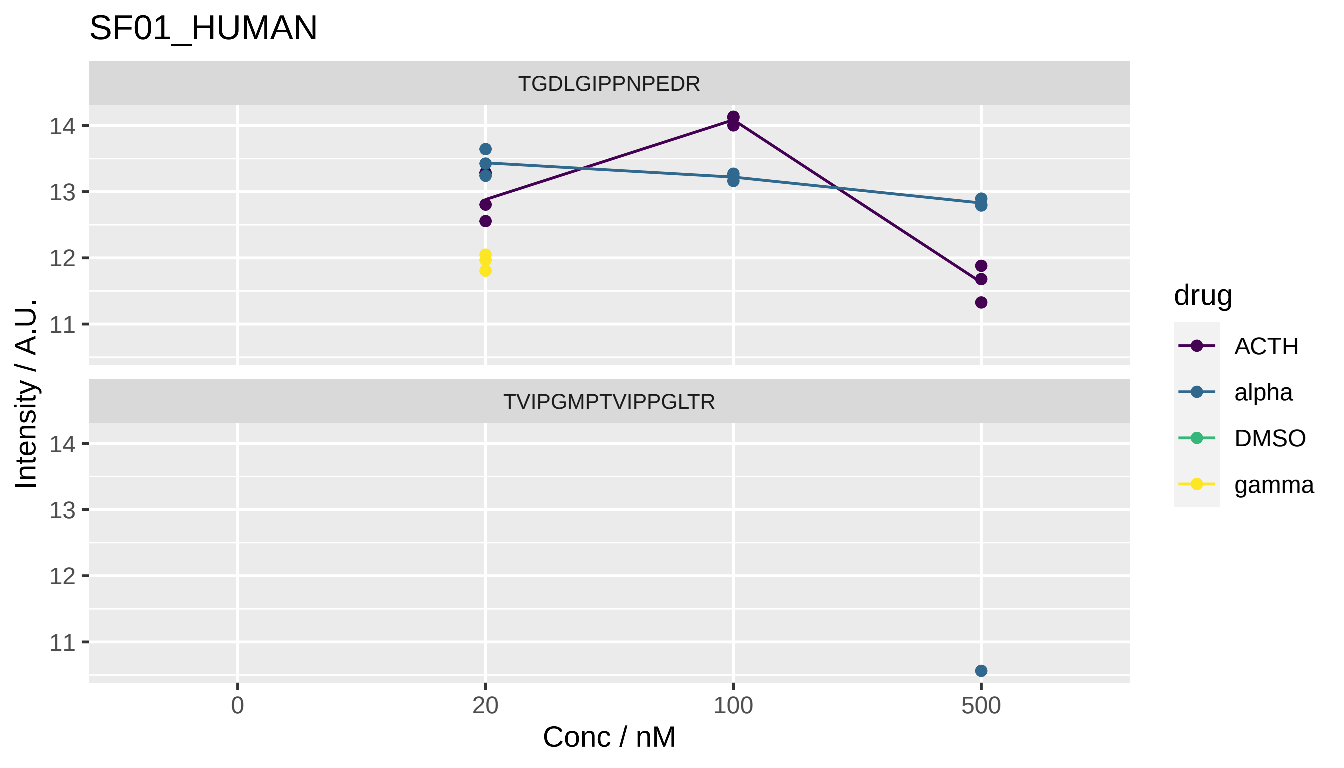

SF01_HUMAN
TGDLGIPPNPEDR
TVIPGMPTVIPPGLTR
Intensity / A.U.
Conc / nM
14
13
12
11
0
20
100
500
drug
ACTH
alpha
DMSO
gamma

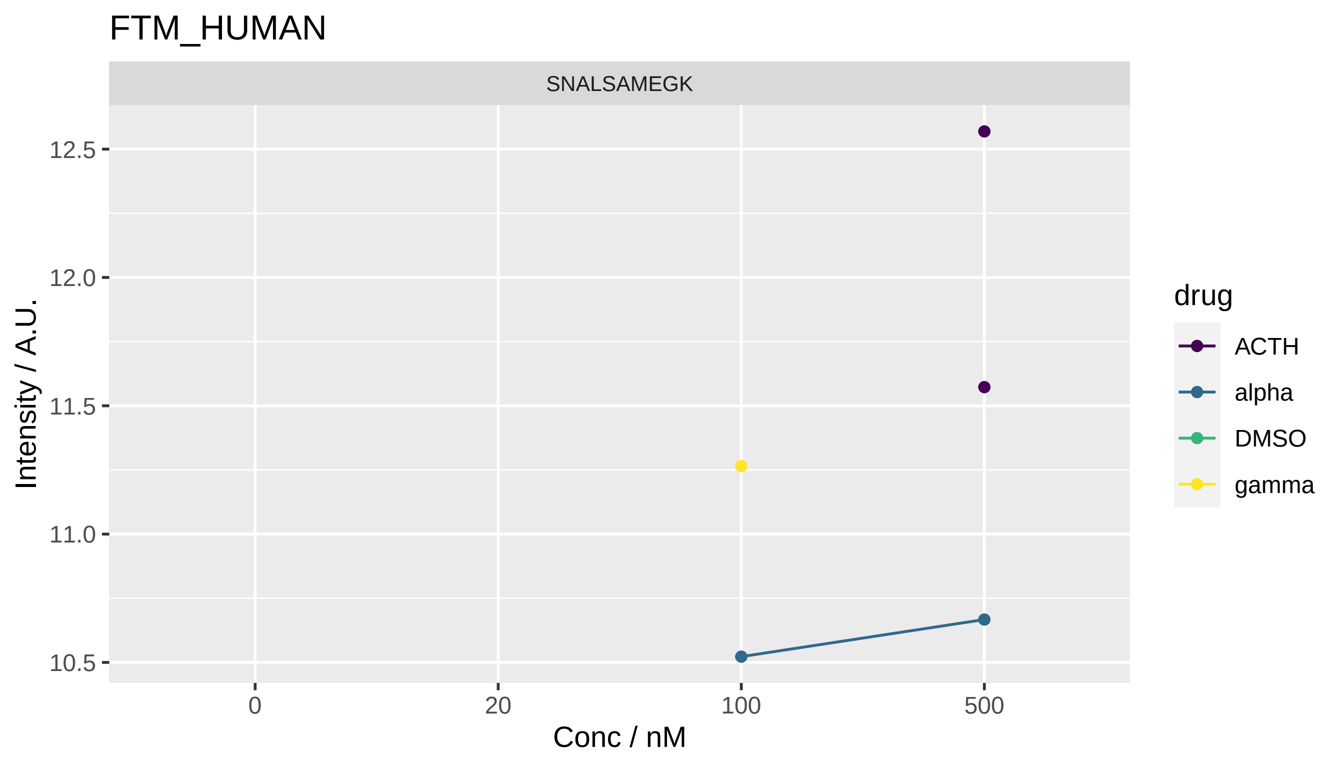

FTM_HUMAN
SNALSAMEGK
12.5
12.0
11.5
11.0
10.5
Intensity / A.U.
0
20
100
500
Conc / nM
drug
ACTH
alpha
DMSO
gamma

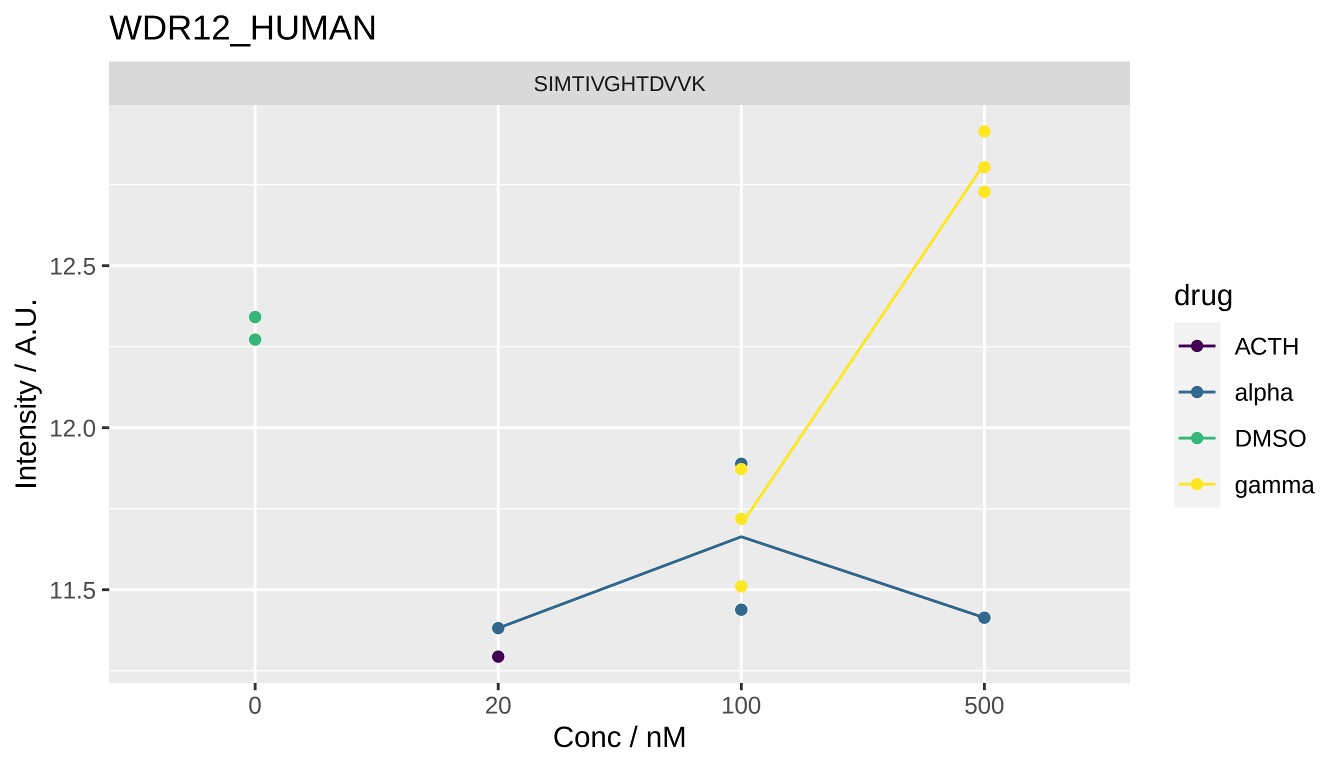

WDR12_HUMAN
SIMTIVGHTDVVK
Intensity / A.U.
12.5
12.0
11.5
0
20
100
500
Conc / nM
drug
ACTH
alpha
DMSO
gamma

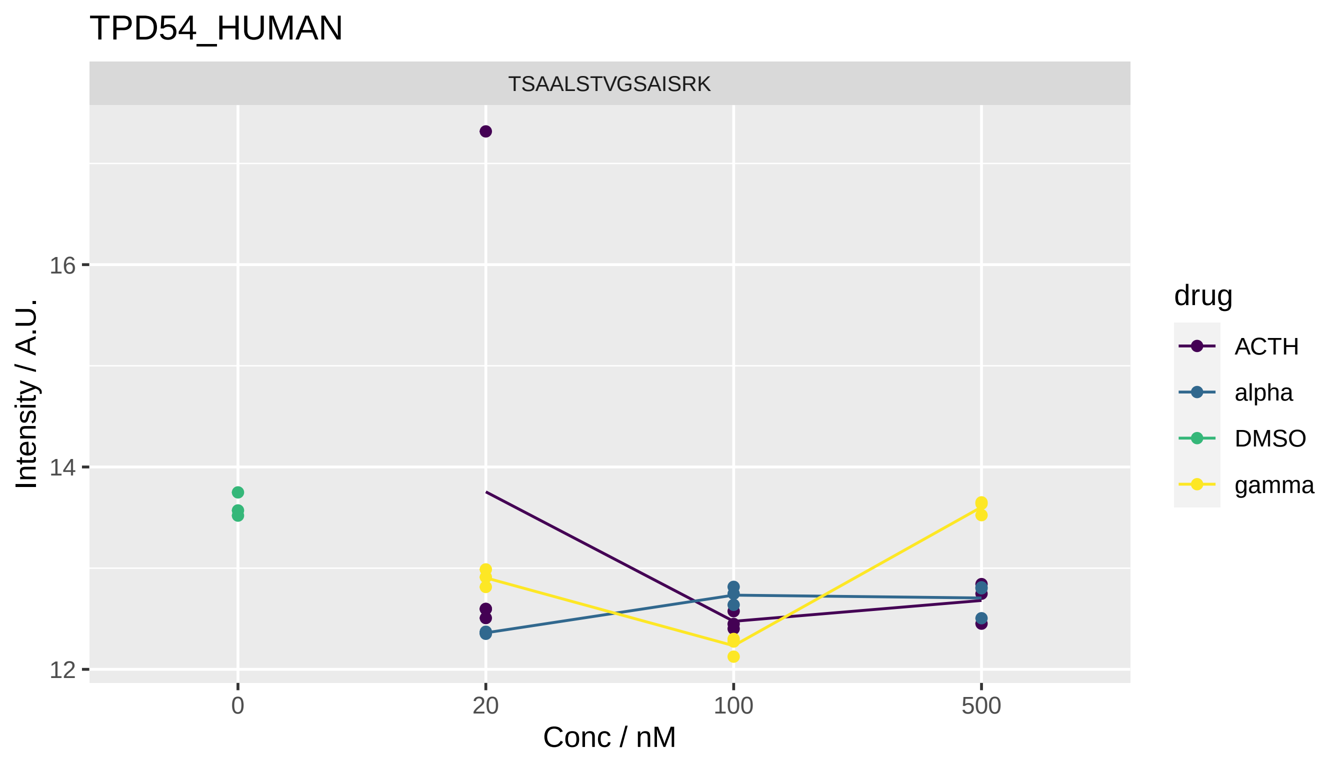
TPD54_HUMAN
TSAALSTVGSAISRK
Intensity / A.U.
16
14
12
0
20
100
500
Conc / nM
drug
ACTH
alpha
DMSO
gamma

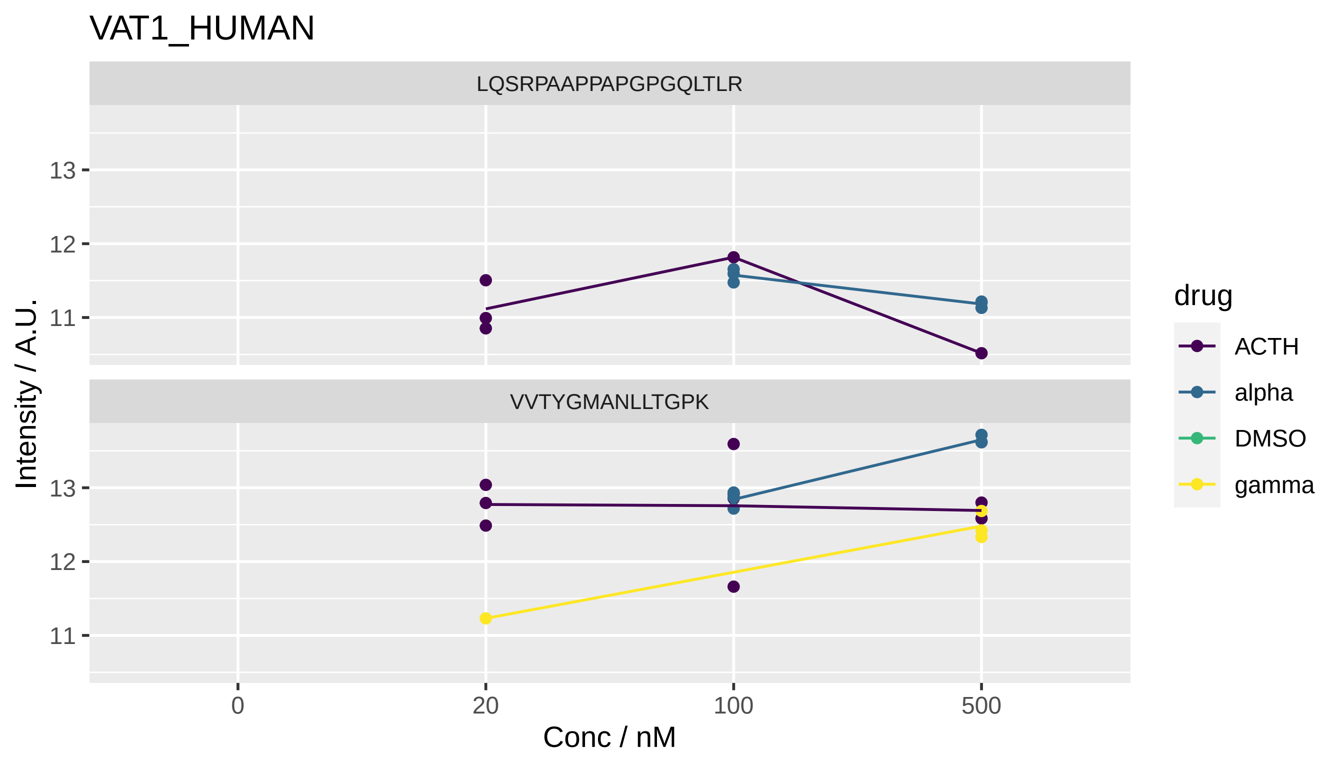
VAT1_HUMAN
LQSRPAAPPAPGPGQLTLR
VVTYGMANLLTGPK
Intensity / A.U.
13
12
11
0
20
100
500
Conc / nM
drug
ACTH
alpha
DMSO
gamma

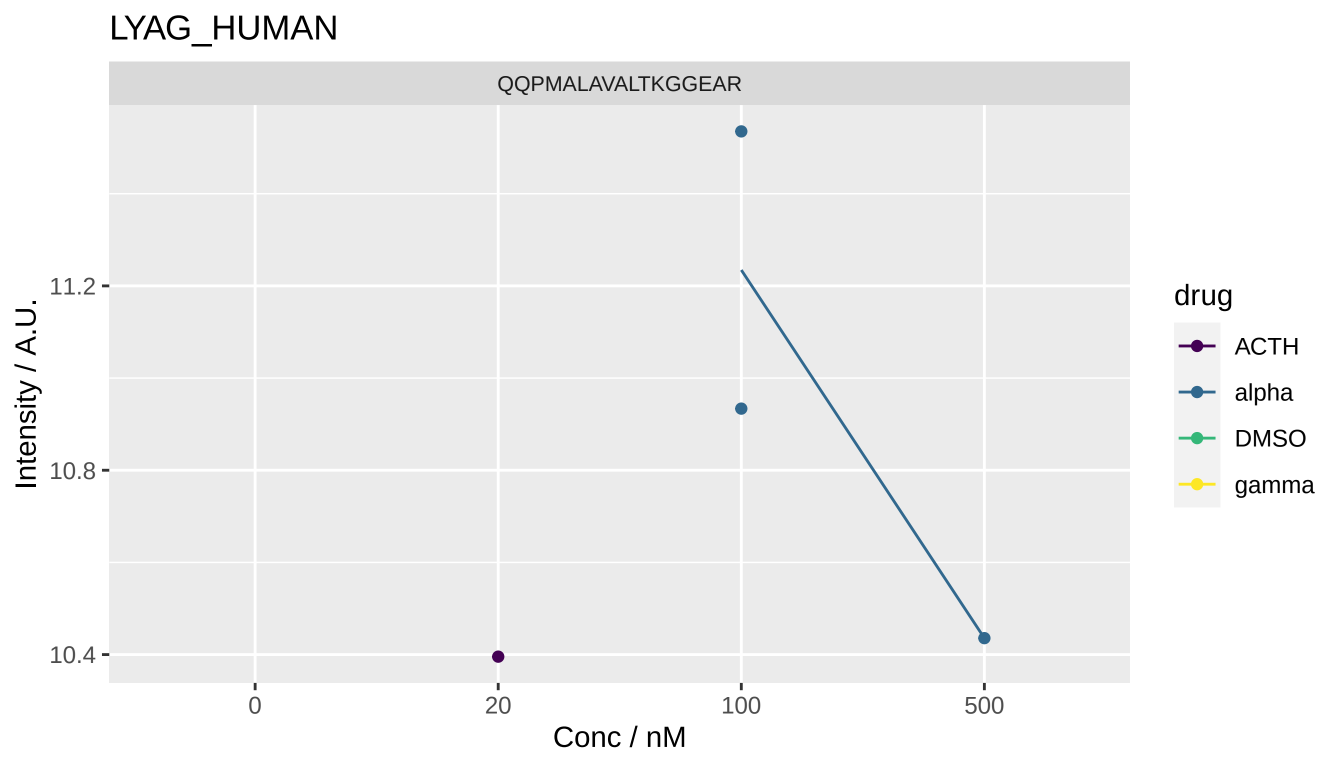
LYAG_HUMAN
QQPMALAVALTKGGEAR
Intensity / A.U.
11.2
10.8
10.4
0
20
100
500
Conc / nM
drug
ACTH
alpha
DMSO
gamma

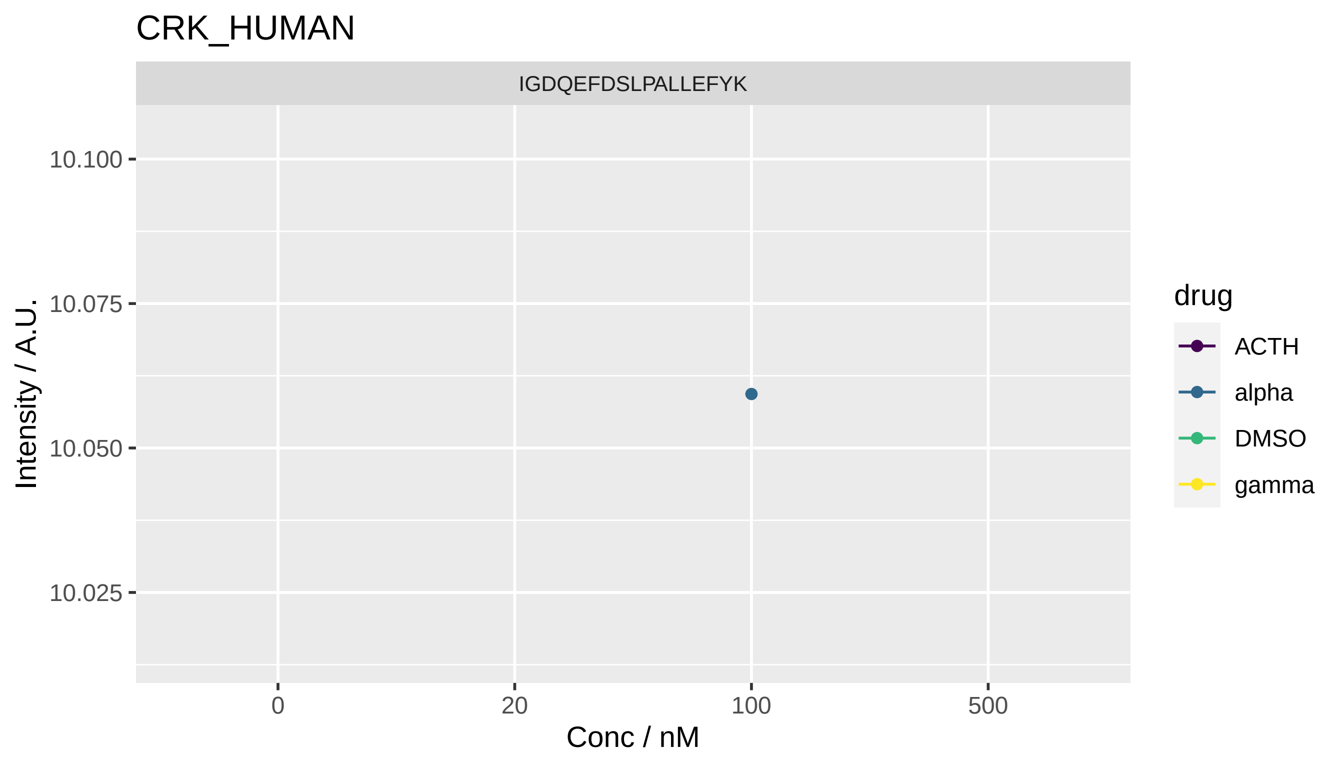

CRK_HUMAN
IGDQEFDSLPALLEFYK
10.100
10.075
10.050
10.025
Intensity / A.U.
0
20
100
500
Conc / nM
drug
ACTH
alpha
DMSO
gamma

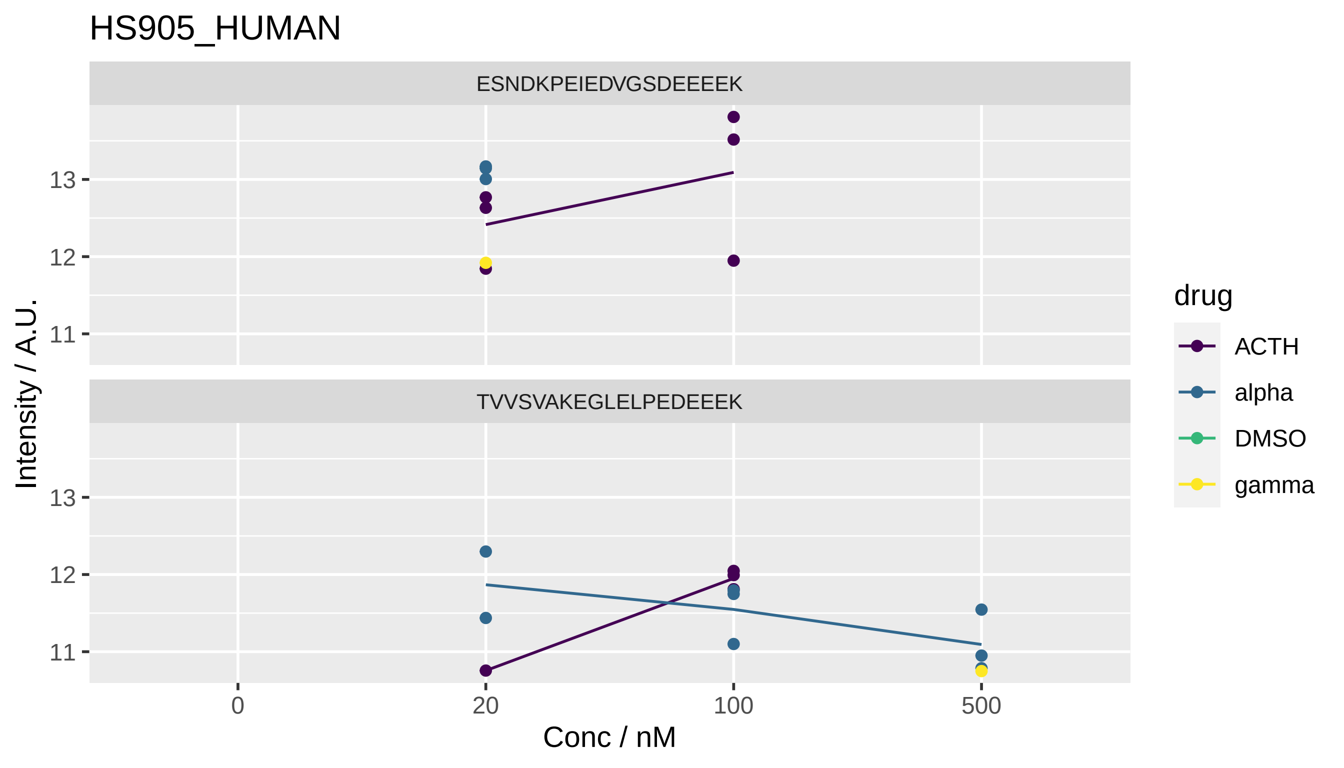
HS905_HUMAN
ESNDKPEIEDVGSDEEEEK
TVVSVAKEGLELPEDEEEK
Intensity / A.U.
13
12
11
13
12
11
0
20
100
500
Conc / nM
drug
ACTH
alpha
DMSO
gamma

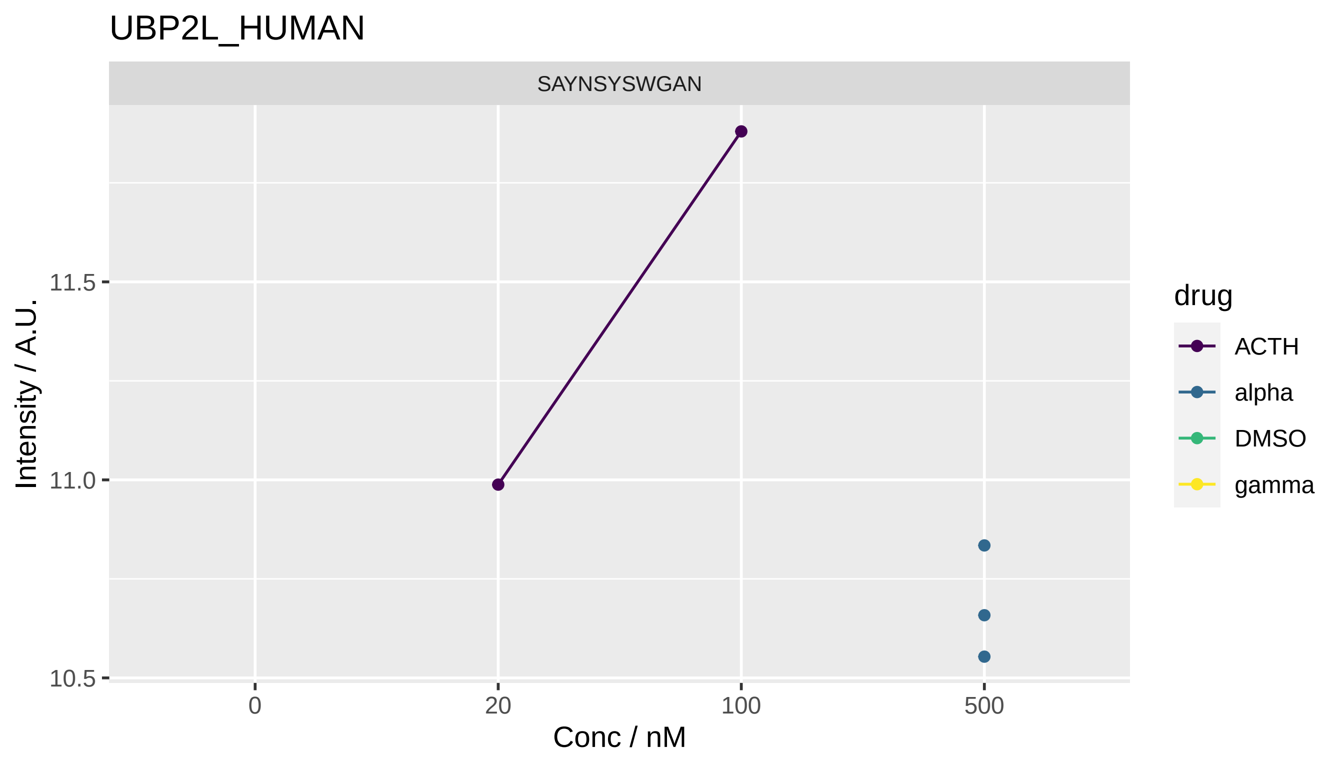

UBP2L_HUMAN
SAYNSYSWGAN
Intensity / A.U.
11.5
11.0
10.5
0
20
100
500
Conc / nM
drug
ACTH
alpha
DMSO
gamma

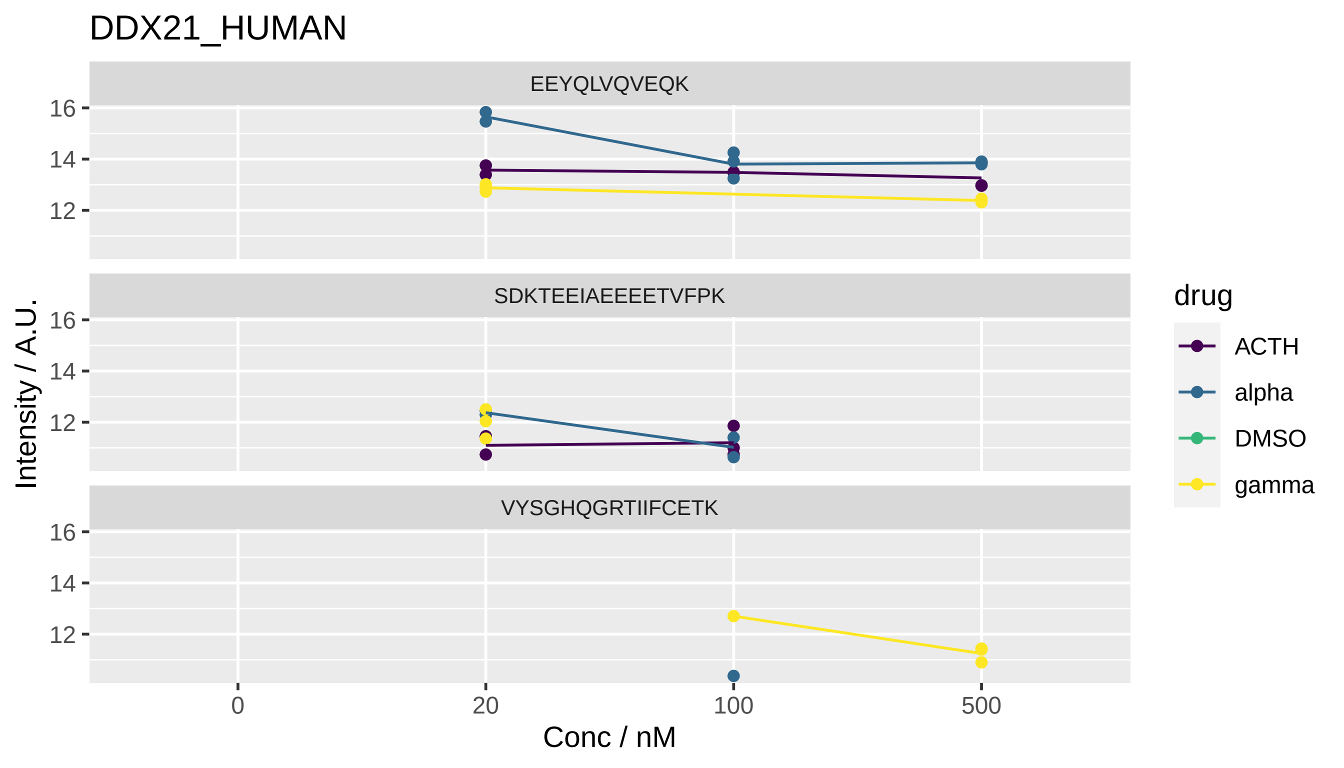

DDX21_HUMAN
EEYQLVQVEQK
SDKTEEIAEEEETVFPK
VYSGHQGRTIIFCETK
16
14
12
Intensity / A.U.
0
20
100
500
Conc / nM
drug
ACTH
alpha
DMSO
gamma

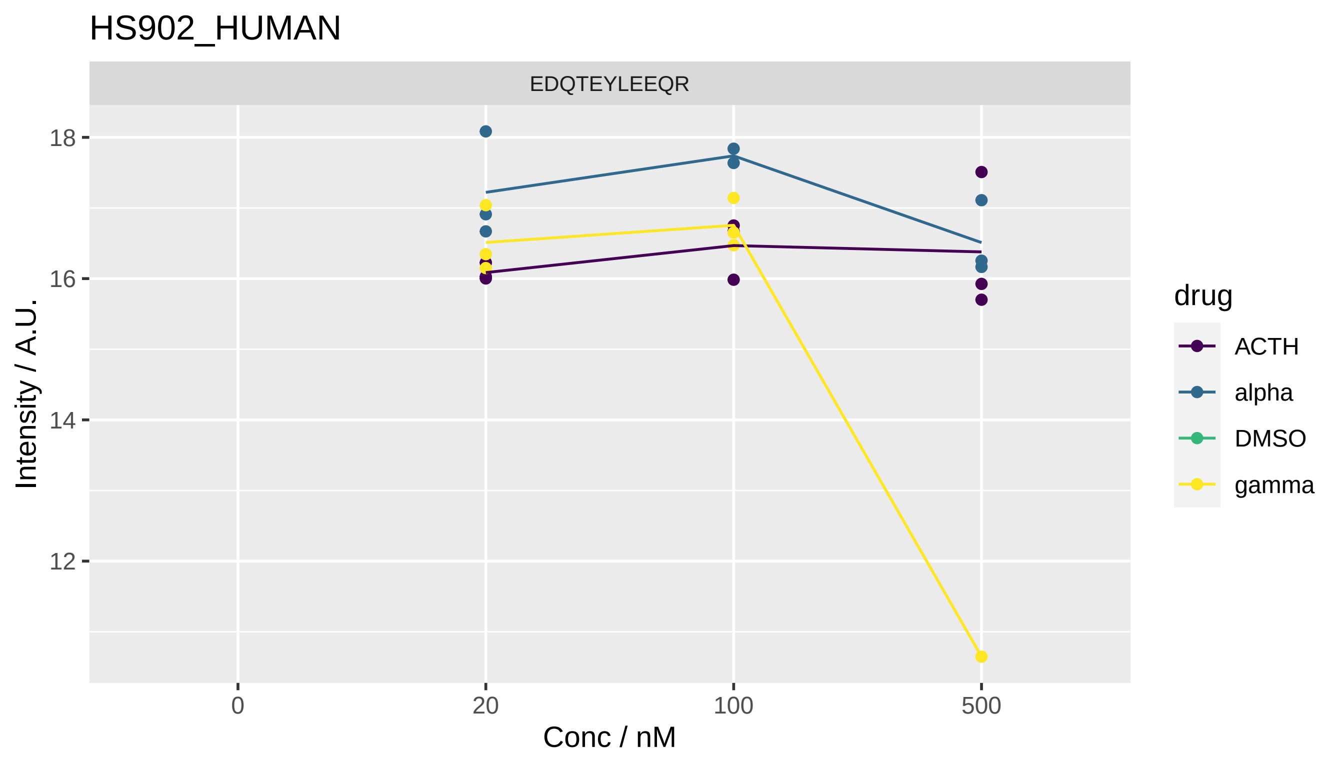
HS902_HUMAN
EDQTEYLEEQR
18
16
14
12
Intensity / A.U.
0
20
100
500
Conc / nM
drug
ACTH
alpha
DMSO
gamma

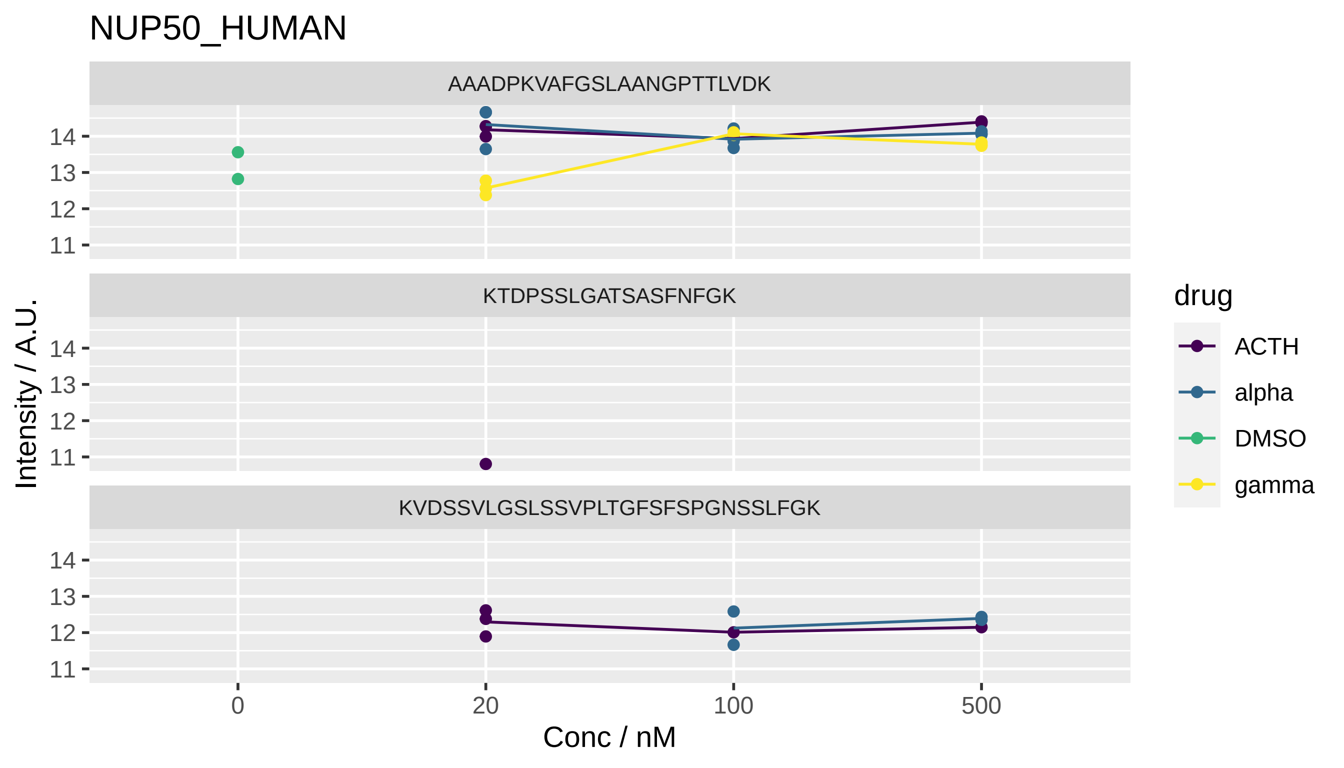

NUP50_HUMAN
AAADPKVAFGSLAANGPTTLVDK
KTDPSSLGATSASFNFGK
KVDSSVLGSLSSVPLTGFSFSPGNSSLFGK
Intensity / A.U.
14
13
12
11
0
20
100
500
Conc / nM
drug
ACTH
alpha
DMSO
gamma

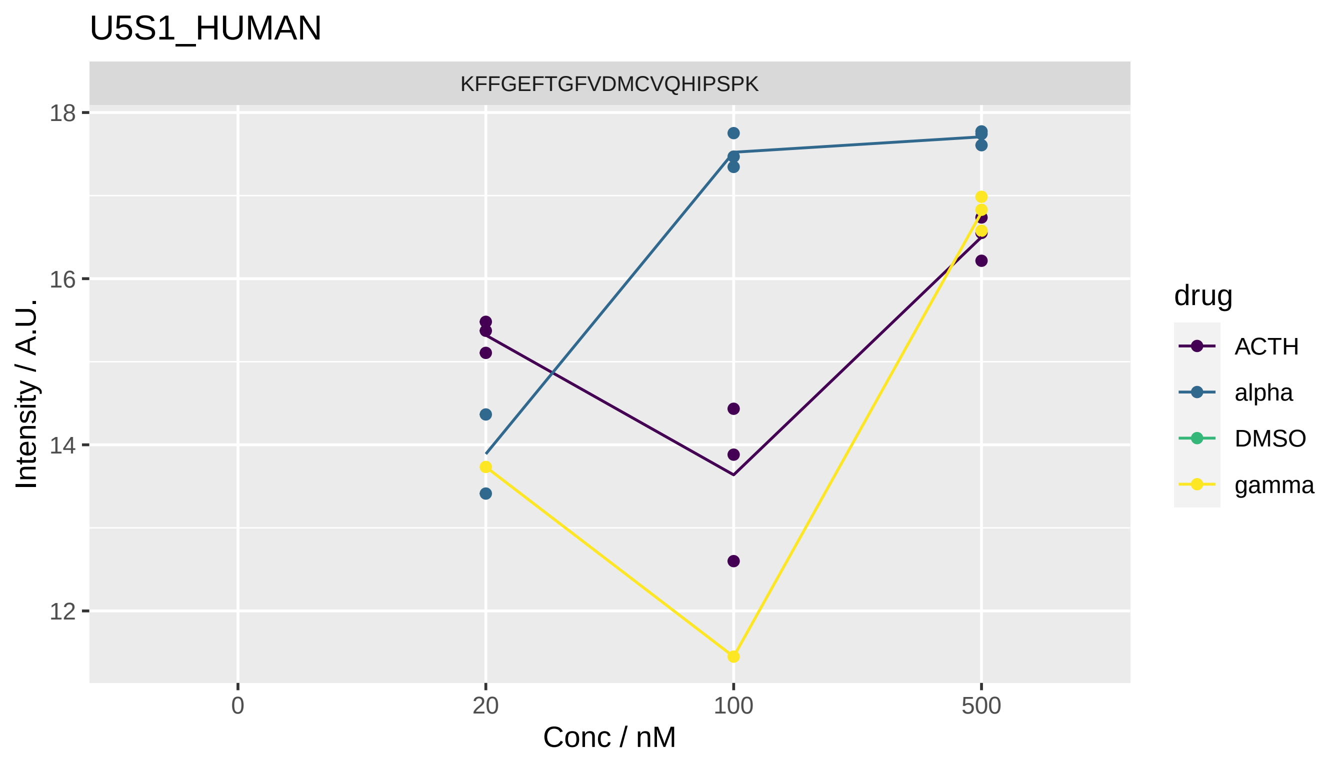
U5S1_HUMAN
KFFGEFTGFVDMCVQHIPSPK
Intensity / A.U.
18
16
14
12
0
20
100
500
Conc / nM
drug
ACTH
alpha
DMSO
gamma

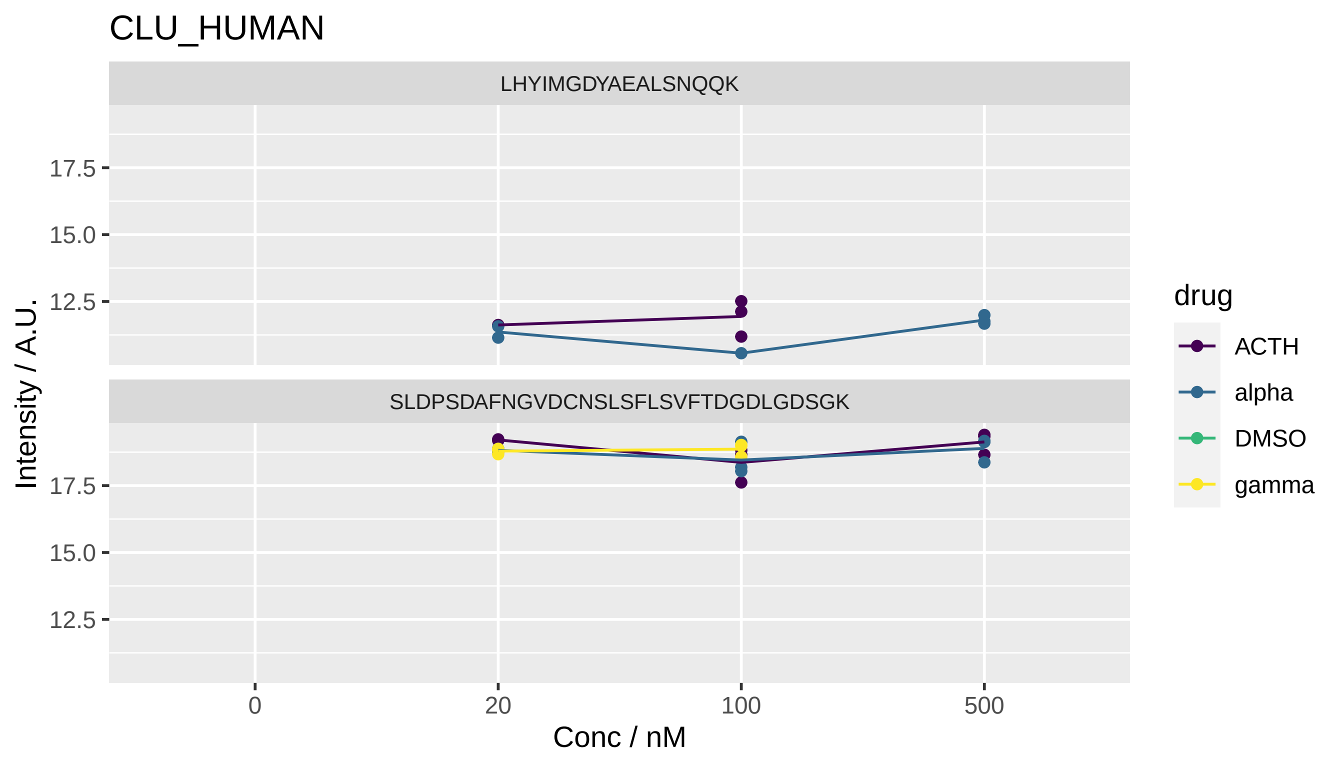
CLU_HUMAN
LHYIMGDYAEALSNQQK
SLDPSDAFNGVDCNSLSFLSVFTDGDLGDSGK
Intensity / A.U.
17.5
15.0
12.5
17.5
15.0
12.5
0
20
100
500
Conc / nM
drug
ACTH
alpha
DMSO
gamma

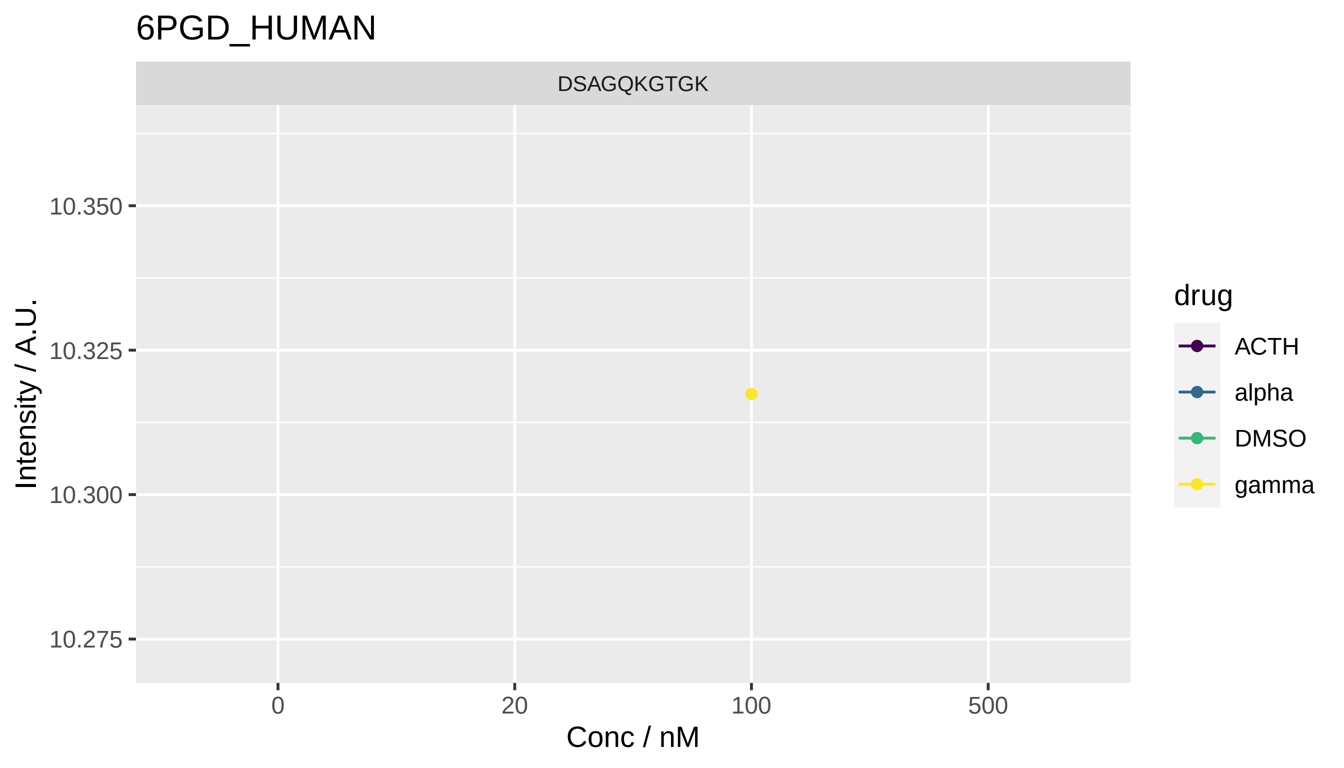
6PGD_HUMAN
DSAGQKGTGK
Intensity / A.U.
10.350
10.325
10.300
10.275
0
20
100
500
Conc / nM
drug
ACTH
alpha
DMSO
gamma

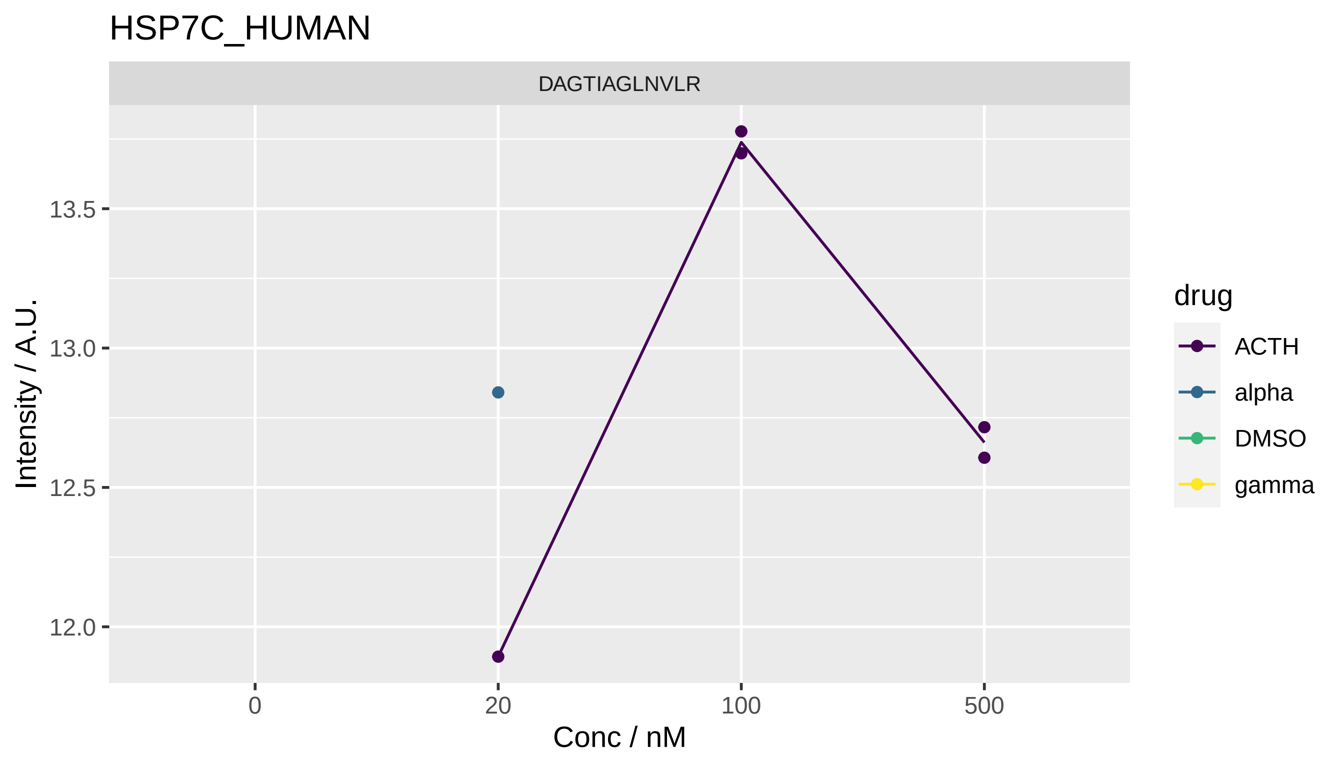
HSP7C_HUMAN
DAGTIAGLNVLR
Intensity / A.U.
13.5
13.0
12.5
12.0
0
20
100
500
Conc / nM
drug
ACTH
alpha
DMSO
gamma

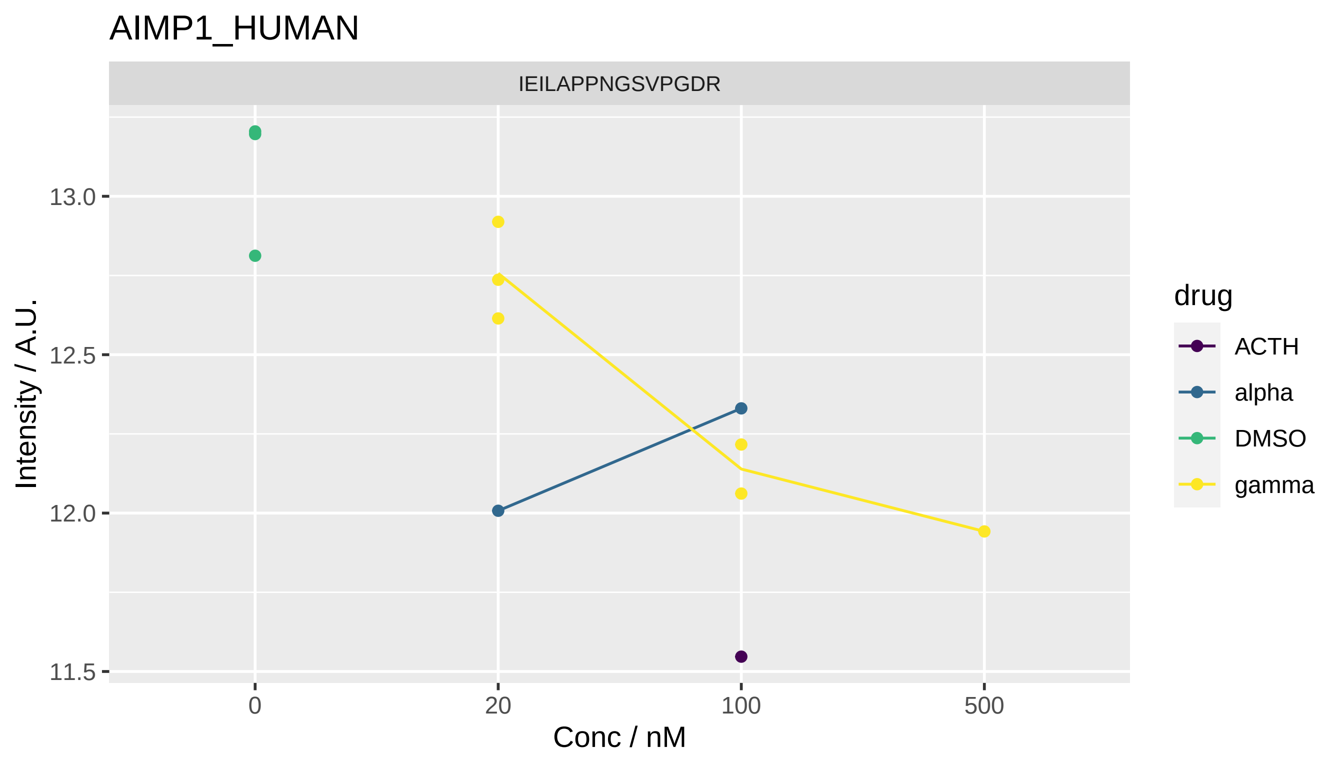
AIMP1_HUMAN
IEILAPPNGSVPGDR
13.0
12.5
12.0
11.5
Intensity / A.U.
0
20
100
500
Conc / nM
drug
ACTH
alpha
DMSO
gamma

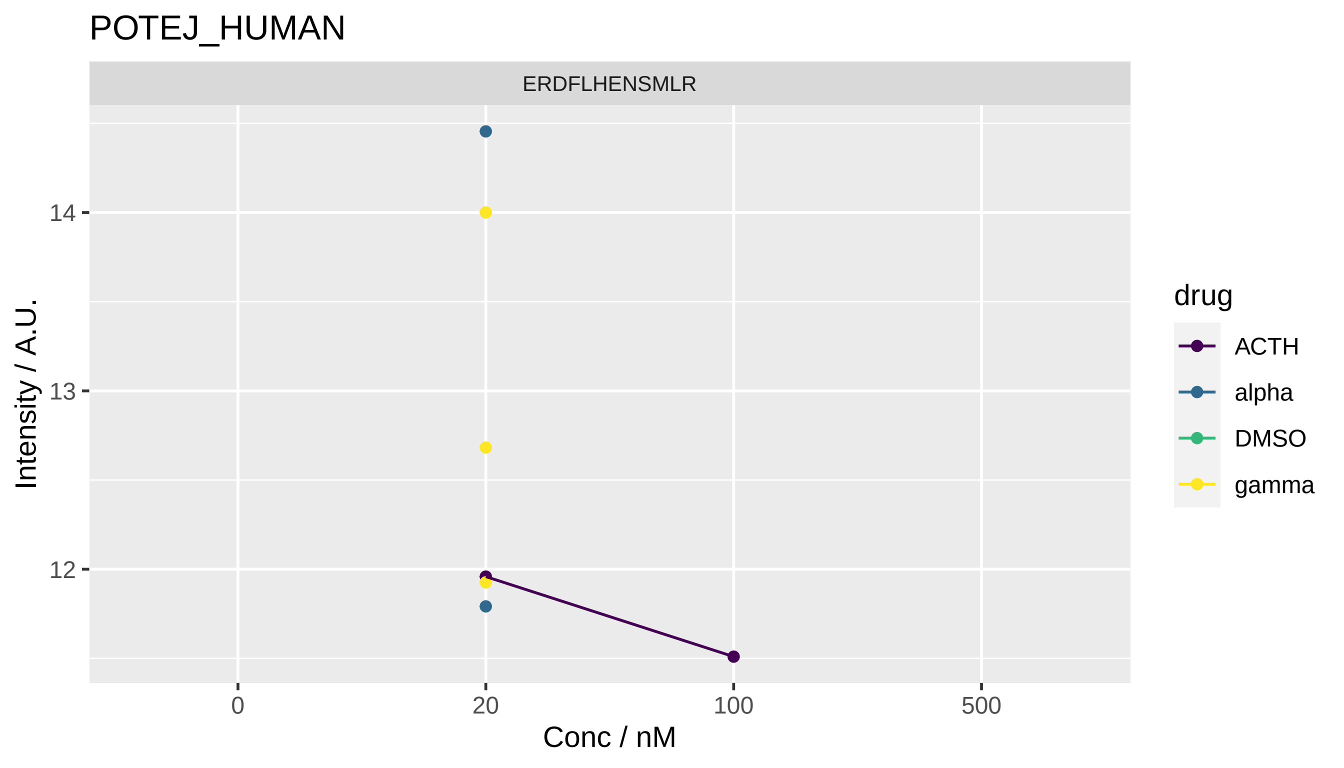
POTEJ_HUMAN
ERDFLHENSMLR
Intensity / A.U.
14
13
12
0
20
100
500
Conc / nM
drug
ACTH
alpha
DMSO
gamma

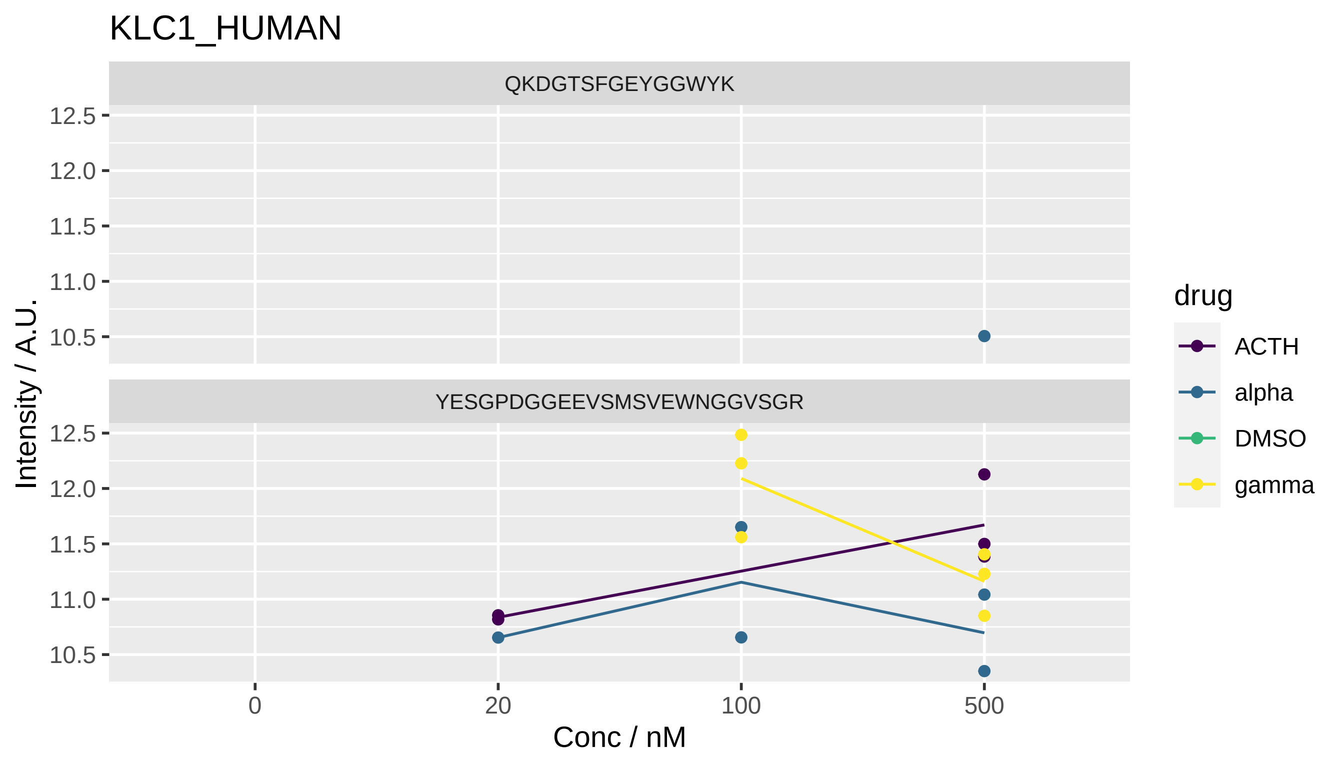
KLC1_HUMAN
QKDGTSFGEYGGWYK
YESGPDGGEEVSMSVEWNGGVSGR
Intensity / A.U.
12.5
12.0
11.5
11.0
10.5
0
20
100
500
Conc / nM
drug
ACTH
alpha
DMSO
gamma

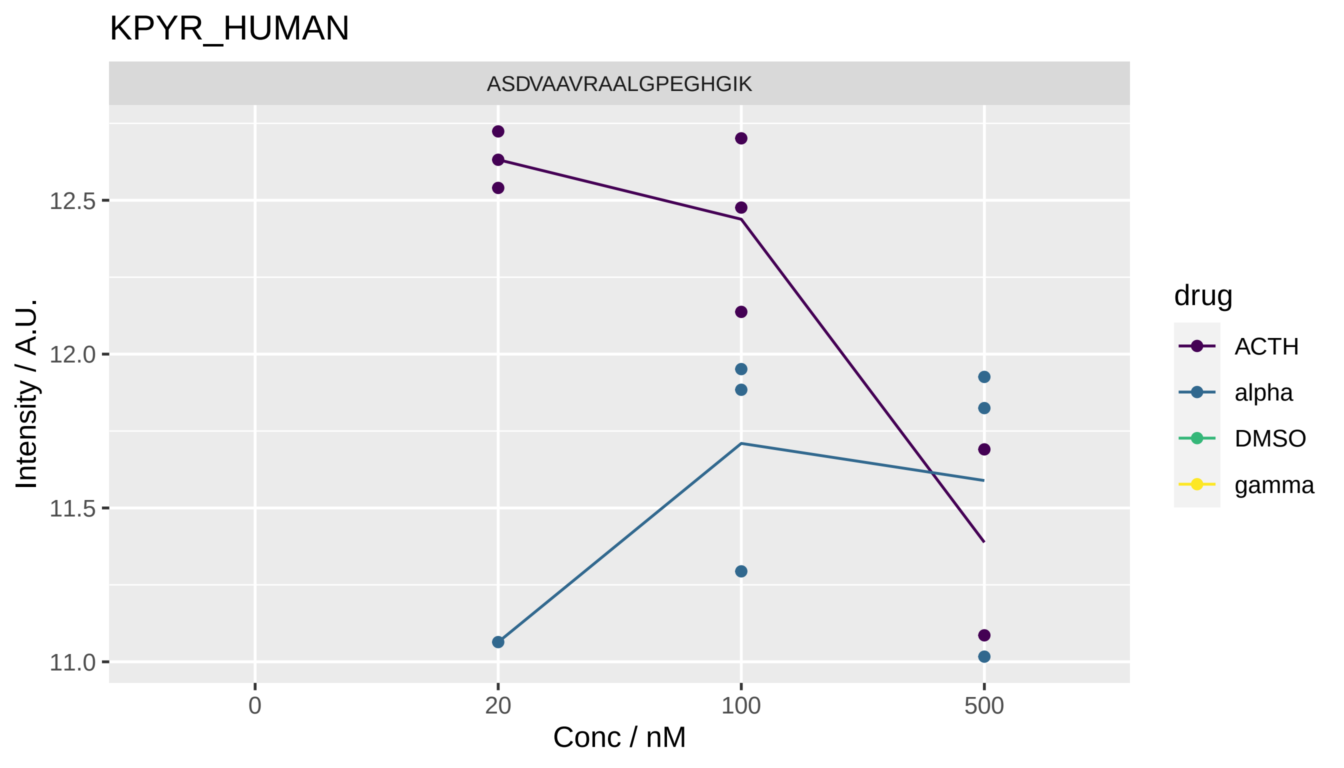

KPYR_HUMAN
ASDVAAVRAALGPEGHGIK
Intensity / A.U.
12.5
12.0
11.5
11.0
0
20
100
500
Conc / nM
drug
ACTH
alpha
DMSO
gamma

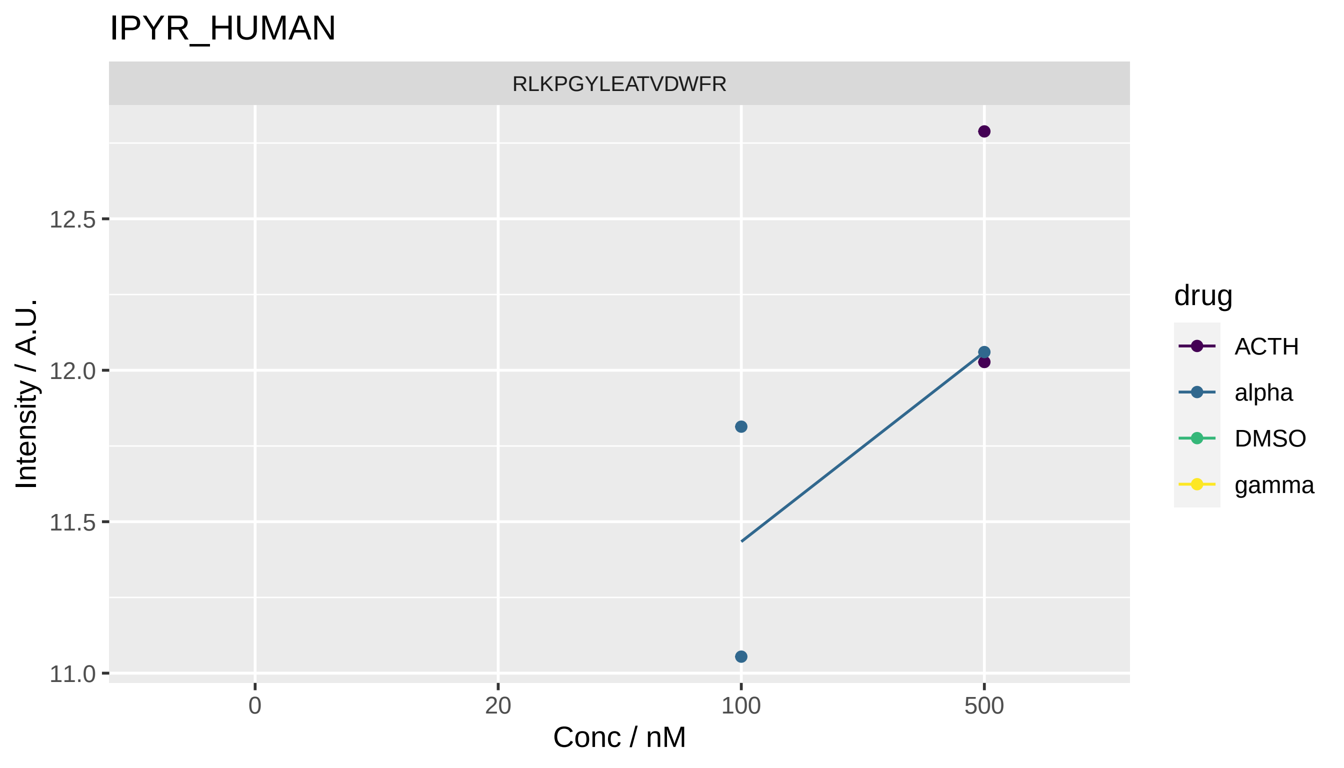
IPYR_HUMAN
RLKPGYLEATVDWFR
Intensity / A.U.
12.5
12.0
11.5
11.0
0
20
100
500
Conc / nM
drug
ACTH
alpha
DMSO
gamma

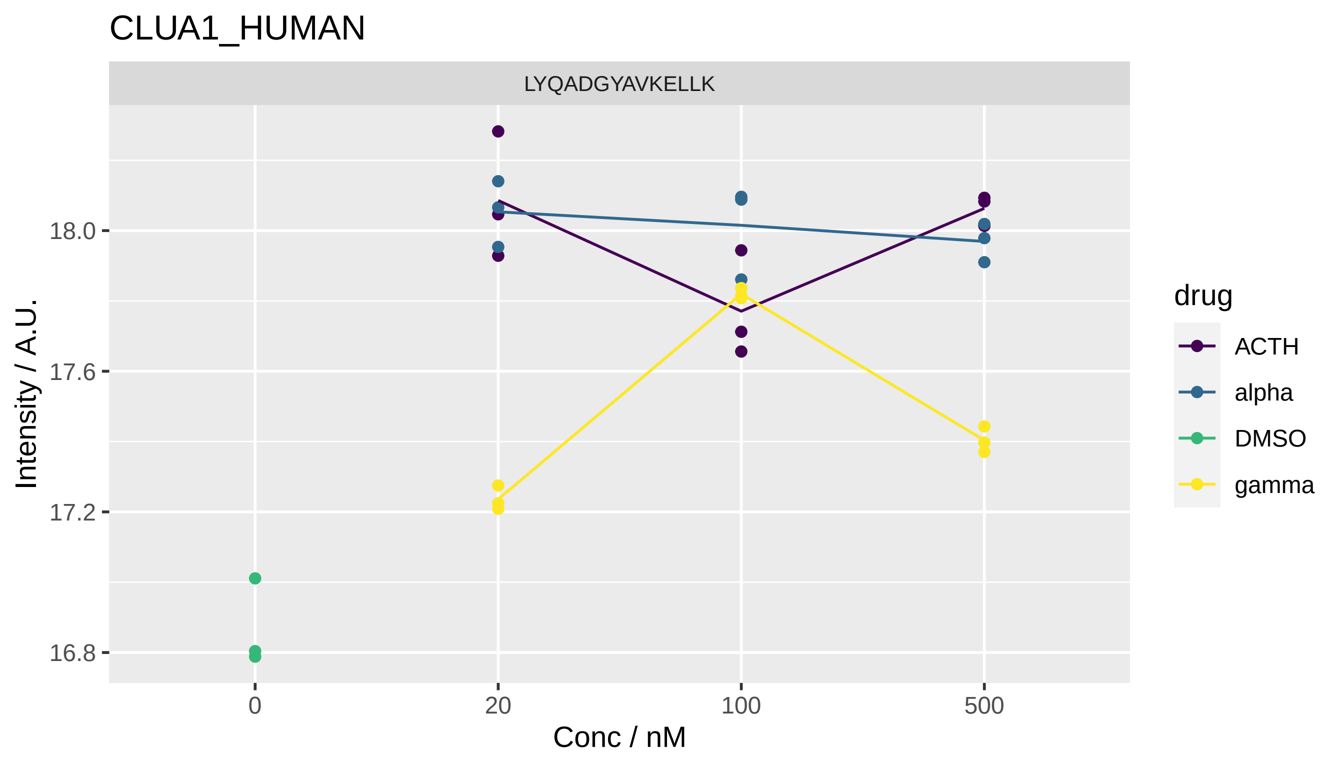
CLUA1_HUMAN
LYQADGYAVKELLK
Intensity / A.U.
18.0
17.6
17.2
16.8
0
20
100
500
Conc / nM
drug
ACTH
alpha
DMSO
gamma

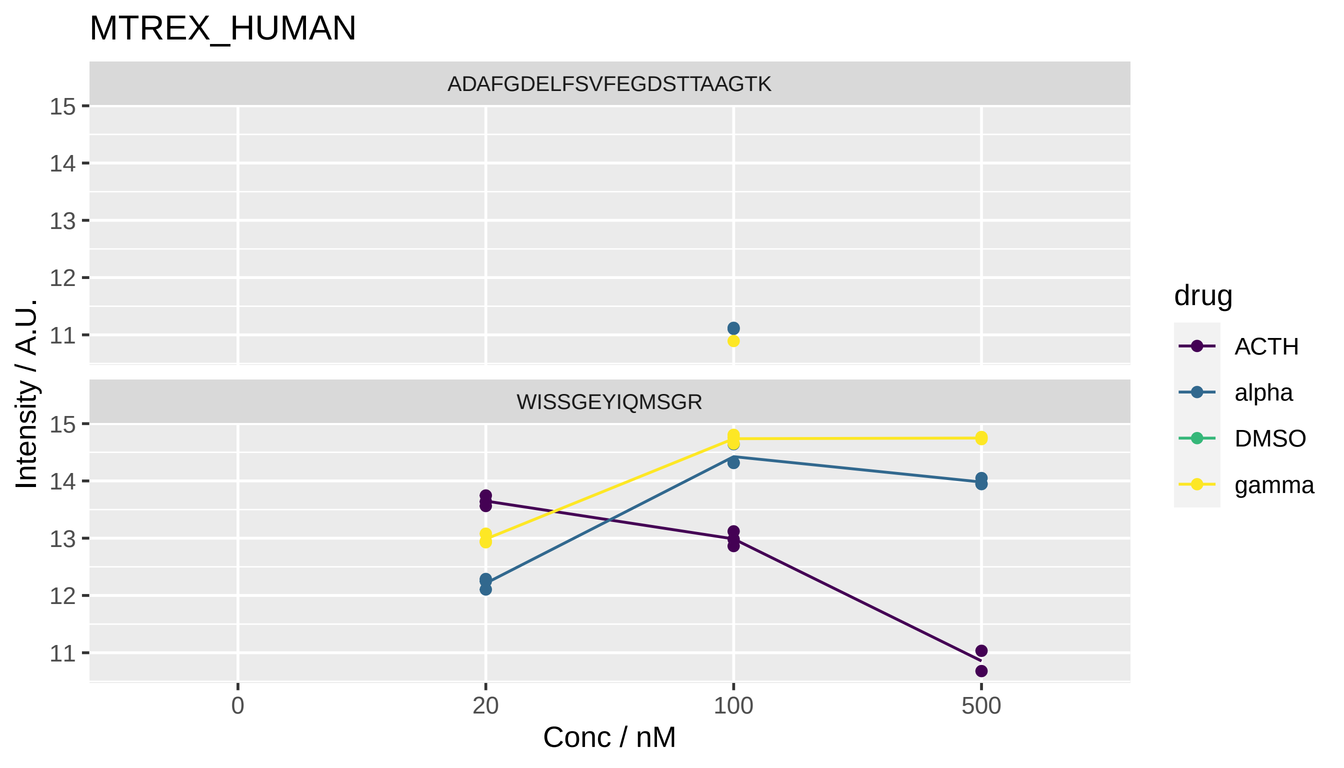

MTREX_HUMAN
ADAFGDELFSVFEGDSTTAAGTK
WISSGEYIQMSGR
Intensity / A.U.
Conc / nM
0
20
100
500
11
12
13
14
15
drug
ACTH
alpha
DMSO
gamma

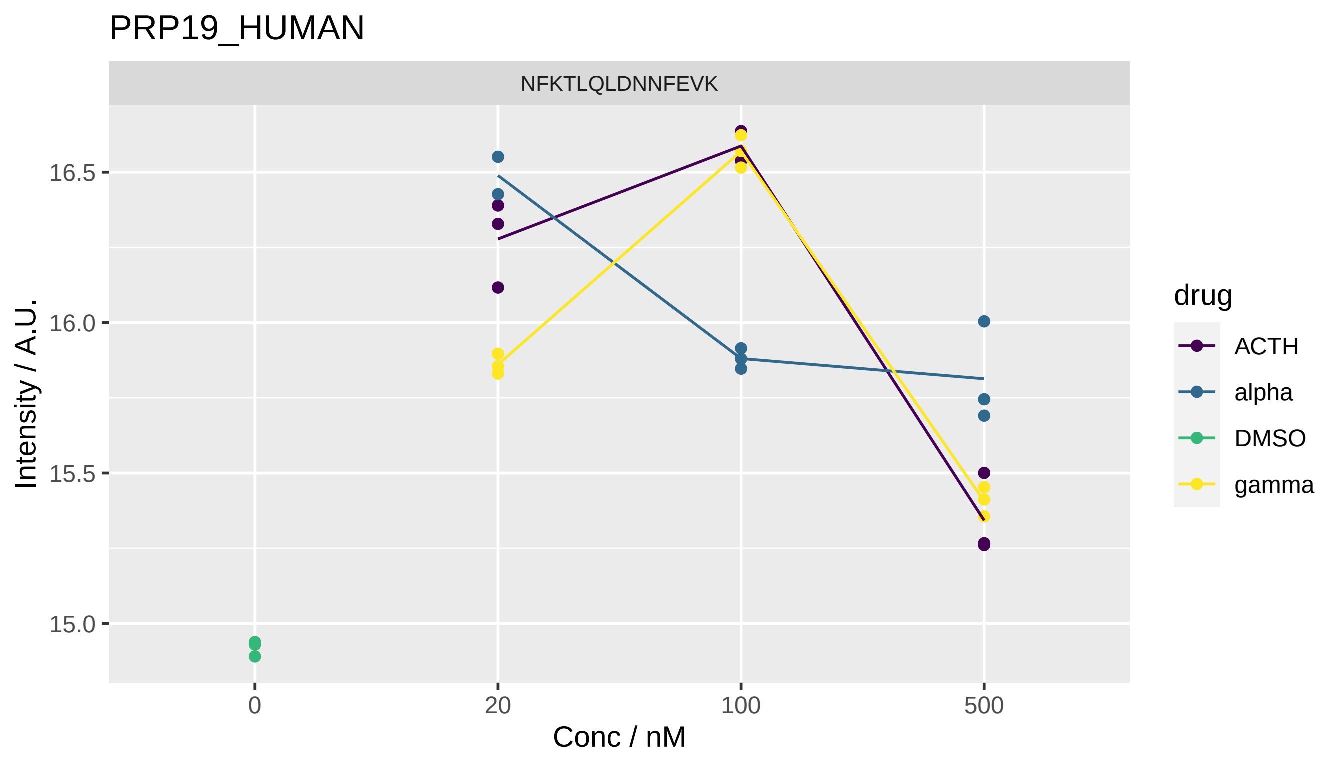
PRP19_HUMAN
NFKTLQLDNNFEVK
16.5
16.0
15.5
15.0
Intensity / A.U.
0
20
100
500
Conc / nM
drug
ACTH
alpha
DMSO
gamma

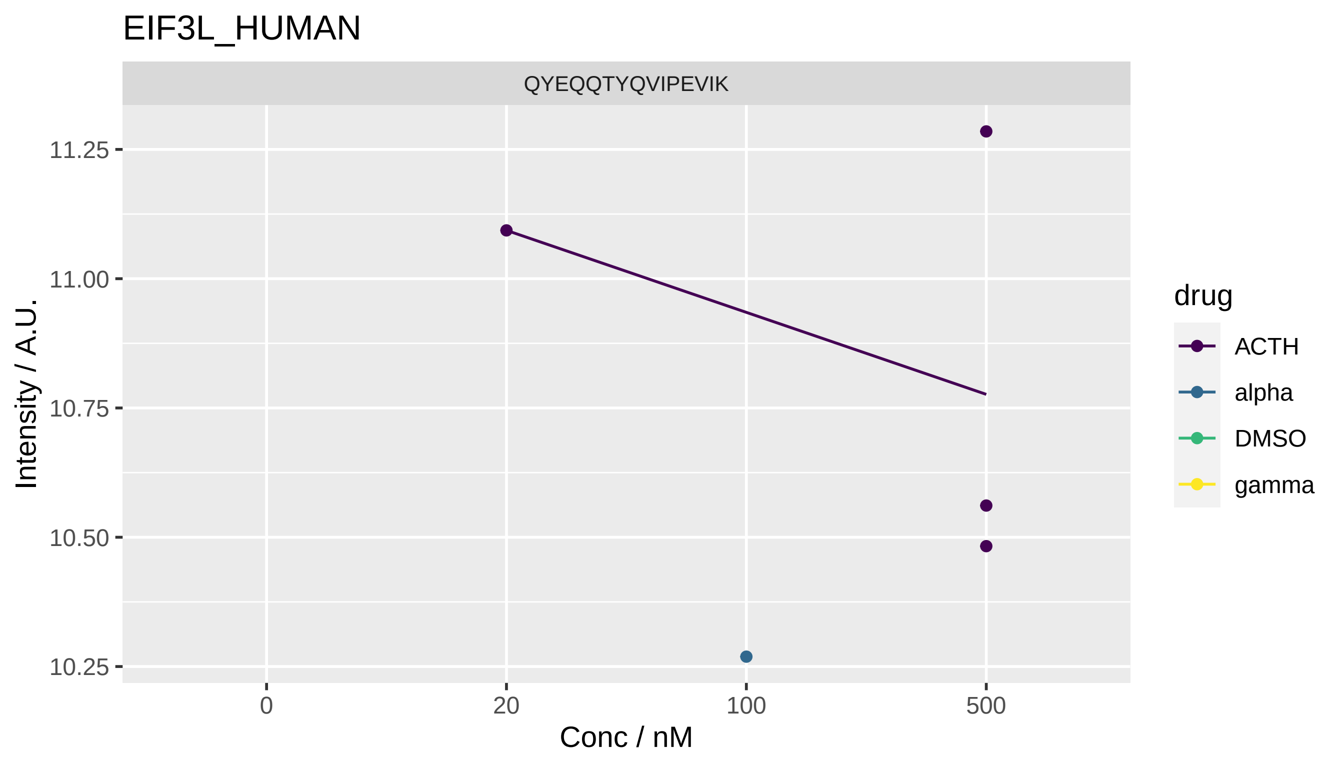
EIF3L_HUMAN
QYEQQTYQVIPEVIK
Intensity / A.U.
11.25
11.00
10.75
10.50
10.25
0
20
100
500
Conc / nM
drug
ACTH
alpha
DMSO
gamma

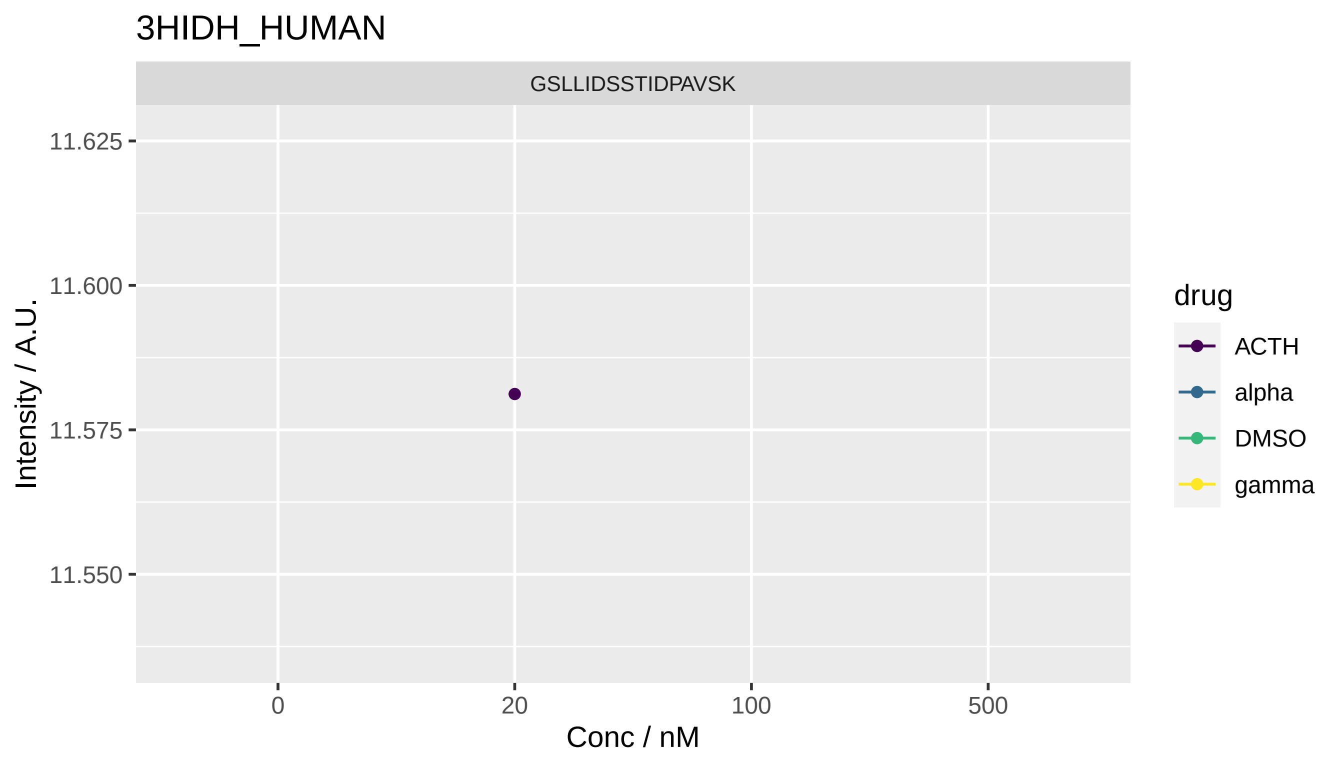
3HIDH_HUMAN
GSLLIDSSTIDPAVSK
11.625
11.600
11.575
11.550
Intensity / A.U.
0
20
100
500
Conc / nM
drug
ACTH
alpha
DMSO
gamma

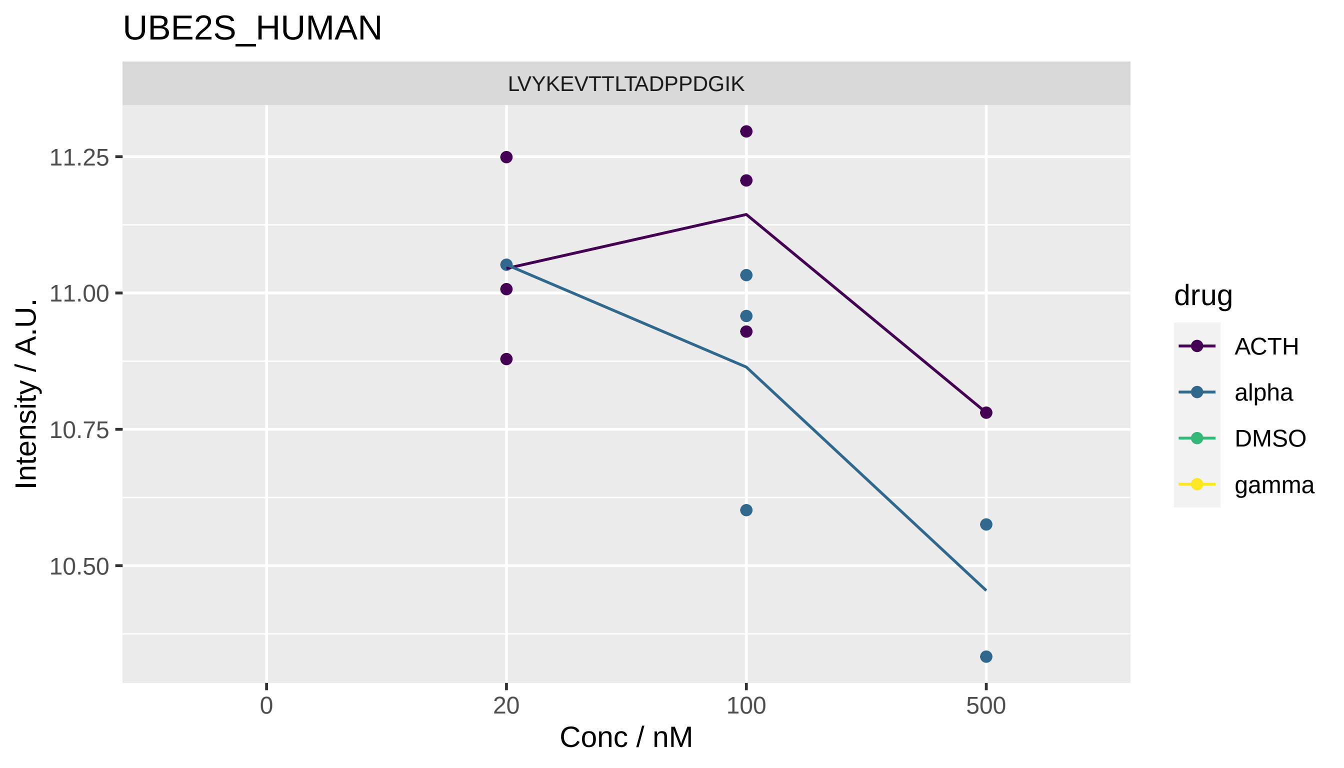
UBE2S_HUMAN
LVYKEVTTLTADPPDGIK
Intensity / A.U.
11.25
11.00
10.75
10.50
0
20
100
500
Conc / nM
drug
ACTH
alpha
DMSO
gamma

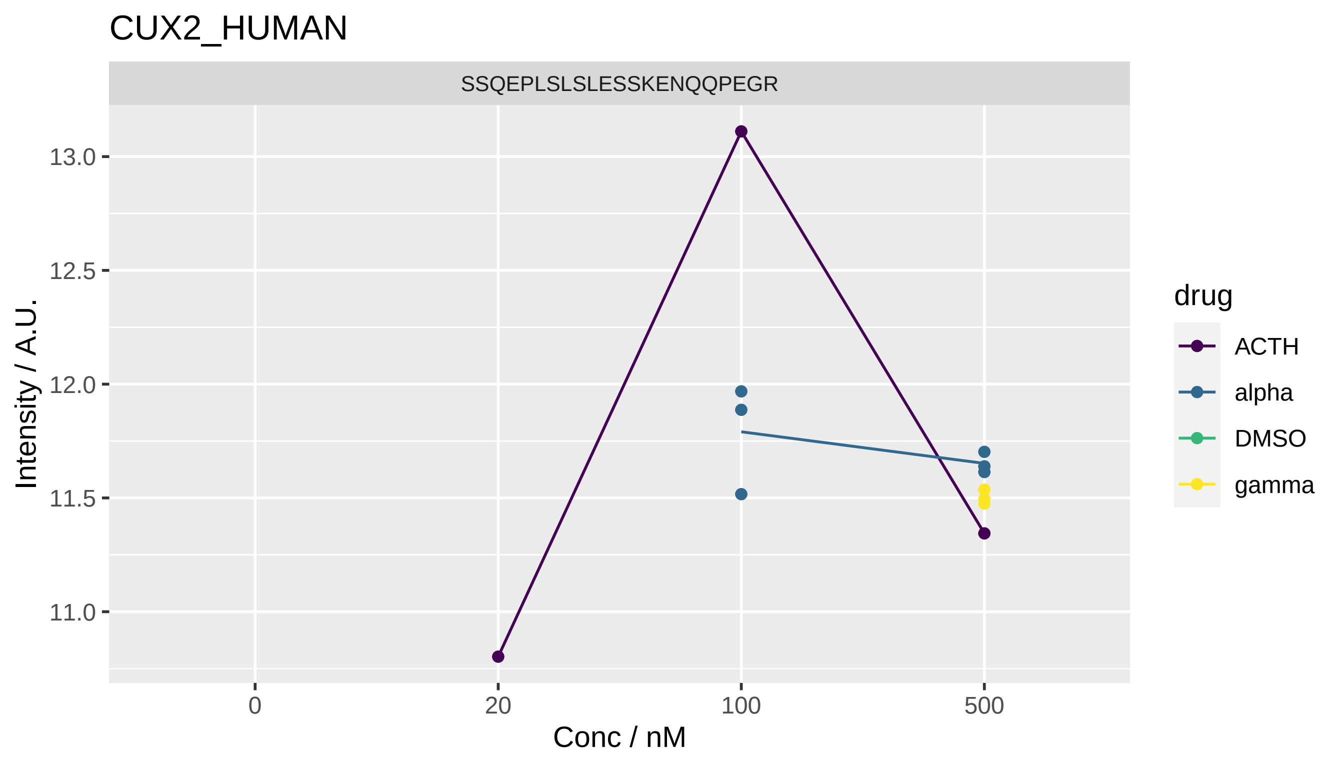

CUX2_HUMAN
SSQEPLSLSLESSKENQQPEGR
13.0
12.5
12.0
11.5
11.0
Intensity / A.U.
0
20
100
500
Conc / nM
drug
ACTH
alpha
DMSO
gamma

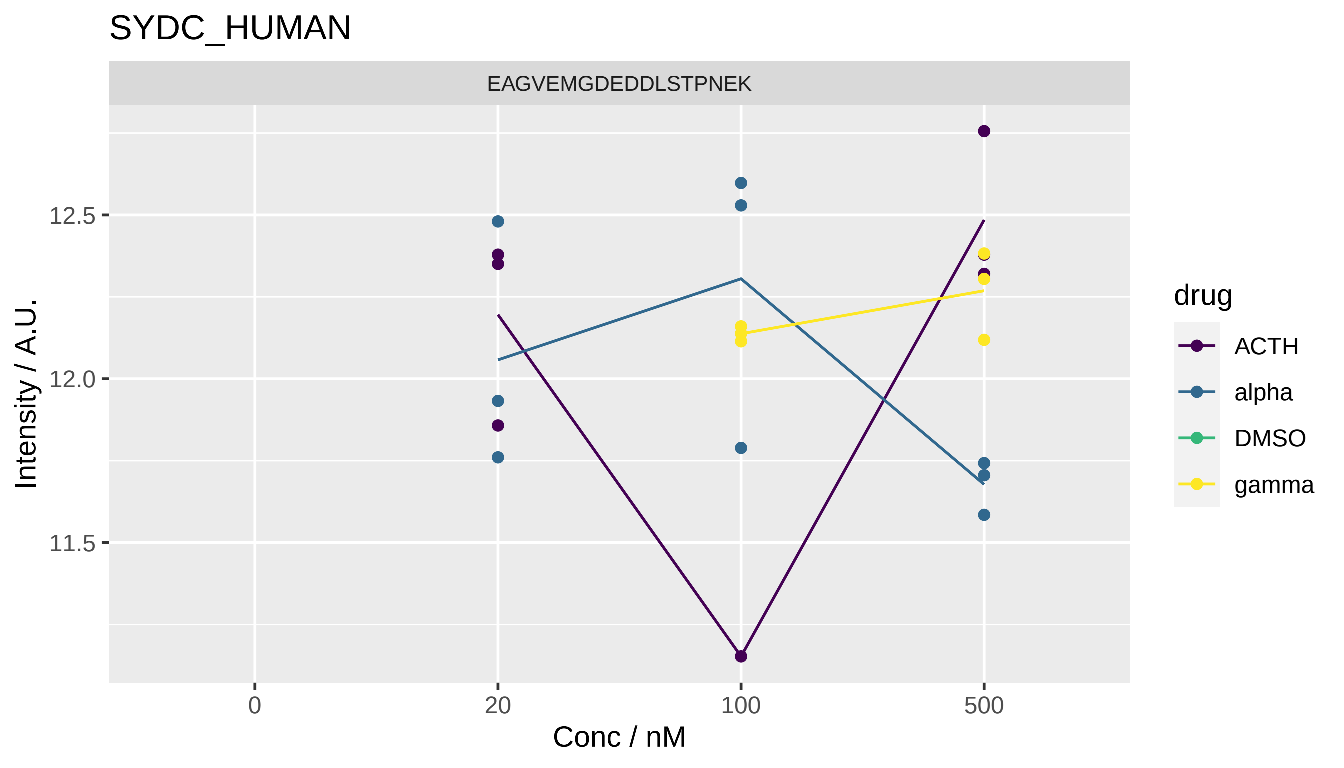

SYDC_HUMAN
EAGVEMGDEDDLSTPNEK
12.5
12.0
11.5
Intensity / A.U.
0
20
100
500
Conc / nM
drug
ACTH
alpha
DMSO
gamma

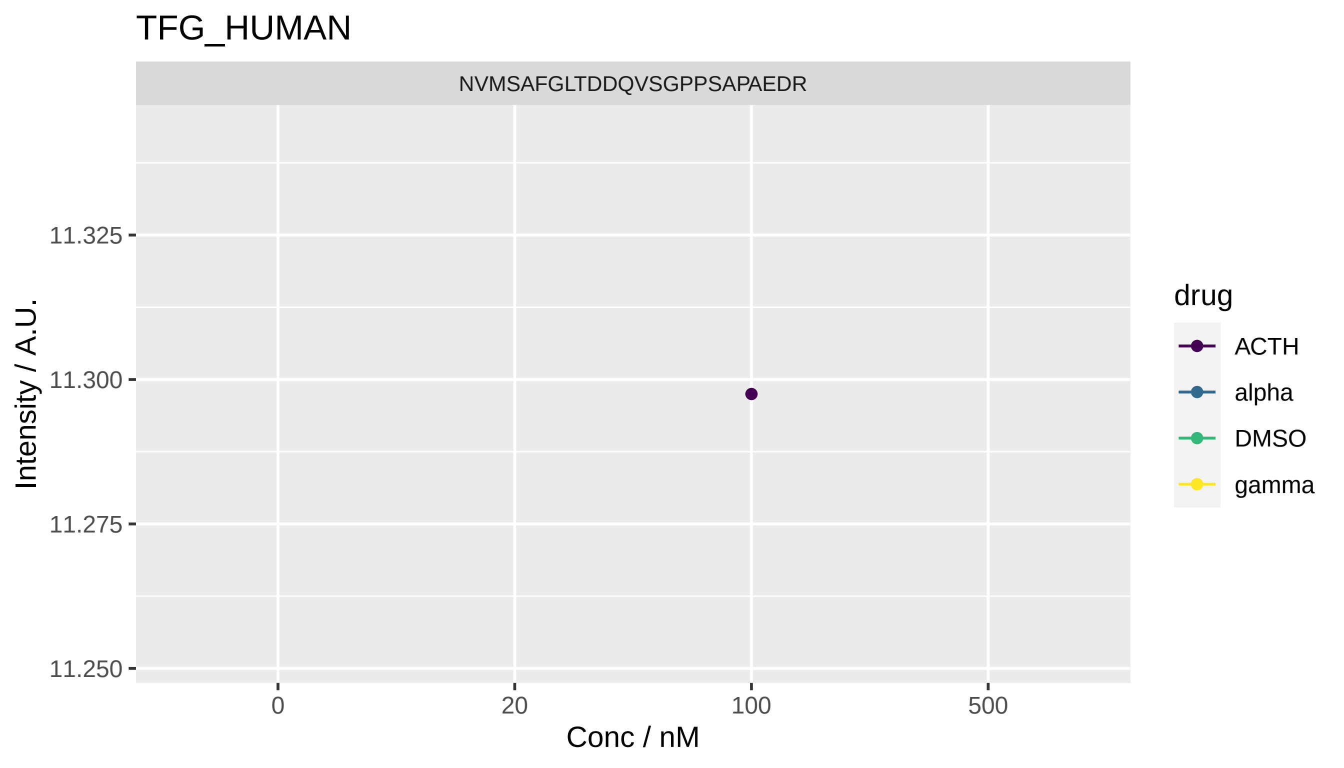
TFG_HUMAN
NVMSAFGLTDDQVSGPPSAPAEDR
Intensity / A.U.
11.325
11.300
11.275
11.250
0
20
100
500
Conc / nM
drug
ACTH
alpha
DMSO
gamma

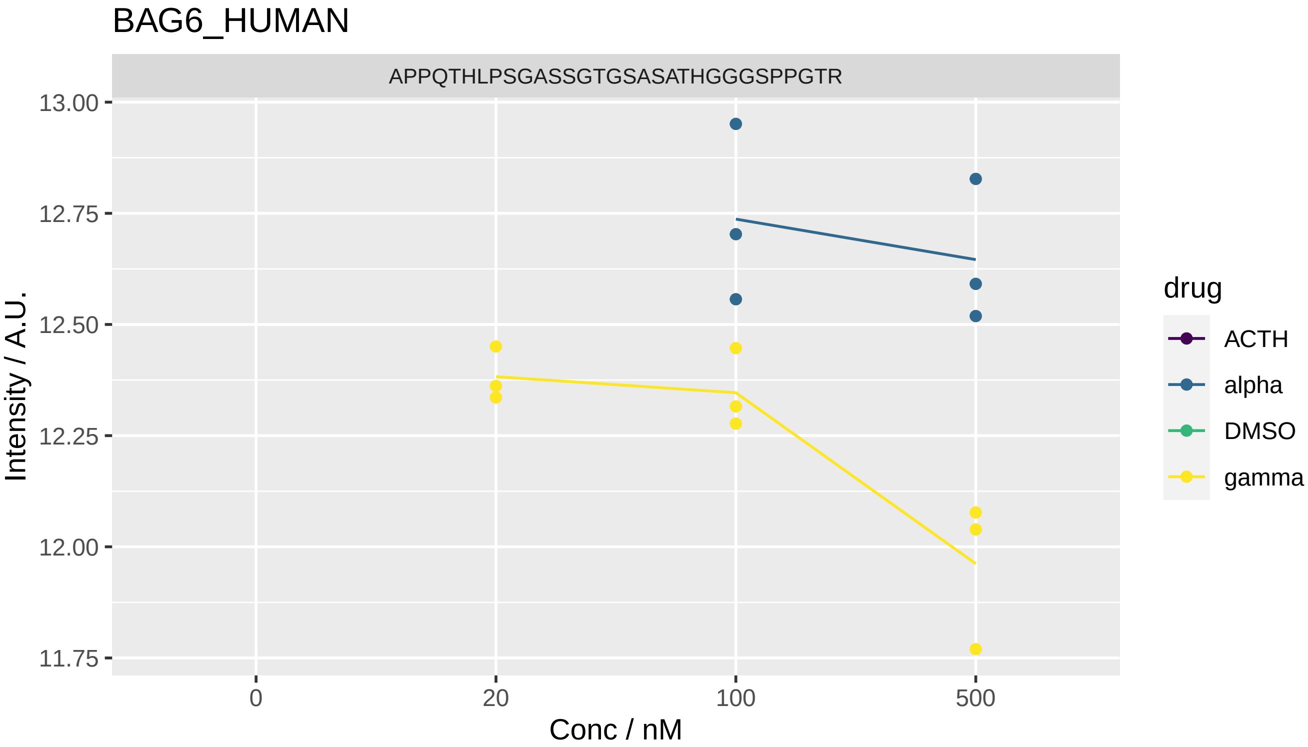

BAG6_HUMAN
APPQTHLPSGASSGTGSASATHGGGSPPGTR
Intensity / A.U.
13.00
12.75
12.50
12.25
12.00
11.75
Conc / nM
0
20
100
500
drug
ACTH
alpha
DMSO
gamma

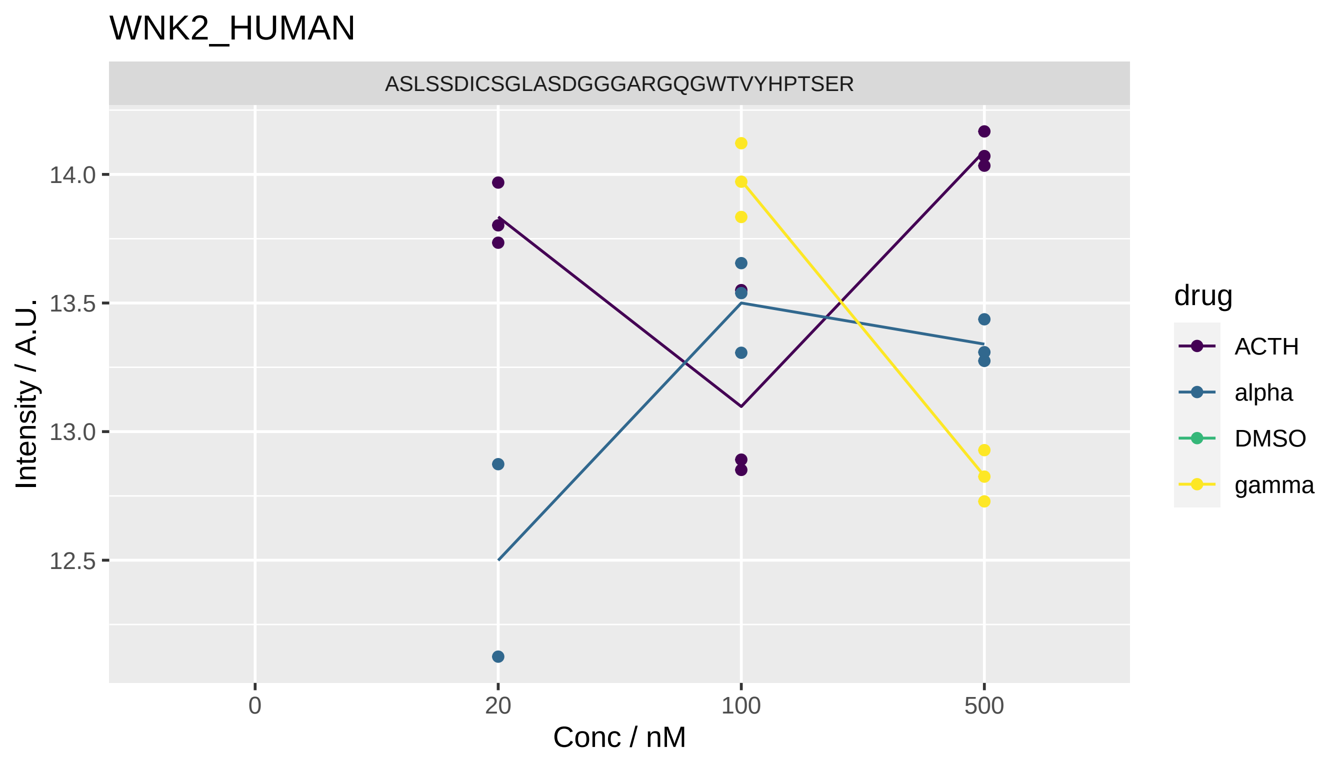

WNK2_HUMAN
ASLSSDICSGLASDGGGARGQGWTVYHPTSER
14.0
13.5
13.0
12.5
Intensity / A.U.
0
20
100
500
Conc / nM
drug
ACTH
alpha
DMSO
gamma
